# DIVIS: a semantic DIstance to improve the VISualisation of heterogeneous phenotypic datasets

**DOI:** 10.1186/s13040-022-00293-y

**Published:** 2022-04-04

**Authors:** Rayan Eid, Claudine Landès, Alix Pernet, Emmanuel Benoît, Pierre Santagostini, Angelina El Ghaziri, Julie Bourbeillon

**Affiliations:** 1 Institut Agro, Univ Angers, INRAE, IRHS, SFR QuaSaV, Angers, 49000 France; 2 Institut Agro, Angers, 49000 France

**Keywords:** Mixed datasets, Heterogeneous datasets, Phenotypic traits, Multivariate analysis, Ontologies, Semantic distance, Clustering, Visualisation

## Abstract

**Background:**

Thanks to the wider spread of high-throughput experimental techniques, biologists are accumulating large amounts of datasets which often mix quantitative and qualitative variables and are not always complete, in particular when they regard phenotypic traits. In order to get a first insight into these datasets and reduce the data matrices size scientists often rely on multivariate analysis techniques. However such approaches are not always easily practicable in particular when faced with mixed datasets. Moreover displaying large numbers of individuals leads to cluttered visualisations which are difficult to interpret.

**Results:**

We introduced a new methodology to overcome these limits. Its main feature is a new semantic distance tailored for both quantitative and qualitative variables which allows for a realistic representation of the relationships between individuals (phenotypic descriptions in our case). This semantic distance is based on ontologies which are engineered to represent real-life knowledge regarding the underlying variables. For easier handling by biologists, we incorporated its use into a complete tool, from raw data file to visualisation. Following the distance calculation, the next steps performed by the tool consist in (i) grouping similar individuals, (ii) representing each group by emblematic individuals we call archetypes and (iii) building sparse visualisations based on these archetypes. Our approach was implemented as a Python pipeline and applied to a rosebush dataset including passport and phenotypic data.

**Conclusions:**

The introduction of our new semantic distance and of the archetype concept allowed us to build a comprehensive representation of an incomplete dataset characterised by a large proportion of qualitative data. The methodology described here could have wider use beyond information characterizing organisms or species and beyond plant science. Indeed we could apply the same approach to any mixed dataset.

**Supplementary Information:**

The online version contains supplementary material available at (10.1186/s13040-022-00293-y).

## Background

The 2000s and the sequencing of complete genomes sparked a scientific revolution in the study of living beings. The now accessible no a priori approach results in the wider spread of high-throughput experimental techniques such as transcriptomics, proteomics, metabolomics, or phenomics and an increase in the volume of publicly available data. As a consequence, biologists are accumulating large amounts of datasets which are characterised by an increasing heterogeneity:

- information sources heterogeneity – multiple databanks, which can be local or distant, with various formats and interfaces, multiple file formats,
- data heterogeneity – various scales (from molecules to populations), types (quantitative and qualitative), modes (text or images), and structuring levels (database fields, structured text, free text).

Therefore the demand by biologists to integrate heterogeneous and large datasets from “omics” and phenotyping activities is rapidly expanding [1].

In this context where large complex datasets are becoming increasingly widespread, biologists often rely on multivariate analysis techniques to project individuals into a new coordinate space to get a first insight into the data and have smaller matrices to process. However, such approaches are not always easily achievable, in particular when faced with mixed (qualitative and quantitative) incomplete (that is to say, including missing values) datasets. Moreover, displaying large numbers of individuals leads to cluttered visualisations, with occlusions, which are difficult to interpret.

In this paper, we introduce a new methodology designed to overcome these limitations. The approach relies on a new semantic distance which is designed for both quantitative and qualitative variables and allows for a realistic representation of the relationships between individuals. This semantic distance is based on ontologies which are engineered to represent real-life knowledge regarding the underlying variables. We associate this new distance definition with an archetype concept to overcome the cluttered displays issue. We define archetypes as individuals representing groups of similar individuals from the dataset. Limiting the visualisations to these archetypes leads to a sparser representation which still provides valuable insight into the data.

More precisely, the structuring of the population in groups is conducted through clustering, for which numerous approaches exist [2, 3]. A common characteristic of clustering techniques is that they group individuals based on their similarity. This similarity is estimated based on distances between the features of the individuals.

However, most clustering methods rely on numeric arithmetic. Therefore the features have to be represented by numeric values. This causes problems with qualitative variables and even more in the case of mixed datasets. Some distances are designed to cope with qualitative data, for instance Jaccard’s coefficient [4], Dice’s coefficient [5], Gower’s distance [6], or the Chi-Square [7]. These metrics are widely used in biology, and in particular in ecology, to characterise species populations. For example *Pandey et al* rely on Jaccard’s coefficient to cluster sesame (*Sesamum indicum* L.) populations [8], Pavoine and colleagues extend Gower’s distance to characterise periurban woodland plant species populations [9]. Classical approaches often consist in the discretisation or dummy-coding of qualitative variables. But if the number of modalities is very different between variables the weight of each variable in the resulting similarity between individuals might be unbalanced [10]. *de Bello et al* propose a solution to overcome this issue for Gower’s distance [11]. For a review on current clustering approaches for heterogeneous data, see [12].

While methods to process qualitative variables exist they generally ignore the modalities’ inherent structure. For instance a variable corresponding to the months of the year can be considered as a circular variable. Pavoine and colleagues have made proposals to take such structuring into account in distance calculations through an extension of Gower’s distance [9]. But a lot of qualitative variables modalities are structured according to more elaborate schemes: it would be possible to describe such variables as ontologies[13]. Ontologies structure knowledge as graphs. Nodes represent concepts or terms and edges represent relationships between them. Ontologies are heavily developed and used in life sciences to annotate data, in particular in almost every biological database, and reason over domain knowledge [14].

In an ontology representing the modalities of a variable, modalities/values could be viewed as concepts. The complex links between these modalities would be materialised by the graph of relationships between concepts. We therefore propose to use the distance between concepts in corresponding ontologies to measure the distance between modalities of qualitative variables.

The measurement of distances in ontologies is a fundamental Semantic Web notion that is exploited for clustering, data mining or information retrieval [15]. Numerous formulas or algorithms [16, 17] exist to define such distances but most are based on two main approaches or a mix of the two.

- Edge-based approaches rely on counting the number of edges between two concepts in the ontology graph.
- Node-based approaches compare the properties of the concepts involved, be it the concepts themselves, their parents, or their children. They generally rely on the Information Content (IC) notion which evaluates how specific and informative a concept is.

However, these approaches rely on the graph topology, with no regard for what the concepts represent. This can lead to inaccuracies. For instance, a geographical ontology graph usually positions France, Italy and Denmark as three concepts that are part of Europe. A classical ontological distance calculation would lead to identical pairwise distances between these countries. This is false from a geographical point of view: Italy is closer to France than Denmark.

Therefore context dependent definitions for distances between modalities of a variable should be used:

- distance calculation algorithms in the corresponding ontology, in particular when the graph is large,
- distances based on real-life information such as in the geographical example above,
- ontology graphs augmented with expert-provided distance values associated with the relations between concepts.

Moreover, clustering and distance calculations usually cannot be performed as is on datasets including missing data. But data matrices in biology are often incomplete, for example because of the cost of some experimental techniques or because an individual hasn’t been available for the whole study. The first approach to coping with missing data is to exclude the affected individuals and variables. In our case study, this approach is inapplicable due to a high percentage of missing values. More than half the rows contain missing values and four out of eleven variables have more than 84% of missing values. A second approach relies on estimating missing values using imputation techniques. A review of these methods is available in [18] in the field of epidemiology. However, using imputation techniques depends on the data at hand. In trait datasets, Johnson and colleagues [19] show that estimating missing data is not always appropriate. For our case study, the traits are sometimes different between individuals of the same category. Therefore, we chose to ignore the missing values and to define a distance based on the available data only.

To reduce the number of individuals displayed in the visualisations we also propose to represent each cluster by a limited number of individuals we call archetypes. In order to define these archetypes different strategies can be considered depending on the clustering results.

- In the case of a large number of small clusters, representing each group by a single individual is probably adequate. In such a case we can imagine basing the archetypes definition on the cluster centroids.
- In the case of a small number of large clusters a single individual might not be sufficient to represent the intra-cluster diversity. In these conditions, it seems more appropriate to select several individuals with one of the existing sampling techniques [20, 21].

A visualisation of the archetypes allows to declutter the initial display of the population. Means to switch between archetypes and whole groups display should be provided.

In this paper, we introduce a new semantic distance to cope with the highlighted limitations. We compare it with a classical distance used for mixed datasets. These distances are incorporated into a complete pipeline, from raw data file to visualisation, that we present here. As a proof of concept, we apply it to a rosebush dataset which includes passport data and a collection of qualitative and quantitative phenotypic traits.

## Methods

### Use case: rosebushes phenotypic traits

We illustrate our study with information on rosebush varieties associated to the DNA collection of the Pome Fruits and Roses Biological Resource Centre (BRC) in Angers, France. The dataset consists in passport and phenotypic data assessed and/or gathered from various sources during the study of French roses (*Rosa* sp.) performed by *Liorzou et al.* [22]. It includes 1434 rosebushes from European garden roses from the 18^*th*^ and 19^*th*^ centuries. Most of these rosebushes correspond to different varieties. Each rosebush is described by the variables listed in Table 1. All the variables are qualitative, except for the number of flowers, and field experts defined all the modalities. The dataset is far from complete. The “Quantity of prickles”, “Perfume intensity”, “Repeat flowering level” and “Number of flowers by volume” variables are only known for a small number of rosebushes. Only one or two variables are known for some of the individuals.

**Table 1:** Variables in the rosebush dataset

Variable	Type	Nature	Number of modalities
Horticultural group	Passport	Qualitative	17
Geographic origin	Passport	Qualitative	9
Breeding period	Passport	Qualitative	16
Ploidy level	Passport	Qualitative	5
Petal colour	Phenotypic	Qualitative	12
Bush height	Phenotypic	Qualitative	6
Quantity of prickles	Phenotypic	Qualitative	4
Perfume intensity	Phenotypic	Qualitative	2
Repeat flowering level	Phenotypic	Qualitative	6
Number of flowers by volume	Phenotypic	Quantitative	-
Duplicature type	Phenotypic	Qualitative	4

Passport variables come from [22] or are inferred from them. The horticulture group is defined according to the American Rose Society (ARS) classification. Breeding dates are grouped into time periods. Phenotypic variables have been evaluated by the Pome Fruits and Roses BRC and its partners: breeders and rose gardens

Such a dataset could be difficult to analyse with classical approaches. We chose it to test whether our method could provide new insight into the data.

**Fig. 1:**
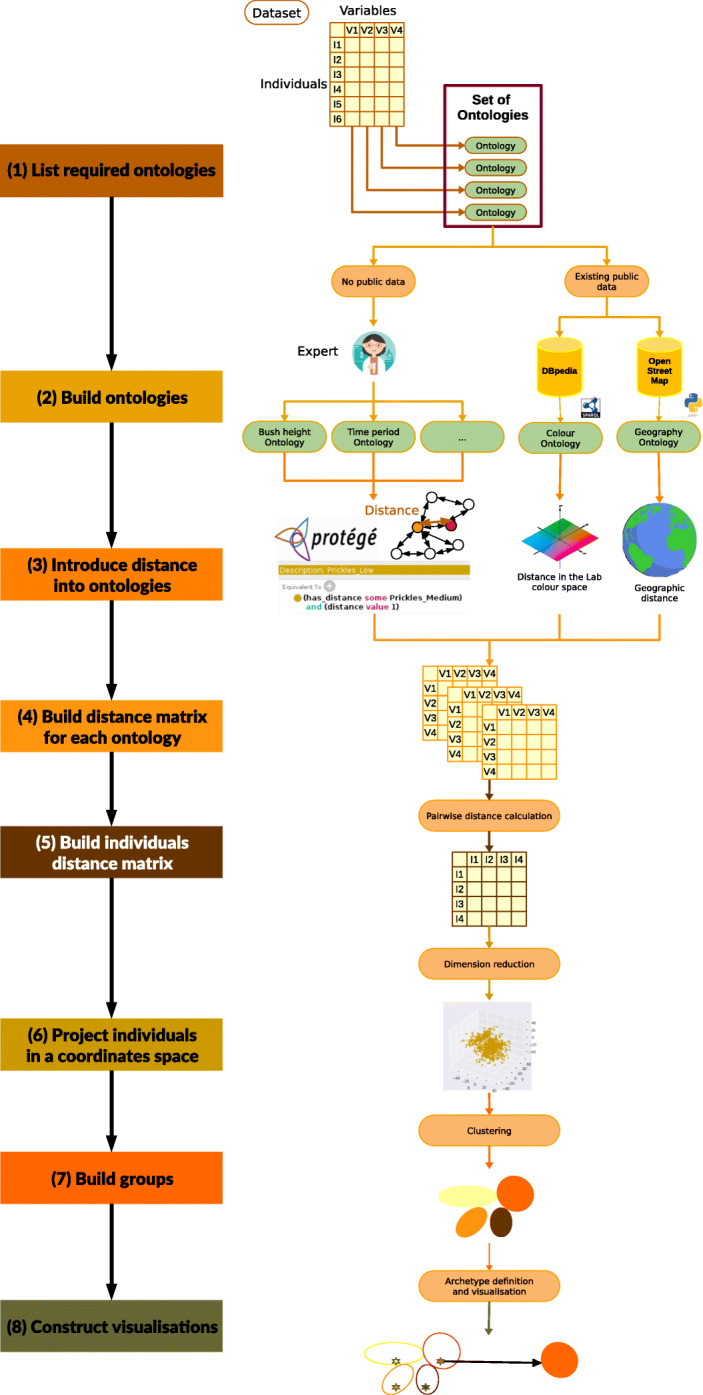
The dataset processing pipeline. Based on the list of qualitative variables we define the list of required ontologies (one for each variable). For each ontology, if relevant data are publicly available we retrieve it. Otherwise, we rely on expert knowledge to build the ontology graph. We introduce distances between concepts in the ontologies based either on real-life distances or expert knowledge. These ontologies including distance between concepts information are used to build a distance matrix between variable modalities for each qualitative variable. Based on the vector of variable values which represent each individual in the dataset we calculate pairwise distances to build a distance matrix between individuals. Individuals are then projected in a coordinate space using dimension reduction methods. Individuals coordinates are used during the clustering process to build groups. Representative individuals for each group are estimated to define the groups’archetypes which are used as part of the visualisations

The dataset was therefore subjected to the pipeline presented in Fig. 1. This pipeline was developed in *Python 3.7*. It relies mainly on the *NumPy* [23] and *pandas* [24] libraries to manipulate the data, *scikit-learn* [25] to perform machine learning, and *matplotlib* [26] and *seaborn* [27] to draw the figures. Traces of the whole process are logged using the *logging* library and parameters of an individual run are defined by the user in a configuration file in YAML format.

The following subsections detail it more precisely.

### Building ontologies and capturing the distance between concepts

#### General principle

In the first stages of the process, we associated each qualitative variable in the dataset with an ontology. This corresponds to steps (1) and (2) from the pipeline in Fig. 1. The various modalities of a variable then became concepts in an ontology. If an ontology corresponding to the variable was publicly available, we used and adapted it to fit our variable modalities as necessary. Otherwise we had to rely on expert knowledge to transform the list of modalities into the concept graph of an ontology.

We have to define a pairwise distance for each pair of concepts in each ontology, as indicated in step (3) of the pipeline in Fig. 1. If the distance between modalities can be calculated based on what they actually represent, as is the case for geographical areas, we use that calculation. If the ontology graph is small enough, we can consider augmenting it using expert-provided distance estimations which will be stored along with the ontology graph. Otherwise, when the graph is large or if no extra expert knowledge is required, we fall back to using existing algorithms to calculate a distance between concepts.

According to these principles the qualitative variables in our dataset were handled as follows, as for step (4) of Fig. 1.

#### Variables associated with public ontologies

Public ontologies existed for colours and geographic information. These ontologies could suitably represent our “Petal colour” and “Geographic origin” variables.

Our code extracts colours’ descriptions from DBpedia [28], using its SPARQL endpoint, then performs lookups based on their names. DBpedia descriptions include coordinates of reference in different colour spaces. We chose to use the L*a*b* colour space because it is designed to approach the perception of colours by human vision. In this space, *L*∗ represents perceptual lightness, *a*∗ the green–red opponent colours, and *b*∗ the blue to yellow tones. We used *ΔE* (CIE 2000), which quantifies the visual difference between two L*a*b* colours and is presented in Eq. (1), as the distance between colours.

1 $$ \Delta E = \sqrt{({L_{2}}^{*} - {L_{1}}^{*})^{2} + ({a_{2}}^{*} - {a_{1}}^{*})^{2} + ({b_{2}}^{*} - {b_{1}}^{*})^{2}}   $$

We relied on the implementation of *ΔE* provided by the *colormath* Python library [29].

We used the *GeoPy* library [30] and the Nominatim geocoder to access OpenStreetMap data [31] and map regions of origin to coordinates. Some region names in our dataset do not exist in OpenStreetMap. It is for example the case for the subdivision of France into four main quadrants. In such cases, we considered the list of named areas composing the region and associated it with the mean latitude and the mean longitude of the areas in the list as a proxy for its location.

#### Variables with no associated public ontology

For the other variables, no existing ontology could be located. The structuring in the form of a graph of possible values was carried out for each variable in collaboration with rosebush experts and stored in an ontology file in OWL format using the Protégé editor [32]. We then defined a distance between pairs of concepts in each graph.

Time periods, which do not overlap in our case, were estimated based on their median year (which is equal to the mean value here). One could consider these time periods as “confidence intervals” around the estimated year. If *S*1 and *S*2 are the start years of two periods and *E*1 and *E*2 the end years, we defined the distance between the two periods *Δt* as the number of years between the median of each period as presented in Eq. (2).

2 $$ \Delta t = \left\lfloor\left|\left(S1 + \frac{E1 - S1}{2}\right) - \left(S2 + \frac{E2 - S2}{2}\right)\right|\right\rfloor   $$

Among the time period modalities, two have just one date: “ <1700” and “ >1920”. We considered 1600 as the start date for the first one and 2020 as the end year for the second.

**Fig. 2:**
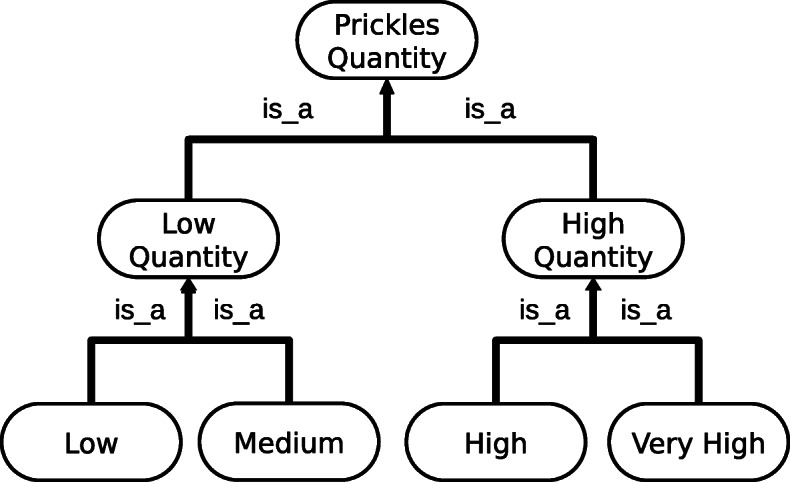
Ontology graph for the “Quantity of prickles” variable

Ontologies representing the other phenotypic variables and distances between their modalities were defined with the help of rosebush experts. Resulting ontologies were usually quite small and organised as trees. Moreover, most of these variables were ordinal. For instance, modalities for the “Quantity of prickles” variable (“low”, “average”, “high” and “very high”) can be ordered from the lowest quantity to the highest. In the ontology, these modalities are organised in two subgroups as presented Fig. 2. Therefore, we considered it relevant to hand-tailor the distances between pairs of leaf concepts to better capture expert knowledge. These distances were defined with arbitrary but not random values: we chose values so that inter-subgroups distances are higher than distances within a subgroup and so that the original order is conserved. The resulting distance matrix for this example is presented in Table 2.

**Table 2:** Distance matrix for the quantity of prickles ontology

	Low	Medium	High	Very high
Low	0	1	5	6
Medium	1	0	4	5
High	5	4	0	1
Very high	6	5	1	0

**Fig. 3:**
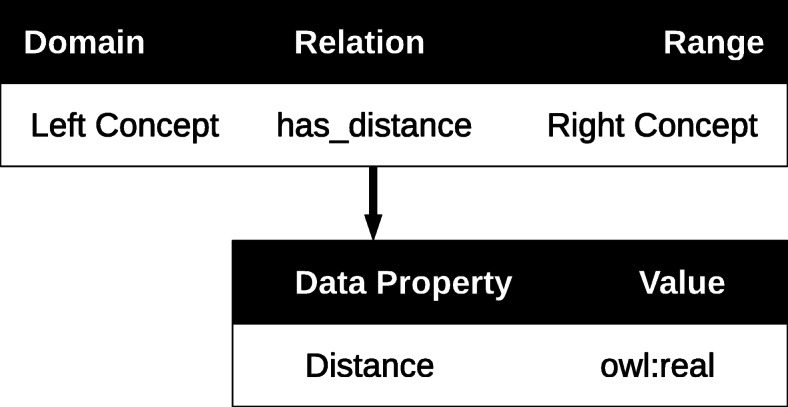
The principle used to store a distance between concepts in the OWL ontology

At that point we needed to store values for the pairwise distances between concepts in the OWL ontology. We introduced a has_distance relationship as an Object Property. We associated it with a distance Data Property of type owl:real. This distance acts as the Range of has_distance. This principle is illustrated in Fig. 3.

**Fig. 4:**
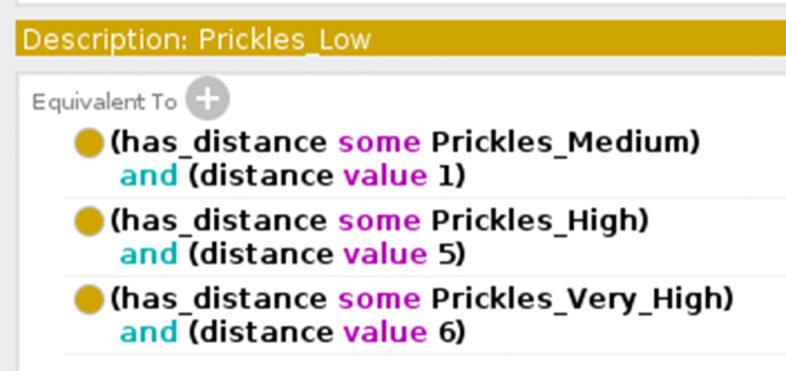
Quantity of prickles ontology - representation of the distances between the “Low” concept and the others

Considering the previous example the distances between the “Low” concept and the others in the “Quantity of prickles” ontology are represented Fig. 4.

#### Building distance matrices for the ontologies

In order to build the distance matrix for the colour and region ontologies, each pair of concepts in the ontology file is processed and the distance is calculated according to the previously defined methods. The OWL file containing the ontologies we engineered is read using the Python *Owlready2* library [33]. We thus retrieve the list of concepts for each ontology along with the pairwise distances. These are formatted as distance matrices stored in a global Microsoft Excel file.

The ranges of distance values for each variable are very different. Each distance matrix is normalised on a [0−100] scale to prevent some variables from out-weighting the others in future calculations.

### Building individuals distance matrix

The next step in Fig. 1, that is to say step (5), consists in building the individuals distance matrix. We had to define how to calculate the pairwise distance.

**Fig. 5:**
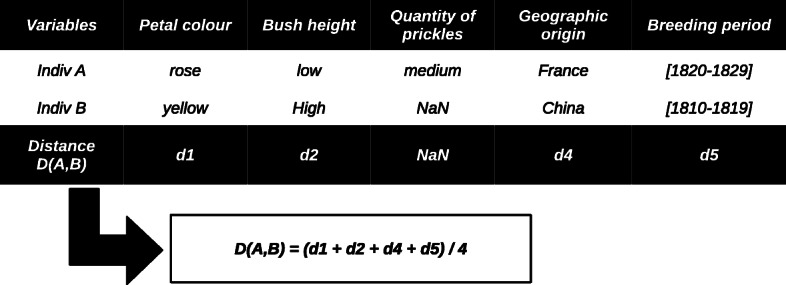
Example of a calculation of distance between individual rosebushes

Each individual can be represented as a vector of variable values. If we consider two individuals denoted by the *A* and *B* vectors, the values of the *i*^*th*^ variable correspond to *A*_*i*_ and *B*_*i*_ respectively. The distance *d*_*A*,*B*_(*i*) between *A* and *B* for the *i*^*th*^ variable can be found in the corresponding distance matrix as the distance between *A*_*i*_ and *B*_*i*_. The distance *D*(*A*,*B*) between *A* and *B* can therefore be expressed as Eq. (3).

3 $$ D(A,B) = \frac{1}{\sum_{i\in M}w(i)} \sum_{i\in M} w(i) d_{A,B}(i)   $$

where *w*(*i*) is the weight of the *i*^*th*^ variable, and *M* is the set of variables for which a distance can be defined. Indeed we have missing data in our dataset. If either *A*_*i*_ or *B*_*i*_ or both are missing then *d*_*A*,*B*_(*i*) is missing too. The weight for each variable is defined in a configuration file. An example for a subset of variables and equal weights is presented in Fig. 5.

The computation is made for all pairs of individuals to build the final distance matrix. This matrix contains both the *D*(*A*,*B*) distance and the number of variables used in the calculation (i.e. cardinal of *M*) for each pair of individuals.

We decided to also calculate a distance matrix based on Gower’s distance [6] for comparison purposes. We could not find an implementation of this distance in Python libraries. Moreover, we needed it to process missing values the same way as our semantic distance. We therefore developed our own based on the Dice and Manhattan distances as found in the *scikit-learn* library. The algorithm iterates over all variables in the dataset and builds a distance matrix for each. The Manhattan distance is used if the variable is quantitative. Otherwise it converts it into binary indicator variables including one for missing values. It calculates the Dice distance on the new dummy variables and marks as missing the pairwise distances flagged by the missing value indicator. The final distance matrix is calculated as the by-element average of all the individual variables’ distance matrices.

### Projection in coordinate space and clustering

The next stage is to group similar individuals based on the distance matrix. Since different clustering algorithms can produce different results depending on the structure of the population to classify, we chose to test several algorithms. However, not all clustering algorithms can use a distance matrix as input.

We therefore had to perform a dimension reduction [34] to project the distance matrix in a coordinate space and use the projection as input for all clustering algorithms, as indicated in step (6) of the Fig. 1 pipeline. Numerous methods exist [35] and we considered the following:

- Classical Multi-Dimensional Scaling, also known as Principal Coordinates Analysis, as implemented in the *scikit-bio* Python library [36], which solves a symmetric eigenvalue problem (PCoA),
- Metric Multi-Dimensional Scaling as implemented in the *scikit-learn* Python library [25], which uses an iterative minimisation procedure (mMDS),
- Laplacian eigenmaps, as implemented in the *scikit-learn* Python library [25].

The intrinsic dimensionality of a PCoA is usually associated with the drop-out point of the eigenvalues. The same approach can be used for the Laplacian eigenmaps. The *scikit-learn* MDS function provides a value of *STRESS* that quantifies the quality of the representation. This indicator can be normalised to obtain the “Kruskal stress” (*stress1*), defined in Eq. (4):

4 $$ stress1= \sqrt{\frac{\sum_{j>i}(\hat{\delta}_{ij} - \delta_{ij})^{2} }{\sum_{j>i}({\delta}_{ij})^{2}}}   $$

where *δ*_*ij*_ corresponds to the observed distance between pairs of individuals (*i*,*j*) supplied as input to the multidimensional positioning algorithm and $\hat {\delta }_{ij}$ is the reconstructed distance in the Euclidean space representing the data. *stress*1 is a widely used indicator in the literature [34] and thresholds exist to guide the selection of the number of dimensions to keep in the new space to have a sufficiently good representation.

Regarding the clustering *per se*, that is to say step (7) from the pipeline in Fig. 1, we sought to compare the results of various algorithms and relied on the *scikit-learn* implementation of the following algorithms:

- Birch [37],
- Gaussian Mixture [38],
- Hierarchical Clustering with Ward linkage [39],
- KMeans [40],
- KMedoids [41],
- Spectral Clustering [42],

These algorithms require the number of clusters as a parameter. In order to assist with this choice, we performed a Silhouette analysis [43] using the *scikit-learn* implementation of the Silhouette coefficient calculation.

We diverted classification evaluation approaches to compare the results of the various clustering algorithms. Indeed these evaluation techniques usually compare a prediction with a ground truth. We didn’t have a ground truth but the results of several algorithms. We considered the KMeans clustering results as “ground truth” and the results of each other algorithm as predictions. We then computed concordance matrices using *pandas* and confusion matrices using *scikit-learn*.

We used the same approach to compare the clusters between the two distances.

### Archetypes definition and visualisation

The next stage (8) of the process described in Fig. 1 consists in representing each group by a small number of individuals. Two strategies were implemented.

- **Single archetype** – In the case of a large number of small clusters, each group is represented by a single individual. This individual is chosen by identifying the cluster’s centroid as implemented by the *scikit-learn* library then selecting the individual closest to the centroid by Euclidian distance.
- **Multiple archetypes** – In the case of a small number of large clusters, each group is represented by several individuals. 5% of the population in each cluster is sampled at random using the *pandas* library.

We relied on the *seaborn* library to output visualisations of the results. Since the number of dimensions may be superior to three, we chose to draw scatterplot matrices. Given scatterplot matrices are symmetrical along the diagonal, we used each half to display two different visualisations of the results.

- The “regular” representation with all individuals appears in the **bottom-left corner**. This representation is used in classical dimension reduction methods and users are accustomed to this type of visualisation.
- Our proposal to reduce cluttering is displayed in the **top-right corner** of the scatterplot matrices. This representation only includes the archetypes as well as kernel density estimations. It provides a rough estimate of the group envelopes.

Each cluster is associated with a colour. The underlying colour map can be defined as a parameter in a configuration file. We picked colours tailored to most colour vision deficiencies [44] to generate the figures for this paper.

In the bottom-left part of the matrix, the number of displayed individuals is large. We made the dots transparent in order to reduce the occlusion and provide insight into the density of the points.

Moreover, the pairwise distances between individuals are calculated based on different numbers of variables. In this context, we considered that a distance calculated based on more variables would be more accurate. We chose to represent individuals with more missing values by larger dots as a proxy to this uncertainty in distances calculations.

## Results

### Classical analysis: MCA

We started by exploring a classical analysis approach before applying the pipeline presented here. Given the types of the variables in the dataset, a Multiple Correspondence Analysis (MCA) [45] was seemingly appropriate after removing the only quantitative variable “number of flowers by volume”. Indeed this variable has around 99% missing values.

We applied MCA with the *prince* Python package [46], after removing all missing values (1029 individuals removed out of 1434). We present in Table 3 the percentage of inertia explained by the first six components. With the first two components, the total inertia was 7.5*%* and reached 19.2*%* with six components. Therefore the projection in the MCA space was very poor.

**Table 3:** MCA explained inertia by component for the rosebush dataset without missing data

Component	1	2	3	4	5	6
Explained inertia	0.041	0.034	0.032	0.029	0.028	0.028

Another point that one needs to be careful about in MCA is the presence of rare categories (categories of small size). These categories can affect the results since the associated inertia will be high. Several solutions can be considered to remedy this. In particular, one can group the categories if there are natural groupings. We increased the inertia of the axes of the MCA by both removing missing values and grouping categories. This percentage reacheed 12.2*%* for two components and 30.8*%* with six components. However, grouping modalities is not relevant for all variables. For instance grouping underrepresented flower colours led to very different colours being treated together and close colours being distinguished.

In the end, this attempt to use an MCA approach was not conclusive for our objective and for our type of data (i.e. structured data).

### Distance matrices

**Fig. 6:**
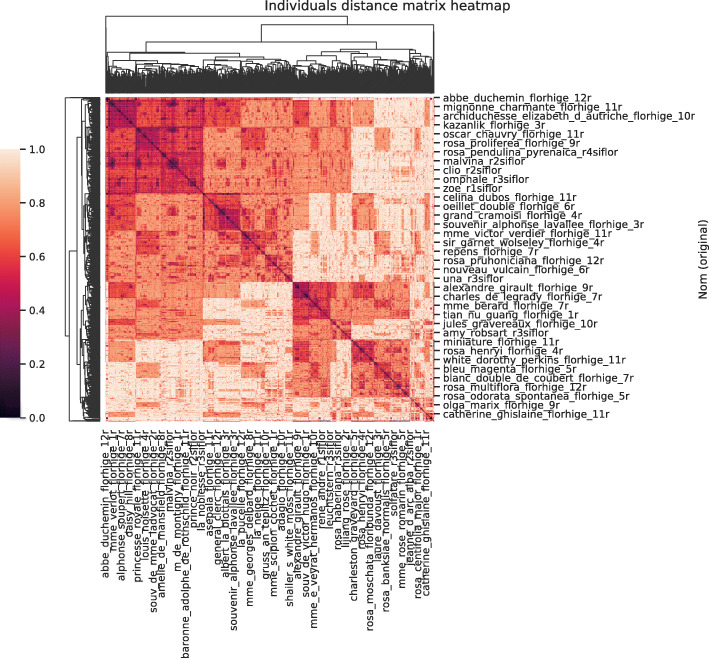
Distance matrix between rosebushes - Gower distance

**Fig. 7:**
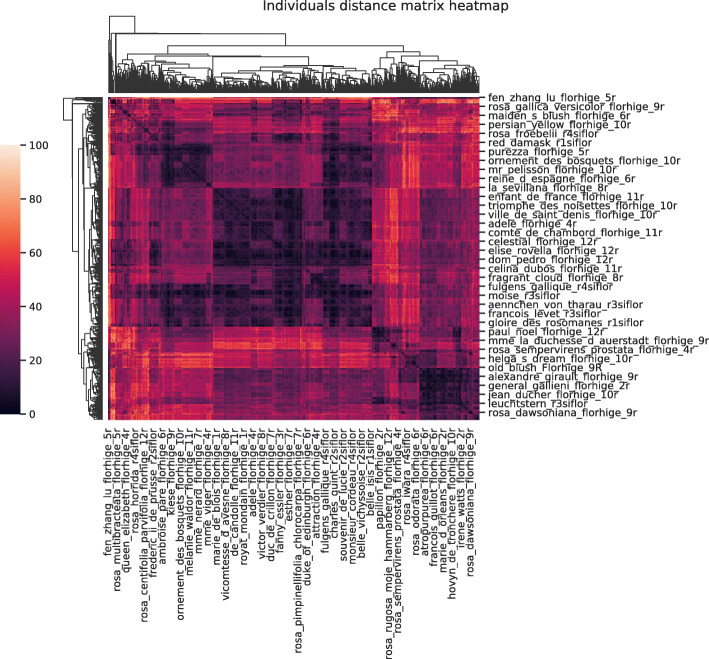
Distance matrix between rosebushes - Semantic distance

Our dataset was then subjected to the pipeline from Fig. 1. We first of all produced distance matrices between individuals using both distances: Gower’s and our semantic one. These matrices are displayed as heatmaps in Figs. 6 and 7.

**Fig. 8:**
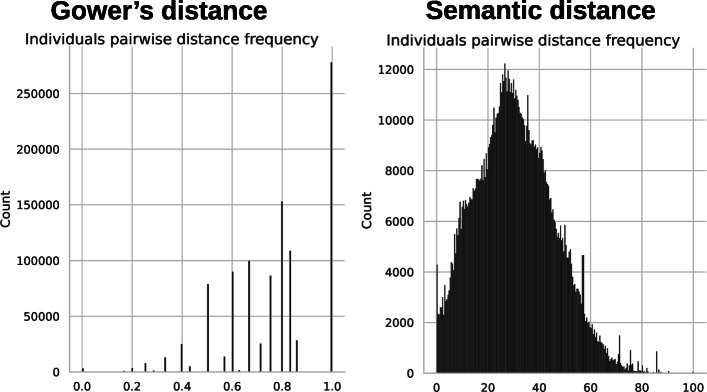
Frequency of distance values in the individuals distance matrices for Gower’s distance and our semantic distance

The two matrices are quite different. Gower’s matrix has a larger proportion of values closer to the maximum compared to the semantic matrix. This can be explained by the way the two distances are constructed. The distance between individuals is based on a majority of qualitative variables and a single quantitative variable. The quantitative variable is associated with a very limited amount of data. In Gower’s case, we mainly represent the proportion of variables whose values are different between individuals. Indeed qualitative variables are somewhat “interchangeable” given the distance between two individuals is binary. The values in the distance matrix are often superior to 0.5 because our rosebushes don’t share a large proportion of values and we necessarily have a distance equal to one for each variable where the values are different. In the semantic case, the distance values between modalities depend on the variables and are not equal to one. Therefore, the range of possible distances between individuals is quite large but with a smaller maximum. The frequency of values in the two cases presented in Fig. 8 illustrates this discussion.

Looking at the heatmaps from Figs. 6 and 7, both distances seem to structure the population in 3 or 4 groups but the interpretation is less clear between the two larger groups in the semantic distance case.

### Projection in a new coordinate space

**Fig. 9:**
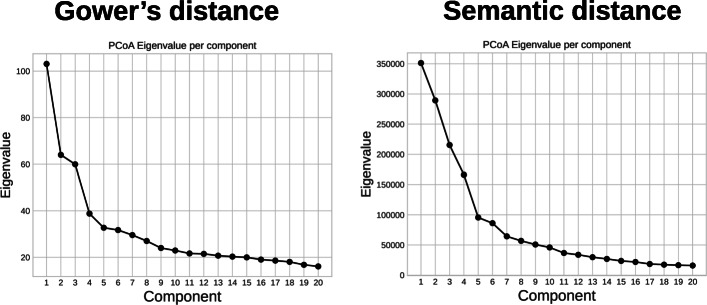
Eigenvalues according to the number of components for the PCoA. Distance matrix based on Gower’s distance (left) and semantic distance (right)

**Fig. 10:**
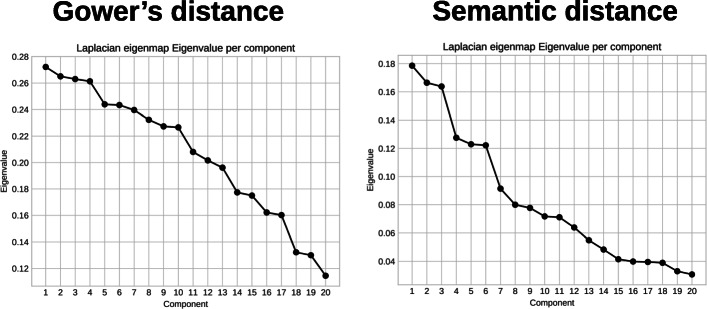
Eigenvalues according to the number of components for the Laplacian Eigenmaps. Distance matrix based on Gower’s distance (left) and semantic distance (right)

**Fig. 11:**
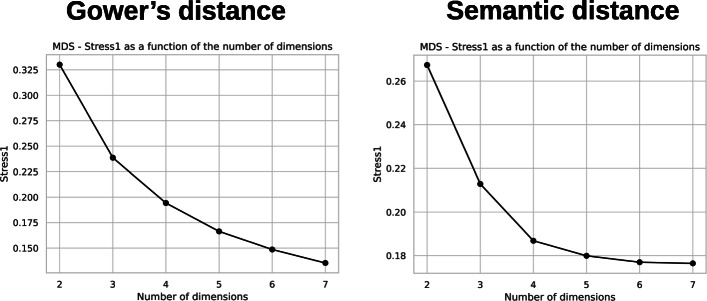
Value of *stress*1 according to the number of dimensions for the metric MDS. Distance matrix based on Gower’s distance (left) and semantic distance (right)

We performed the dimension reduction for both matrices and all three techniques: PCoA, mMDS and Laplacian eigenmaps. We then evaluated the intrinsic dimensionality for the dimension reduction techniques: we calculated the eigenvalues of the PCoA and the Laplacian eigenmaps and plotted their values for an increasing number of components as presented Figs. 9 and 10, respectively. In this type of representation, the number of components is usually chosen at the bend in the curve. In the PCoA case, this would be 4 for the matrix based on Gower’s distance and 5 for the matrix based on the semantic distance. For the Laplacian eigenmaps, these values would be 4 and 3 respectively. For the mMDS we plotted the value of *stress*1 from Eq. 4 for an increasing number of axes, as presented in Fig. 11. The *stress*1 values are similar for both distances. It is generally admitted that a *stress*1 value below 0.2 corresponds to a good representation of the distance matrix in a coordinate space [34]. We thus chose four dimensions for all dimensionality reduction techniques and for both distances to easily compare the results.

The points in the new spaces appear less spread for Laplacian eigenmaps than for the others [see Additional files 1 and 2 for the final visualisations for Laplacian eigenmaps and PCoA dimension reductions respectively]. Moreover, the results are relatively close between PCoA and mMDS. Therefore, we chose to focus the remainder of the paper on the results of the mMDS since it has the same intrinsic dimensionality for both distances.

Data points also appear more spread for the semantic distance than for Gower’s distance. It is related to the fact that we have a wider range of distances between individuals.

### Number of clusters choice

A required parameter for all tested clustering algorithms is the number of clusters to build. We therefore performed a Silhouette analysis as a preliminary step for the clustering process, for both distances and using two strategies:

- Plot the mean Silhouette coefficient as a function of the number of groups for three clustering algorithms: KMeans, KMedoids, and Hierarchical Clustering,
- Perform a Silhouette analysis at the individuals level for an increasing number of clusters for the KMeans algorithm.

**Fig. 12:**
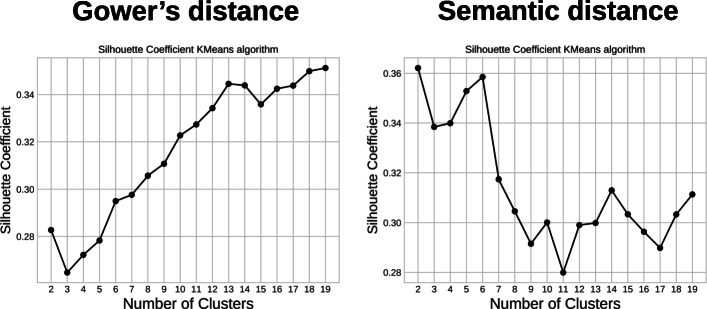
Mean Silhouette coefficient as a function of the number of clusters, KMeans algorithm. Gower’s distance (left) and semantic distance (right)

The mean Silhouette graphs for the KMeans algorithm are presented in Fig. 12.

**Fig. 13:**
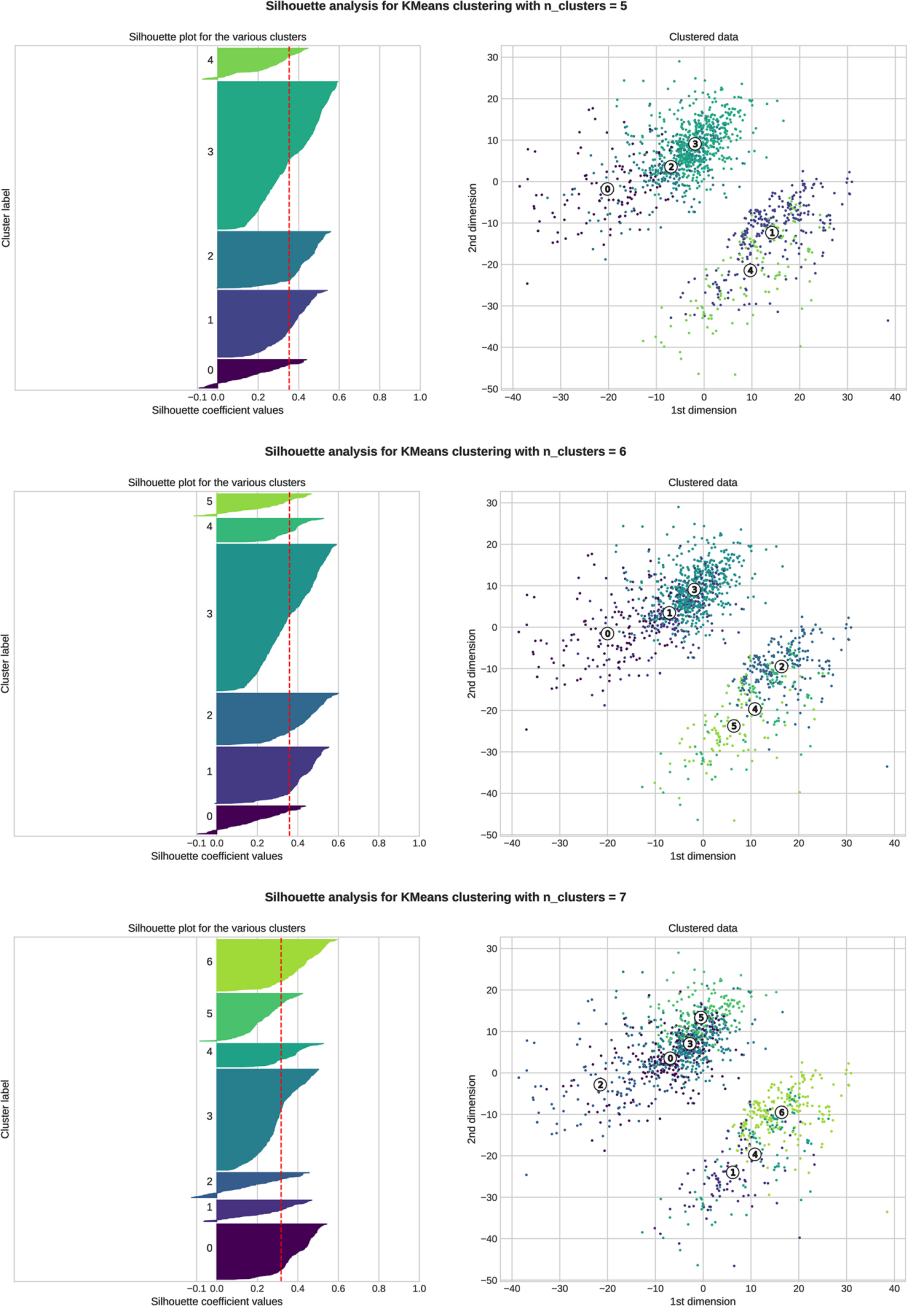
Silhouette analysis for 5, 6, and 7 clusters, KMeans algorithm, semantic distance. The left-hand part of each figure presents the Silhouette value for each individual (colour coded per cluster) and the mean Silhouette value as the red dash vertical line. The right-hand part presents the individuals projected in the first two dimensions

For the semantic distance, the number of clusters that maximises the Silhouette coefficient is 6. Profiles are similar for the KMedoids and Hierarchical Clustering algorithms. Both algorithms suggest five to six clusters. A more precise rendering of Silhouette values at the individual level is presented for the KMeans algorithm with five to seven clusters in Fig. 13 [see Additional file 3 for renderings for 2 to 19 clusters]. This figure confirms that the clustering quality from a Silhouette point of view is similar, and we chose to perform the next steps for 5, 6, and 7 clusters.

For Gower’s distance, the situation is very different since it seems that the more clusters the better the representation [see Additional file 4 for Silhouette coefficient renderings for 2 to 19 clusters]. We chose to perform the following steps with five to seven clusters to compare both distances based on the same number of clusters.

### Cluster analysis

As part of the analysis of the clustering results, we wanted to:

- compare the results between the different clustering algorithms for each distance (Gower’s and semantic). In order to do so, we considered the KMeans results as ground truth and compared each of the other algorithms with it,
- compare the results of our new semantic distance with Gower’s for all algorithms. Here we considered the results for the semantic distance as ground truth.

We calculated concordance matrices for 5, 6, and 7 clusters and for the two sets of comparisons. These provided us with a mapping between the clusters built according to the two methods.

**Fig. 14:**
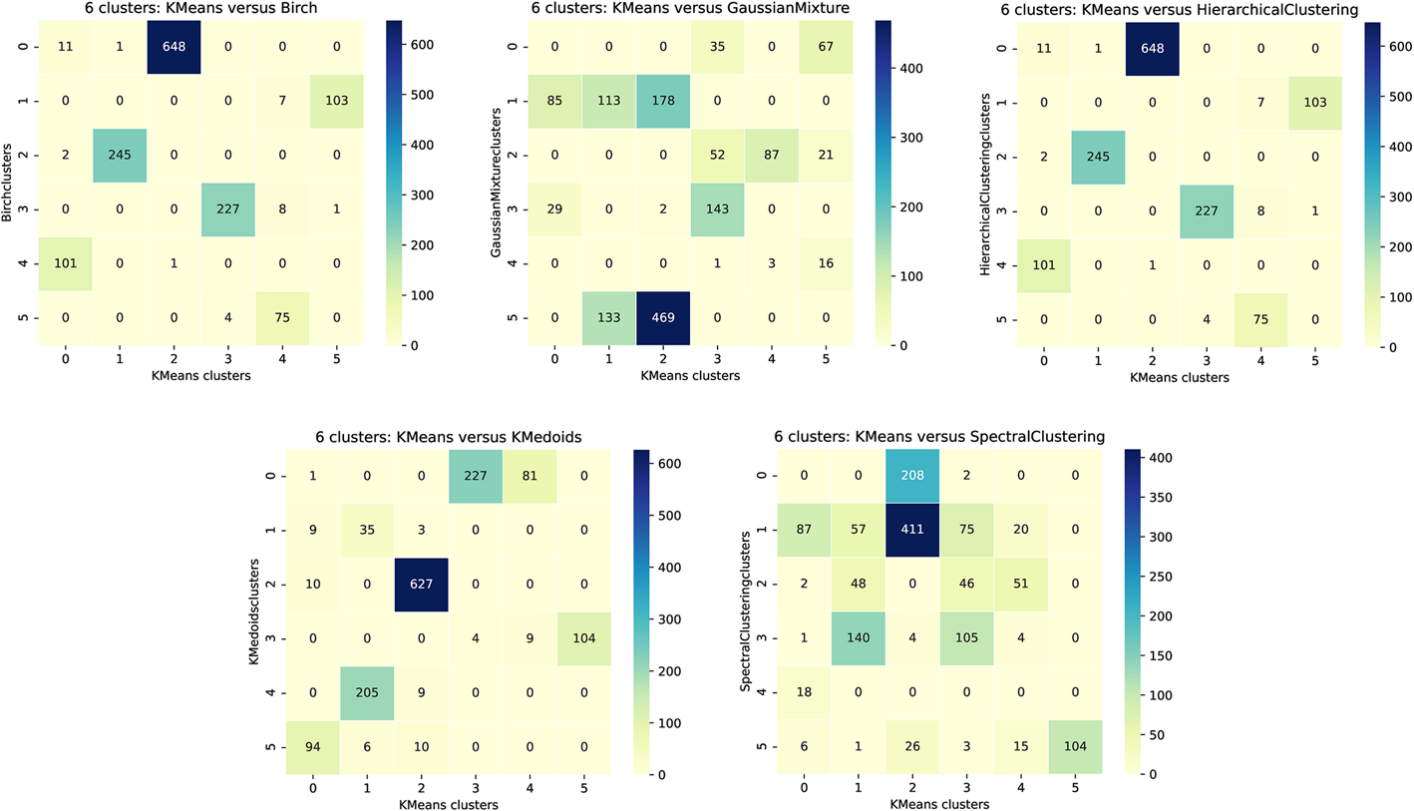
Heatmaps of the concordance matrices between KMeans clusters for 6 clusters and the other tested clustering algorithms for semantic distances. In each heatmap, columns correspond to the KMeans clusters and rows to the clusters for the other algorithm. This other algorithm corresponds to Birch, Hierarchical Clustering and Gaussian Mixture for the top three heatmaps and to KMedoids, and Spectral Clustering for the last two
- KMeans and KMedoids results are very close except for KMedoids cluster 0 which is split in two by the KMeans algorithm,
- Spectral Clustering and Gaussian Mixture results are more different compared to KMeans. Moreover, the results of these two algorithms are not similar.

Figure 14 presents the concordance matrices for the semantic distance as illustration of the comparison of the results between algorithms [for Gower ’s distance, see Additional file 5]. In each heatmap, columns correspond to the KMeans clusters and rows to the clusters for the other algorithm. The other algorithms are Birch, Gaussian Mixture, and Hierarchical Clustering for the top three heatmaps and KMedoids and Spectral Clustering for the two bottom ones. It appears that:

- Birch and Hierarchical Clustering clusters are very close to the KMeans clusters,

We have a good concordance between three of the algorithms, the fourth one (KMedoids) has only one major difference and the last two have some clusters which are mixed up. Moreover, the concordance is similar for Gower’s distance for all algorithms except Gaussian Mixture, in particular for 6 clusters. We therefore decided to focus on the results of the KMeans algorithm with 6 clusters for the following analyses.

**Fig. 15:**
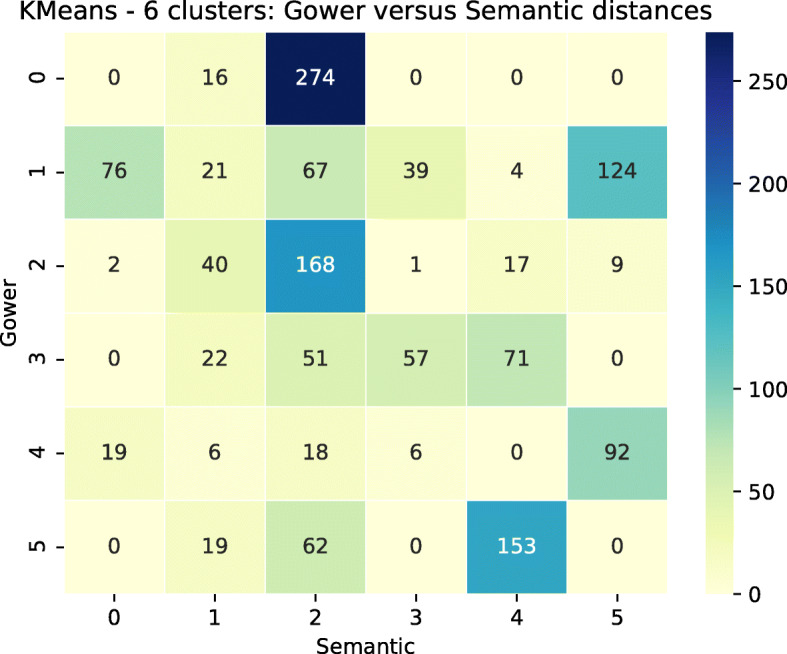
Heatmap of the concordance table between KMeans clusters for 6 clusters built with Gower’s distance (rows) and the semantic distance (columns)

The concordance matrix for the comparison between semantic and Gower’s distances is presented Fig. 15 for the KMeans algorithm and 6 clusters. No group is similar between the two distances. Most groups from Gower’s distance are spread among the various semantic groups. Group sizes are more balanced with Gower’s distance. We can again assume a link with the larger spread of points in the semantic case.

### Archetypes and visualisations

**Fig. 16:**
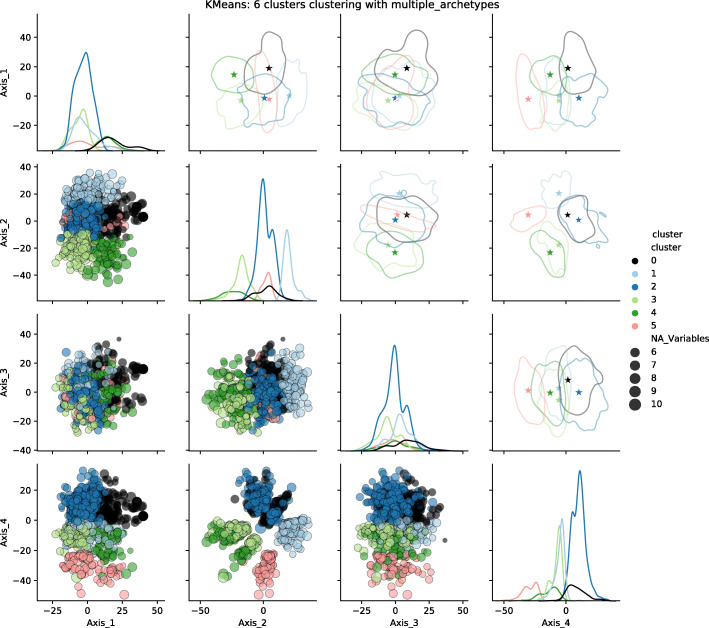
Clusters and archetype visualisations, single archetype, for the semantic distance, KMeans algorithm, 6 clusters. The *NA*_*Variables* legend illustrates some of the dot sizes representing the uncertainty: the larger the dot, the more variables are missing

**Fig. 17:**
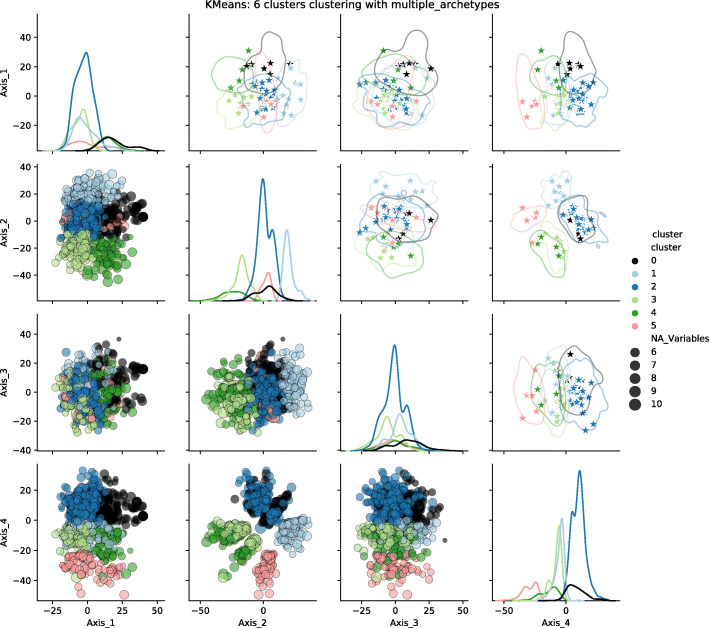
Clusters and archetype visualisations, multiple archetypes, for the semantic distance, KMeans algorithm, 6 clusters. The *NA*_*Variables* legend illustrates some of the dot sizes representing the uncertainty: the larger the dot, the more variables are missing

The next stage consisted in representing each group by either one or several individuals in order to build a less cluttered visualisation. We called these representative individuals archetypes. We calculated the archetypes positions for both distances and both approaches: one or several archetypes per group. The resulting visualisations are presented for the KMeans algorithm, 6 clusters, and the semantic distance in Fig. 16 for the single archetype approach and Fig. 17 for the multiple archetype approach. Equivalent figures for Gower’s distance are provided as Additional files 6 and 7.

The projection according to the 4 axes of the mMDS (1/2 matrix at the bottom left) shows a structuring of the 6 groups along plans 2 and 4. Axis 2 discriminates between clusters 3 and 4 on the one hand and cluster 1 on the other. Axis 4 mainly contrasts cluster 5 with clusters 0 and 2.

One can compare our representations (top right corner of the scatterplot matrix) with the whole dataset scatter plot (bottom left corner of the matrix). Our representations (top right corner of the scatterplot) provide a direct overview of the dispersion of the population and its structuring as groups compared to a classical representation (bottom left corner of the scatterplot). Regarding the number of archetypes, single or multiple archetypes per group seem relevant. The choice of one or the other is a matter of user preference and the number of clusters. The more clusters there are, the fewer archetypes per cluster are required to provide a good insight into the dataset.

### Biological interpretation of the clusters

We first of all compared the clustering for both distances at a global level. The sizes of the groups are much more variable with the semantic distance (from 90 to 649) than with Gower’s (from 141 to 331). Moreover:

- Semantic group 0 is split into the 6 Gower’s groups.
- Semantic group 1 is mainly split between Gower’s groups 3 and 5.
- Semantic group 2, with a membership of 649 individuals, includes virtually all but 17 individuals from Gower’s group 0, but also individuals from each of the other Gower’s groups, in particular group 2.
- Semantic group 3 is mainly split between Gower’s groups 1 and 4.
- Semantic group 4 is mainly split between Gower’s groups 1 and 4.
- Semantic group 5 is mainly split between Gower’s groups 1 and 3.

We went further into this comparison by looking at the underlying level, *id est* at the variables level. In order to do so, we performed *Chi*^2^ tests to evaluate whether each qualitative variable contributed to the clustering. It is the case for all variables except ’Bush height’, ’Quantity of prickles’, and ’Perfume intensity’. This is not unexpected given we have very few data corresponding to these variables. We performed a Correspondence Analysis (CA) for each of the other variables, using the *prince* Python package. We then looked more closely at the composition of the clusters.

**Fig. 18:**
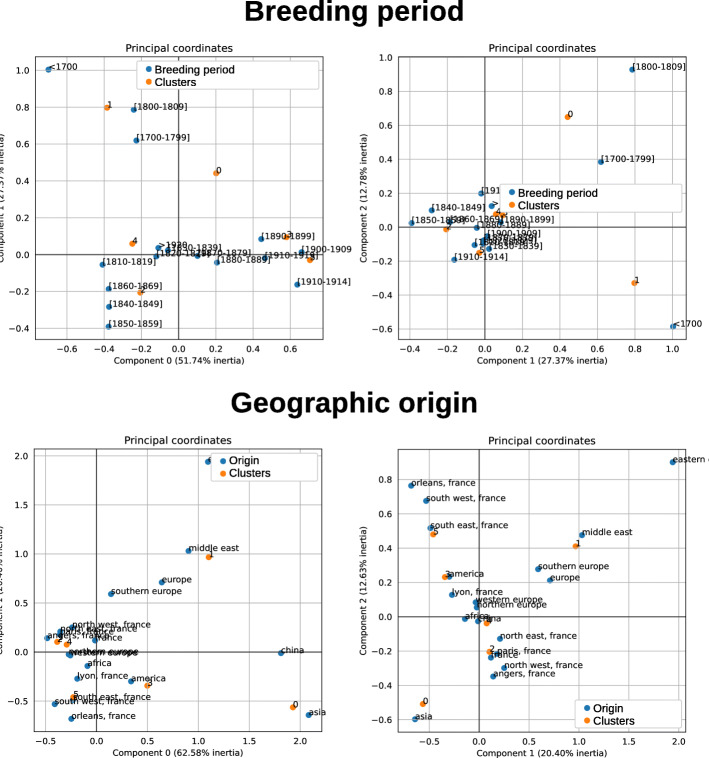
Correspondence analysis for the breeding period (top) and geographic origin (bottom) variables, for the 6 clusters obtained with the semantic distance and KMeans algorithm. The plane corresponding to components 0 and 1 are on the left and components 1 and 2 on the right

The projections reveal a stronger structuring of the modalities of the variables with the semantic distance. For instance, the geographic origin and breeding period variables reflect rose breeding history, as presented in Fig. 18. Breeding was indeed at its beginning during the 17^*th*^ century and new varieties were not very different from botanical ones, whose breeding date is before 1700. This is coherent with the projection of these modalities in the north-west part of the CA graph. Three modalities of the middle of the 19^*th*^ century (1840 to 1869) are well grouped. They correspond to the period of intense first hybridisations between Chinese and European varieties. Further periods are distributed in almost chronological order along the first axis: this may be interpreted as a continuity in the breeding schemes. Surprisingly, the “after 1920” modality is positioned near those of the first part of the 19^*th*^ century and we might wonder why.

With Gower’s distance, there is no clear structuring according to the period. All Gower’s groups have many periods that are neither over nor under-represented. The “period” variable is therefore not very discriminating, probably because the distance between the beginning and the end of the century is not greater than the distance between two consecutive decennial periods.

In all cases, the structuring with the semantic distance increases the distance between groups of modalities and reduces the intra-group spread.

We also used the *catdes* function from the *FactoMineR* R package [47] to evaluate the variable modalities that are significantly under or over-represented in each group according a *Chi*^2^ test.

With the semantic distance, cluster 0 is composed of polyploid varieties which were not or very little selected: “before 1700” and/or wild varieties. Clusters 1 and 2 regroup the first recurring flowering hybrids, but cluster 2 specifically regroups tetraploid rosebushes with pink or red flowers. Cluster 3 is mainly composed of varieties that are often diploid and yellow, obtained mainly at the end of the 19^*th*^ century: yellow tea or Noisette (the latter being obtained, at least for part of them, in America). Cluster 4 includes an over-representation of wild and diploid individuals obtained in the 18^*th*^ and early 19^*th*^ centuries, with over-represented Asian (including Chinese). Cluster 5 is mainly composed of individuals with a single duplication, mostly yellow or white, mostly obtained at the end of the 19^*th*^ century. The single duplication over-represented in this group would correspond to the introduction of previously unused wild species (which generally have single duplication flowers), bringing in a new genetic source for the yellow colour into the breeding schemes.

Using Gower’s distance, cluster 0 regroups tetraploid reflowering individuals with a red and double flower. Members of cluster 5 have the same characteristics except they have a simple flower. Cluster 1 is mainly composed of diploid, semi-double, white, yellow, or orange individuals obtained at the end of the 19^*th*^ century while cluster 3 regroups varieties often yellow, obtained mainly at the end of the 19^*th*^ century, but triploid with simple flowers. This is coherent with the more massive introduction of the yellow colour in the breeding programs at this time period. Clusters 2 and 4 regroup polyploid varieties with pink flowers, more tetraploid and of European origin obtained mainly during the first half of the 19^*th*^ century for cluster 2, and more diploid of the second part of the century for cluster 4.

All Gower’s clusters have at least one over-represented type of petal colour, while two of the semantic clusters are not distinguished by colour (clusters 0 and 1). With Gower’s distance, the red and pink petal colours are over-represented in several clusters, whereas, with the semantic distance, they are in only one cluster (cluster 2). The ’red-pink’ modality has a much more structuring effect with the semantic distance than with Gower’s, probably because its distance from the colours yellow and white is greater with the semantic distance than with Gower’s. The difference in numbers between individuals with pink or red flowers (very numerous) and individuals with yellow or white flowers (much less numerous) may play a role in this structuring. With the semantic distance, these last two colours are over-represented in two or even three clusters (clusters 3 and 5 for yellow, clusters 3, 4, and 5 for white) whereas they are mainly over-represented in Gower’s cluster 1 and a little in cluster 3. The influence of the size of the clusters on the structuring when using Gower’s or semantic distances could be further investigated in future work.

We do not have a ground truth to compare our results with since no clustering based on this dataset has been previously performed. Therefore we cannot conclude whether one distance leads to a better clustering than the other. We can only evaluate their consistency from a biological view point. Both cases lead to relevant clusters. However each distance allows to explore the dataset from a different point of view.

## Discussion

The introduction of our new semantic distance and of the archetype concept allowed us to build a comprehensive representation of an incomplete dataset characterised by a large proportion of qualitative data. Even if the current prototype is closely linked to the example dataset, it can be used as a proof of concept for a more general methodology. This can be useful from several perspectives.

Incomplete datasets including mixed (quantitative and qualitative) data are becoming more and more common in life sciences. The approach we developed allowed us to fully explore the available dataset, even though we simply ignored missing data. Indeed, as long as a pairwise distance can be calculated for all pairs of individuals, they can all be taken into account in the subsequent dimension reduction, clustering, archetype definition, and visualisation.

Regarding distances, we introduced a semantic distance as an alternative to distances tailored to mixed data such as Gower’s. This semantic distance (Eq. (3)) accounts for the underlying meaning of qualitative variables. It can be attached to distances calculated in ontologies, real-life measures such as geographical distances or associated with specific calculations such as the distances between time periods we defined in Eq. (2). It can also be based on expert knowledge regarding both the structuring of the modalities of the variable (i.e., the concepts graph of an ontology) and the distance values between two concepts. This semantic distance brings more precision regarding how two individuals relate to each other compared to Gower’s, which is more binary. The semantic distance therefore leads to a wider range of possible distance values in the dataset and as a consequence a more realistic spread of the data points in the new coordinate space.

We performed an experiment with different values for all manually defined distances (i.e. except for colours and geographical areas) to illustrate how our approach takes into account expert knowledge and the impact this has on the visualisation. In the first scenario, we defined the distance values to represent the ordered nature of the modalities of the variable without introducing a clear separation between modality groups. The corresponding distance matrix for the quantity of prickles ontology from Table 2 is presented in Table 4.

**Table 4:** Distance matrix for the quantity of prickles ontology, with no separation between groups

	Low	Medium	High	Very high
Low	0	1	2	3
Medium	1	0	1	2
High	2	1	0	1
Very high	3	2	1	0

In the second scenario, we set all distances to 1 so that all modalities are equidistant, which leads to the distance matrix from Table 5 for the quantity of prickles.

**Table 5:** Distance matrix for the quantity of prickles ontology, with equidistant concepts

	Low	Medium	High	Very high
Low	0	1	1	1
Medium	1	0	1	1
High	1	1	0	1
Very high	1	1	1	0

We compared these two scenarios with the values previously used throughout the paper, labeled as “normal”, and Gower’s distance. We can see in Additional file 9 that the dispersion of the individuals decreases from scenario (1) to scenario (4). This reduction of the dispersion of the individuals corresponds to a break-up of the groups. The reference being our “normal” ontology, we can build 3 concordance matrices [See Additional file 10], with the “normal” clusters in columns and those of the other scenario in rows. In all cases cluster 0 is not found with the other scenarios. The other clusters break up more and more in subgroups throughout the various concordance matrices, from left to right. More precisely:

- (1) vs (2) Comparison: clusters 1 to 5 are mostly kept.
- (2) vs (3) Comparison: clusters 2 to 4 are mainly split into 2 subgroups while clusters 1 and 5 remain almost unchanged.
- (3) vs (4) comparison: all the clusters found with our semantic distance are split into 2 majority subgroups.

Our distance representation allows us to better structure the scatter plot even if the distance between the different modalities in the ontology remains a delicate parameter to tune.

Relying on ad hoc distances in concept graphs for some variables, we had to capture this information in an ontology format. We did just that in OWL format: we defined a *has*_*distance* relationship. This relationship links two concepts and stores the distance value within a *distance* property attached to it. Giving a numeric value to the distance between two concepts is difficult for domain experts, but such an approach also present advantages. These distances are data and they can be easily changed, which once again brings flexibility to the way we process the datasets.

Moreover, our semantic distance is defined as a weighted sum. Therefore it gives more or less importance to some variables compared to others, thus granting the ability to fine tune the way each facet of the dataset is managed.

Finally, we tackled the problem of cluttered scatter plots by reducing the number of displayed individuals.

From the application point of view, we illustrated our approach with passport and phenotypic data of rose varieties. But it could be used for any dataset describing a large set of organisms, for instance in ecology, and including other types of data, for instance genomic. More widely, it could be used for any incomplete dataset mixing qualitative and quantitative variables. A problem that presents similar premises (reduce the number of individuals representing a population) is the constitution of core-collections by BRCs. Indeed BRCs store large collections of biological material and associated information, and they often need to constitute sub-samples of a more manageable size e.g. for experimental purposes. These core-collections include, with a minimum of repeatability, the maximum diversity of the species in question [48] and are designed by exploiting the maximum amount of data available: the origin of the samples, genetic and phenotypic characteristics, etc. The existing strategies for the selection of inputs are diverse: random sampling, partitioning (also called “stratification”), maximisation, and some other so-called “hybrid” strategies [49]. The methodology presented here could add a new tool to the arsenal of BRCs.

However, even if it is functional, the methodology presents some limitations.

First of all the method relies on ontologies. Reference characterisation of individuals in the plant sciences domain is becoming more common. Examples include the MIAPPE (Minimum Information About a Plant Phenotyping Experiment) [50] minimum requirements or the ontologies of the Planteome (https://planteome.org) databank [51], in particular the Plant Trait Ontology. Sharing more reference ontologies would spread the knowledge engineering effort further. Moreover, some distances have to be associated with these ontologies. Some ontologies may be linked with measures, such as time periods, colours, and geographic locations, but it is not always the case. Relying on distances calculations based on the graph topology or information content scales well for all ontology graphs but doesn’t necessarily represent reality. For some rosebushes phenotypic traits, we chose to define the distances between concepts in the ontologies with expert knowledge for better representation. It was feasible because our graphs were small. We then had to translate relative distance information expressed by experts into numeric values. This has to be carefully managed in order to avoid introducing some bias. Methods to better anchor the distances with quantifiable information have therefore to be designed. Moreover, the more closely the distance reflects biological reality, the better the results. For example, when considering petal colour, instead of using the DBpedia colour ontology, it could be better to estimate a distance between two biosynthesis pathways leading to the yellow and red colours respectively (e.g. by counting the number of different enzymes in the pathways). Scientific knowledge supporting such an exhaustive approach is still limited but with the semantic distance such new knowledge can be integrated over time and new analyses performed.

Secondly, the management of missing data could be further refined. The distance between individuals defined in Eq. (3) calculates a distance with any individual having missing data. However, the pairwise distance can be calculated based on different numbers of variables, depending on the number of variables where two individuals share values. For instance, in our rosebush example, we have distances calculated from 1 to 9 variables out of 11 potential variables for individuals with a complete record. In this context, we might want to consider that a distance calculated based on more variables is more accurate than one calculated with less. As a readily available proxy to represent this accuracy (or lack thereof), we encoded the number of missing data for each rosebush as the size of the dot representing the rosebush in the visualisation. A better approach to represent this accuracy might be to encode the pairwise distance not as a number but as an interval or a fuzzy number. Another approach would be to associate an error with the distance. We then would have to perform the next stages of the process (dimension reduction, clustering, archetype definition, and visualisation) based either on fuzzy data or error prone data. Methods are described in the literature, for instance for fuzzy MDS [52] or fuzzy clustering [53]. We, however, have to study the topic more thoroughly and find implementations of the described approaches or develop our own.

Thirdly the approaches we used to build the archetypes representing the clusters may not be the most relevant. We might want to better associate the construction of these individuals with the values of the variables in the original dataset. A better archetype might indeed be an “artificial” one whose variable values are the most represented in its cluster. Defining an archetype this way however introduces new problems. It would have to be projected in the new coordinate space created by the dimension reduction so that it could be represented in the visualisations. It isn’t a trivial task given we can only calculate distances between individuals. The topic would have to be explored further.

Fourthly the visualisations we produced are static scatterplot matrices. A big improvement would be to render them dynamically and make the visualisation interactive. A graphic interface to choose which display to render (which distance, which dimension reduction method, which clustering algorithm, how many clusters, etc.) and fill in the current configuration file would be most welcome. We could imagine allowing to rotate and zoom in and out of the display. Tooltips associated with the archetypes could provide information regarding the cluster they represent, such as the number of individuals, main characteristics regarding the original variables, etc. Clicking on an archetype could change the display and lead to a visualisation of the individuals composing the corresponding cluster. The pipeline presented here was developed as a proof of concept regarding the interest of the semantic distance and archetype notions. Dynamic visualisations would regard future work.

## Conclusion

In this paper, we presented a new method to analyse heterogeneous datasets. This approach relies on a new semantic distance which is designed for both quantitative and qualitative variables. This distance can be considered as an alternative to distances designed for mixed data such as Gower’s. We associated this new distance definition with an archetype concept to overcome the cluttered displays issue. Indeed we defined archetypes as individuals representing groups of similar individuals from the dataset. Limiting the visualisations to these archetypes led to a sparser representation which still provided valuable insight into the data. For easier handling by biologists, we have incorporated their use into a complete tool, from raw data file to visualisation, implemented in Python 3.7. Following the distance calculation, the next steps performed by the tool consisted in (i) grouping similar individuals, (ii) representing each group by emblematic individuals we call archetypes and (iii) building sparse visualisations based on these archetypes. The semantic distance allows for a more realistic representation of the relationships between individuals and a wider spread of the data points. It can be linked to real-life knowledge regarding the modalities of the underlying variable or to distance measures captured in ontologies. In this respect, we defined how to describe the distance value between two concepts in OWL format.

The methodology described here was applied to a dataset describing rosebush passport and phenotypic traits but it could have wider uses. Indeed we could apply the same approach to any mixed dataset. Moreover, the selection of a representative subset of a population is a widespread problem. It is a concern for Biological Resources Centres willing to build core collections for the species they are conserving. Our technique could provide a complementary methodology to existing ones.

The method is fully functional. However, some aspects imply future work. The method relies on ontologies which may have to be constructed. However, in the near future, the expansion of FAIR data principles should lead to sharing more reference ontologies and reduce the knowledge engineering work. Taking into account missing data and some kind of confidence in the pairwise distance between individuals based on the number of variables used to calculate this distance has to be studied further. Future work also implies implementing an interactive visualisation to improve the data mining by biologists.

## Supplementary Information

**Additional file 1** Laplacian eigenmaps dimension reduction. visualisation with multiple archetypes and semantic distance. Clusters and archetype visualisations, multiple archetypes, for semantic distance, KMeans algorithm, 14 clusters (number chosen through Silhouette analysis).

**Additional file 2** PCoA dimension reduction. visualisation with multiple archetypes and semantic distance. Clusters and archetype visualisations, multiple archetypes, for semantic distance, KMeans algorithm, 6 clusters (number chosen through Silhouette analysis).

**Additional file 3** Silhouette analysis for the semantic distance. Silhouette values at the individual level for the KMeans algorithm, 2 to 19 clusters and the semantic distance.

**Additional file 4** Silhouette analysis for gower’s distance. Silhouette values at the individual level for the KMeans algorithm, 2 to 19 clusters and Gower’s distance.

**Additional file 5** Concordance between algorithms, gower’s distance. Heatmaps of the concordance tables between KMeans clusters for 6 clusters (columns) and the other tested clustering algorithms (rows), Gower’s distance. In each heatmap columns correspond to the KMeans clusters and rows to the clusters for the other algorithm. This other algorithm correspond to Birch, HCA (Hierarchical clustering) and Gaussian Mixture for the top three heatmaps and to KMedoids and Spectral Clustering for the two bottom ones.

**Additional file 6** Visualisation with single archetype and gower’s distance. Clusters and archetype visualisations, single archetype, for Gower’s distance, KMeans algorithm, 6 clusters.

**Additional file 7** Visualisation with multiple archetype and gower’s distance. Clusters and archetype visualisations, multiple archetypes, for Gower’s distance, KMeans algorithm, 6 clusters.

**Additional file 8** Results from the catdes r function from the factoMiner package. Results from the catdes function, used to characterise the clusters with the modalities of the original variables in the datasets, clusters calculated with the KMeans algorithm, in the 6 clusters case, for both distances (semantic and Gower’s).

**Additional file 9** Comparison of the results with different distances between concepts in the rose ontology. Comparison of clusters and archetype visualisations, multiple archetypes, KMeans algorithm according to the way the distance between concepts are considered: (1) Rose ontology as used throughout the paper and qualified as “Normal”. Distances for colours and geographic locations remains calculated as described in the paper, (2) Rose ontology with an inter-groups distance which isn’t larger than the intra-group distance. Distances for colours and geographic locations remains calculated as described in the paper, (3) Rose ontology where all pairwise distances between leaf concepts are the same. Distances for colours and geographic locations remains calculated as described in the paper, (4) Use of Gower’s distance instead of our semantic distance.

**Additional file 10** Heatmaps of the concordance tables with different distances between concepts in the rose ontology. Concordance between the semantic clusters, KMeans algorithms (6 clusters) and the different conditions detailed in the experiment from Additional file 8.

## Data Availability

The software developed to implement the pipeline presented in this paper is available as follows: • Project name: DIVIS • Project home page: https://forgemia.inra.fr/irhs-bioinfo/Divis • Archived version: v1.2 • Operating system(s): Platform independent • Programming language: Python 3.7 • Other requirements: Described as requirement.txt file for *pip* in the code repository • License: CeCILL. See LICENCE file in the code repository • Any restrictions to use by non-academics: None The OWL ontology (in French) is bundled with the code. The datasets used and analysed during the current study are available from the corresponding author on reasonable request.

## Abbreviations

BRC: Biological Ressource Centre
CA: Correspondence Analysis
DNA: Deoxyribo Nucleic Acid
IC: Information Content
PCoA: Principal Coordinates Analysis
MCA: Multiple Correspondence Analysis
MDS: Multi-Dimensional Scaling
mMDS: Metric Multi-Dimensional Scaling
OWL: Web Ontology Language
SPARQL: SPARQL Protocol and RDF Query Language
