# Supplementary figures and images for: DIVIS: a semantic DIstance to improve the VISualisation of heterogeneous phenotypic datasets
Source: BioData Min. 2022 Apr 4;15:10. doi: 10.1186/s13040-022-00293-y (PMC8981856; doi:10.1186/s13040-022-00293-y)

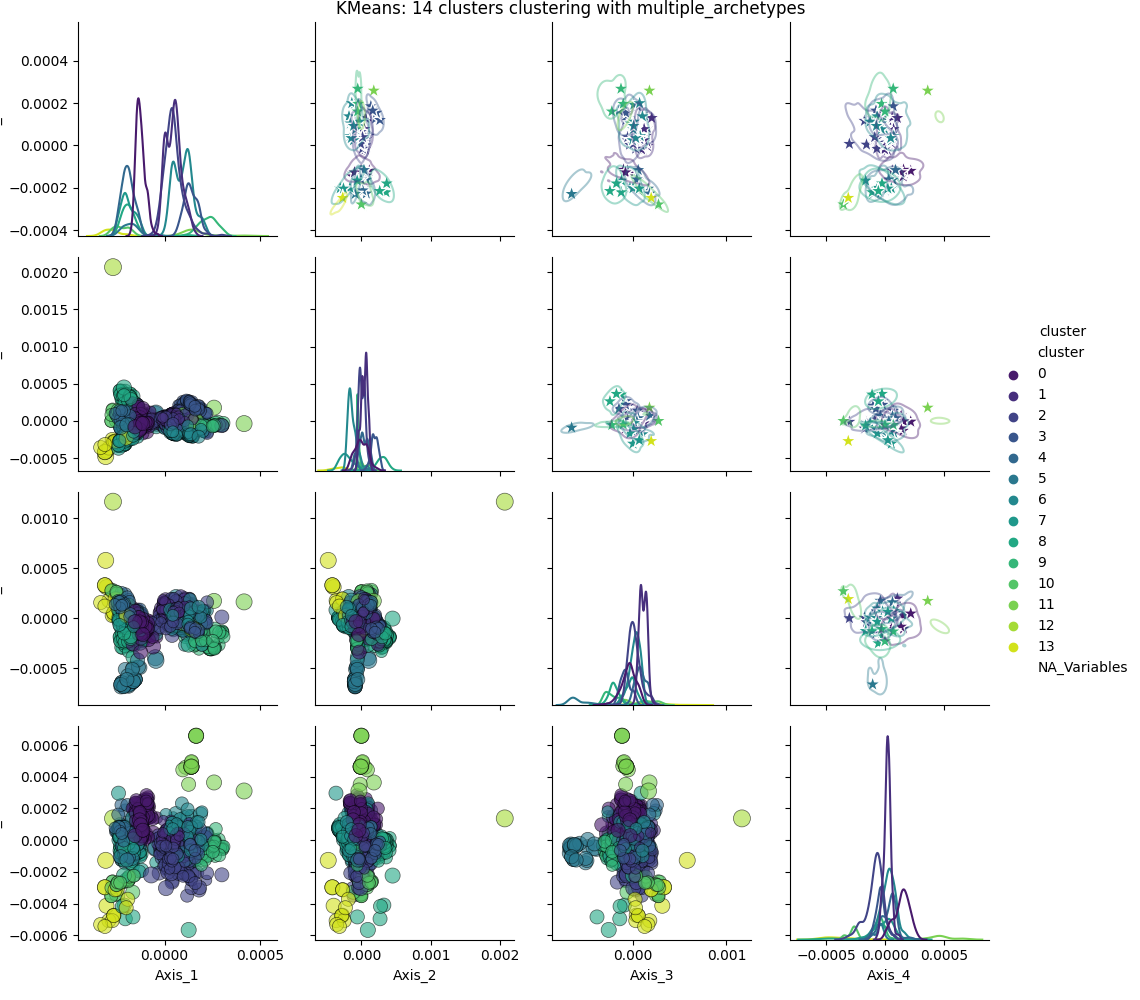

Supplement: Supplementary file 1 — Additional file 1 Laplacian eigenmaps dimension reduction. visualisation with multiple archetypes and semantic distance. Clusters and archetype visualisations, multiple archetypes, for semantic distance, KMeans algorithm, 14 clusters (number chosen through Silhouette analysis). [file 13040_2022_293_MOESM1_ESM.png]

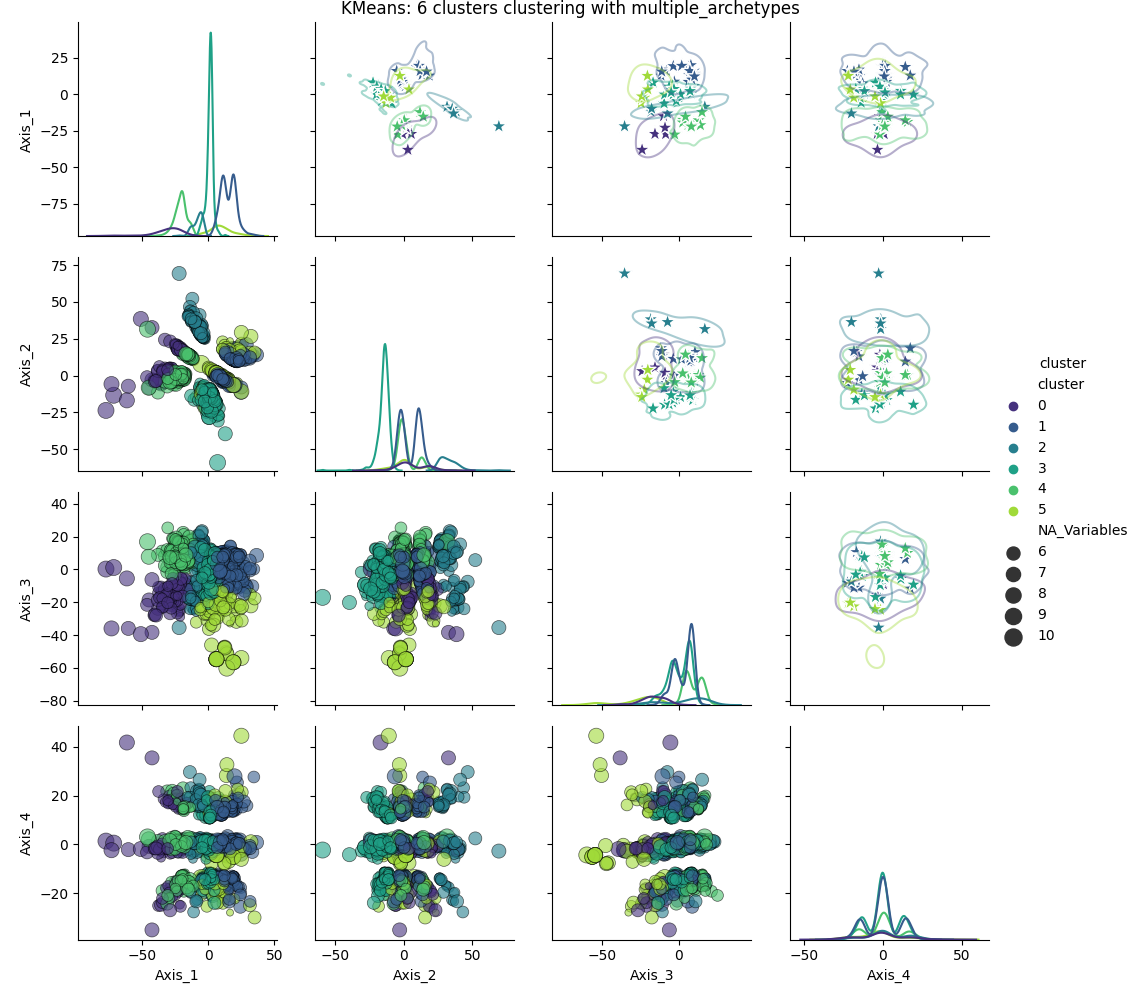

Supplement: Supplementary file 2 — Additional file 2 PCoA dimension reduction. visualisation with multiple archetypes and semantic distance. Clusters and archetype visualisations, multiple archetypes, for semantic distance, KMeans algorithm, 6 clusters (number chosen through Silhouette analysis). [file 13040_2022_293_MOESM2_ESM.png]

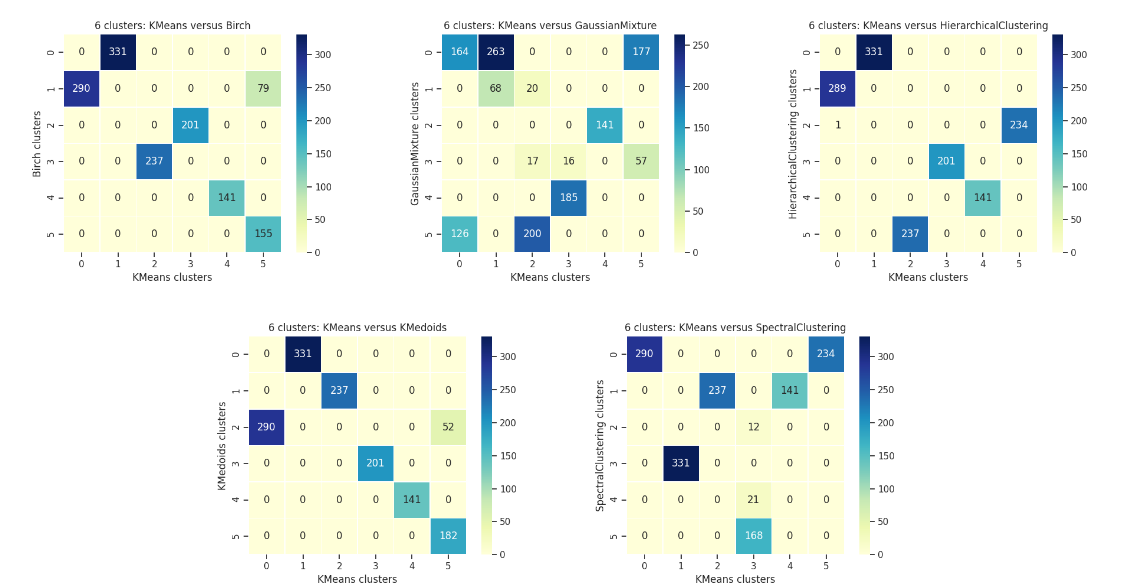

Supplement: Supplementary file 5 — Additional file 5 Concordance between algorithms, gower’s distance. Heatmaps of the concordance tables between KMeans clusters for 6 clusters (columns) and the other tested clustering algorithms (rows), Gower’s distance. In each heatmap columns correspond to the KMeans clusters and rows to the clusters for the other algorithm. This other algorithm correspond to Birch, HCA (Hierarchical clustering) and Gaussian Mixture for the top three heatmaps and to KMedoids and Spectral Clustering for the two bottom ones. [file 13040_2022_293_MOESM5_ESM.png]

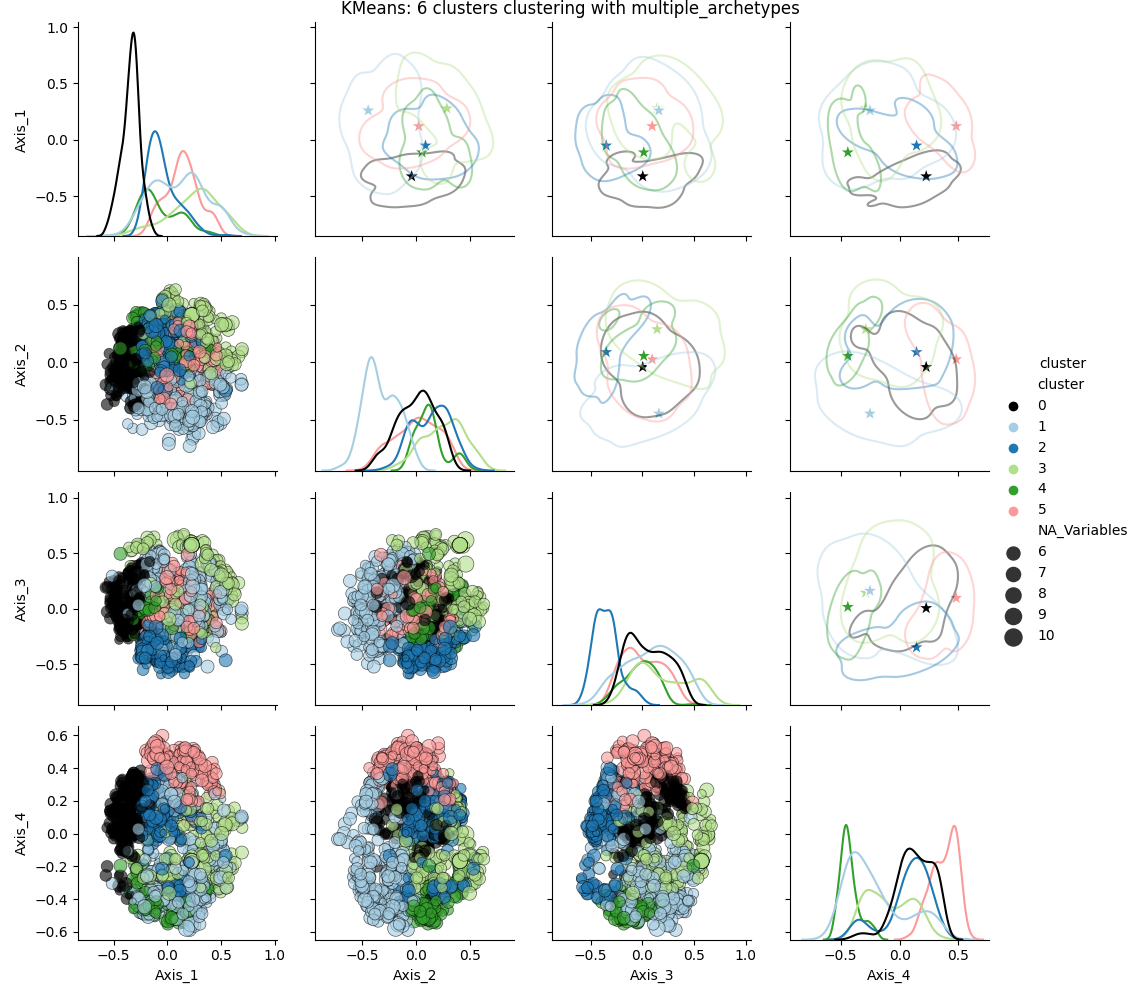

Supplement: Supplementary file 6 — Additional file 6 Visualisation with single archetype and gower’s distance. Clusters and archetype visualisations, single archetype, for Gower’s distance, KMeans algorithm, 6 clusters. [file 13040_2022_293_MOESM6_ESM.png]

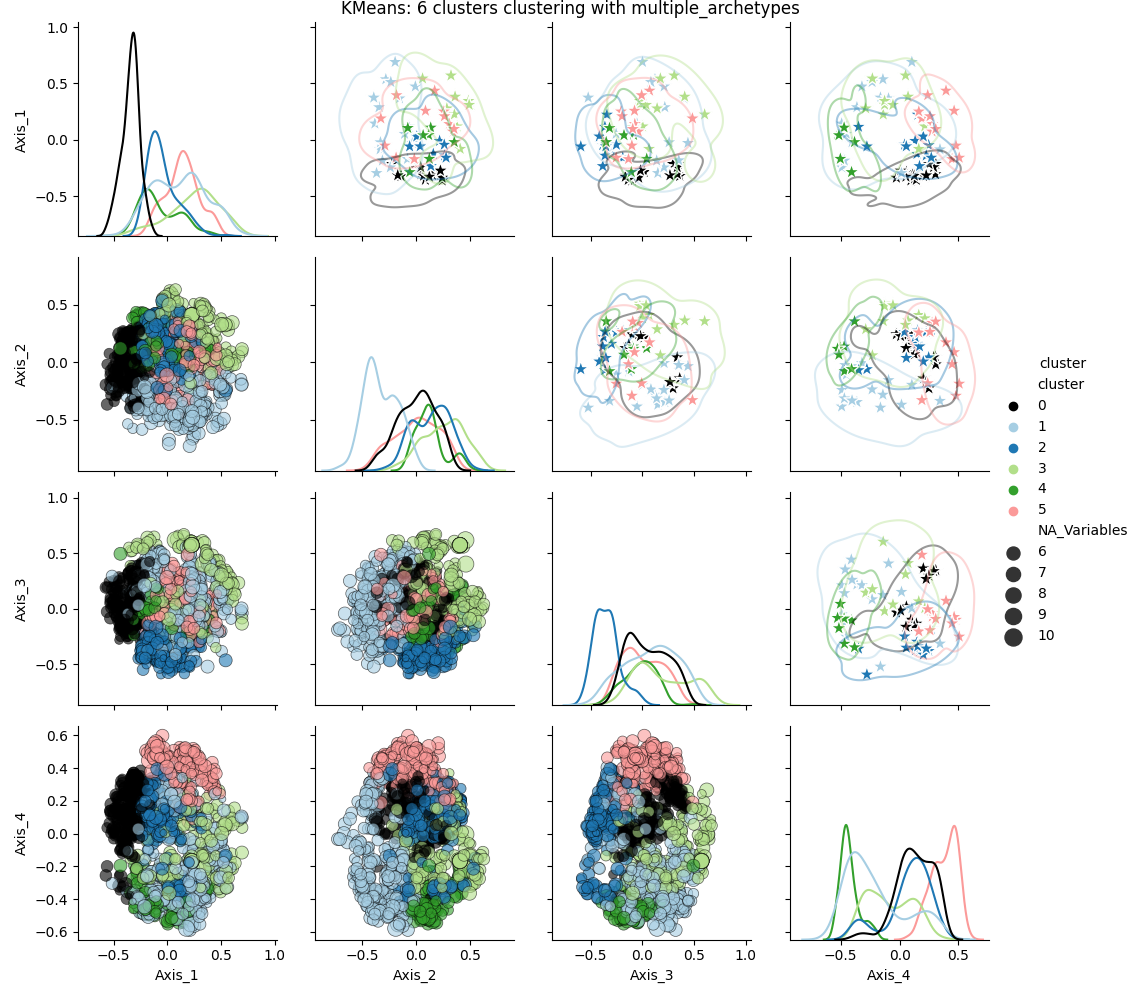

Supplement: Supplementary file 7 — Additional file 7 Visualisation with multiple archetype and gower’s distance. Clusters and archetype visualisations, multiple archetypes, for Gower’s distance, KMeans algorithm, 6 clusters. [file 13040_2022_293_MOESM7_ESM.png]

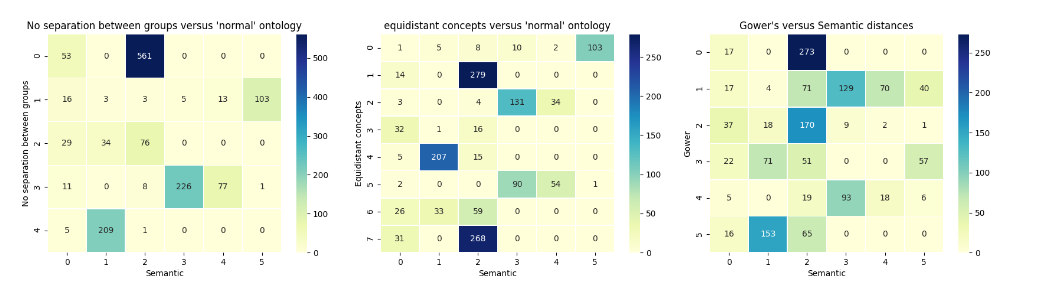

Supplement: Supplementary file 9 — Additional file 9 Comparison of the results with different distances between concepts in the rose ontology. Comparison of clusters and archetype visualisations, multiple archetypes, KMeans algorithm according to the way the distance between concepts are considered: (1) Rose ontology as used throughout the paper and qualified as “Normal”. Distances for colours and geographic locations remains calculated as described in the paper, (2) Rose ontology with an inter-groups distance which isn’t larger than the intra-group distance. Distances for colours and geographic locations remains calculated as described in the paper, (3) Rose ontology where all pairwise distances between leaf concepts are the same. Distances for colours and geographic locations remains calculated as described in the paper, (4) Use of Gower’s distance instead of our semantic distance. [file 13040_2022_293_MOESM9_ESM.png]

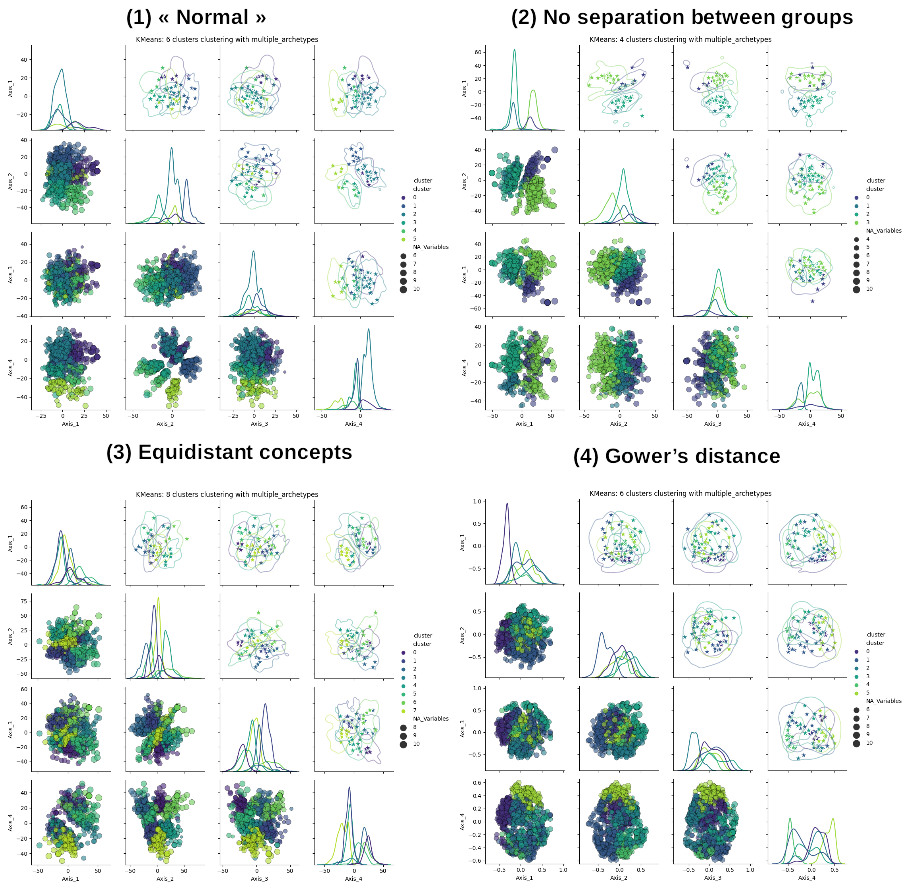

Supplement: Supplementary file 10 — Additional file 10 Heatmaps of the concordance tables with different distances between concepts in the rose ontology. Concordance between the semantic clusters, KMeans algorithms (6 clusters) and the different conditions detailed in the experiment from Additional file 8. [file 13040_2022_293_MOESM10_ESM.png]
