# Supplementary material for: DIVIS: a semantic DIstance to improve the VISualisation of heterogeneous phenotypic datasets
Source: BioData Min. 2022 Apr 4;15:10. doi: 10.1186/s13040-022-00293-y (PMC8981856; doi:10.1186/s13040-022-00293-y)

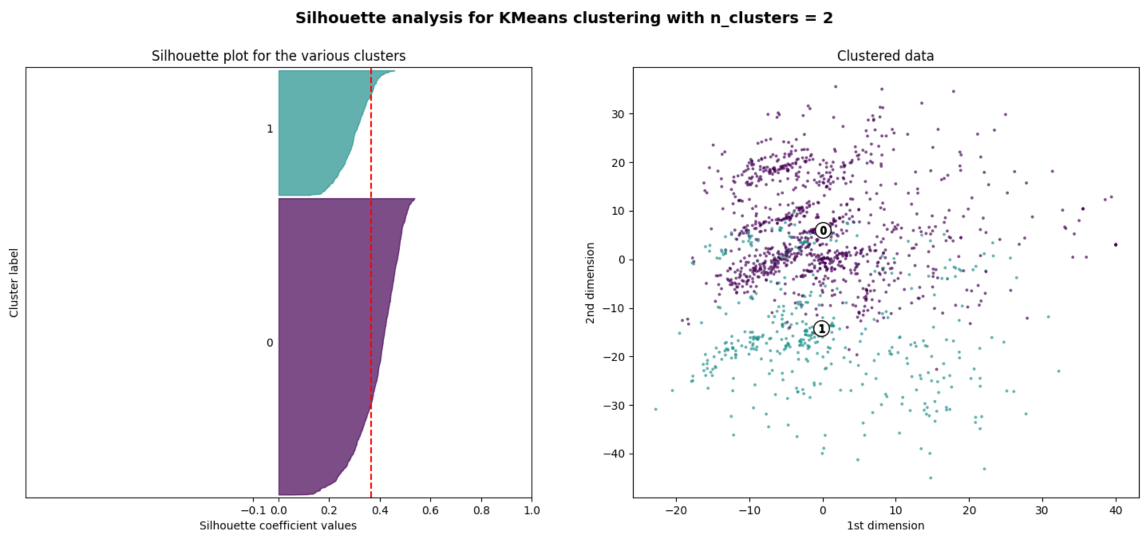
Silhouette analysis for KMeans clustering with n_clusters = 2
Silhouette plot for the various clusters
Cluster label
Silhouette coefficient values
Clustered data
2nd dimension
1st dimension

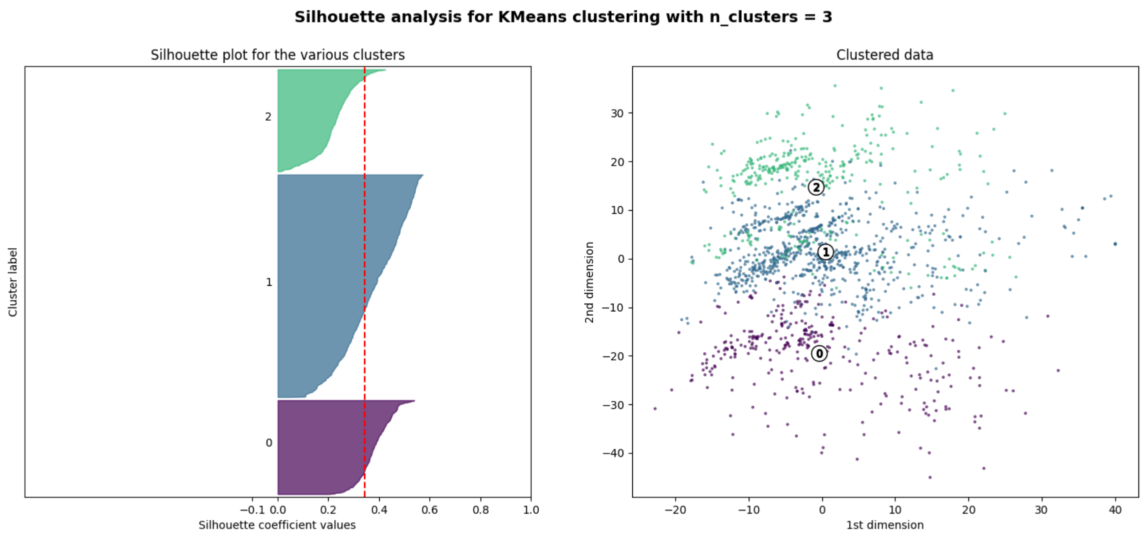
Silhouette analysis for KMeans clustering with n_clusters = 3
Silhouette plot for the various clusters
Cluster label
Silhouette coefficient values
Clustered data
2nd dimension
1st dimension

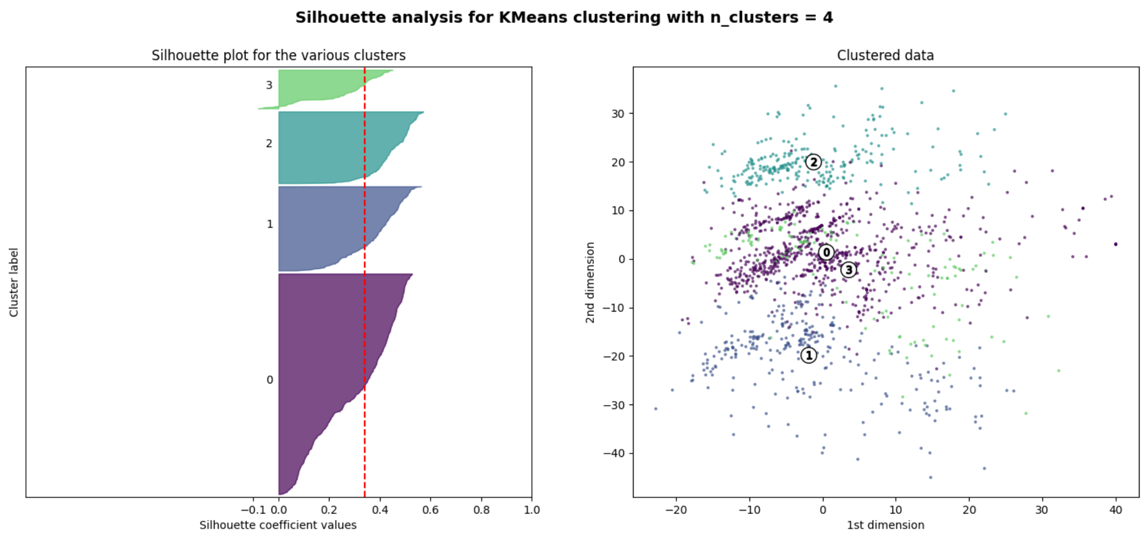
Silhouette analysis for KMeans clustering with n_clusters = 4
Silhouette plot for the various clusters
Cluster label
Silhouette coefficient values
Clustered data
2nd dimension
1st dimension

Silhouette analysis for KMeans clustering with n_clusters = 5

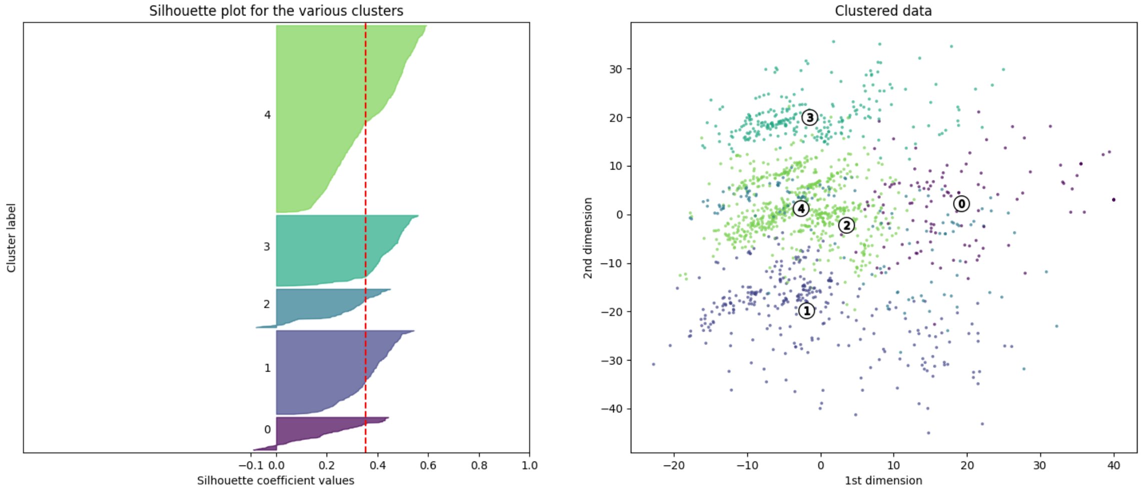

Silhouette analysis for KMeans clustering with n_clusters = 6

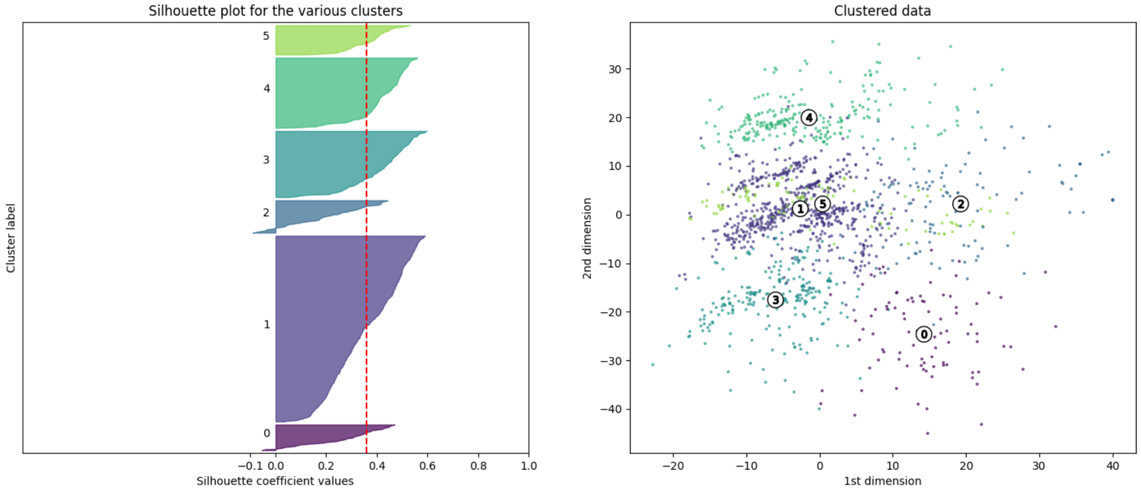

Silhouette analysis for KMeans clustering with n_clusters = 7

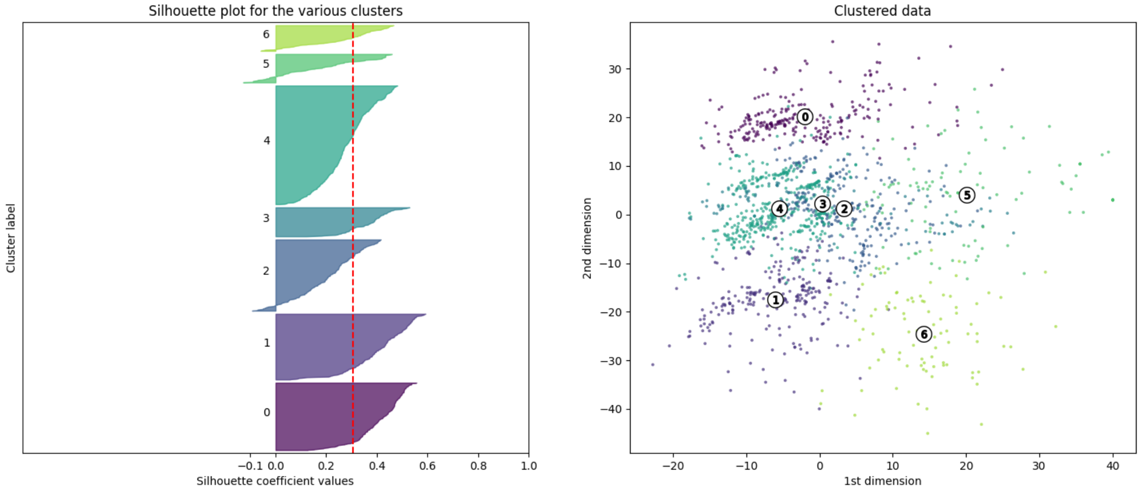

Silhouette analysis for KMeans clustering with n_clusters = 8

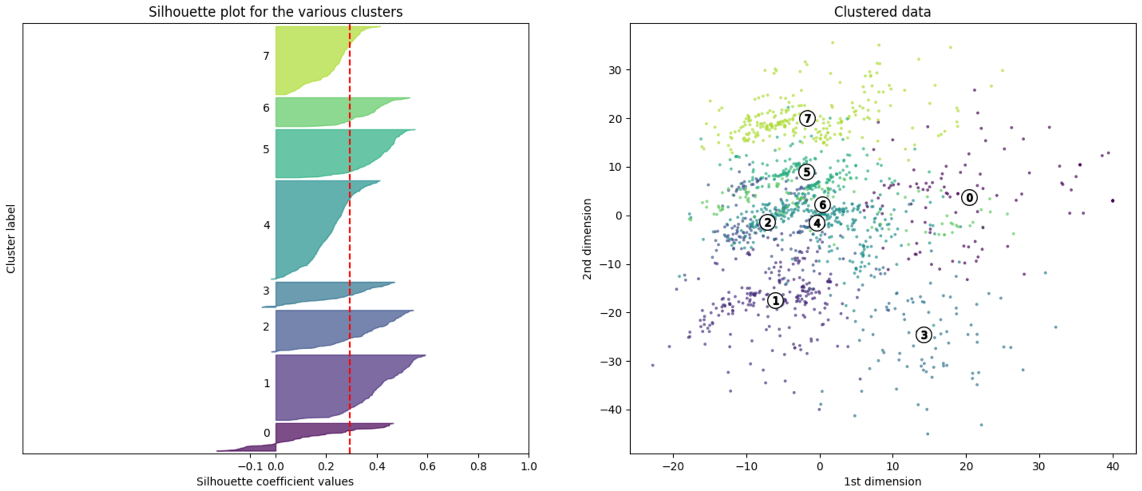

Silhouette analysis for KMeans clustering with n_clusters = 9

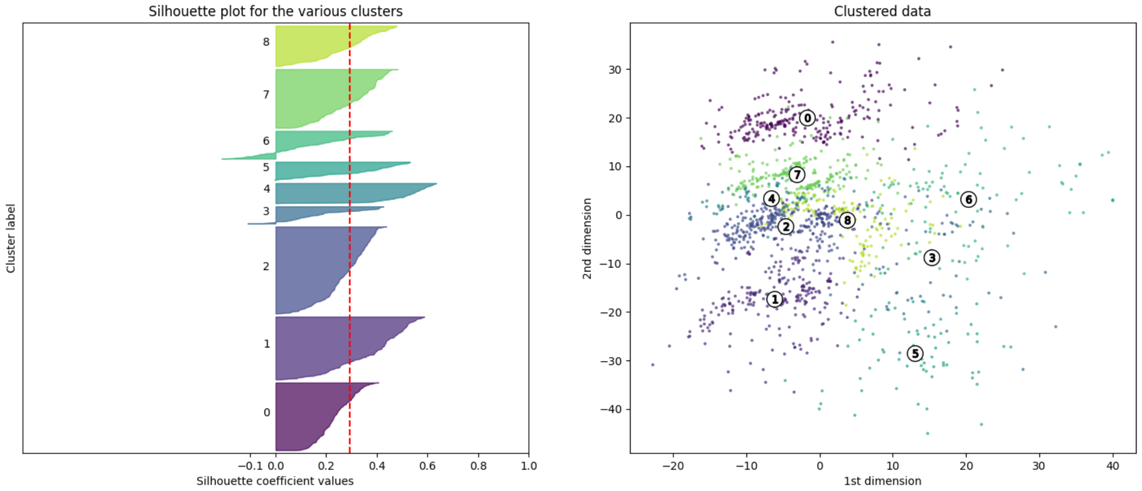

Silhouette analysis for KMeans clustering with n_clusters = 10

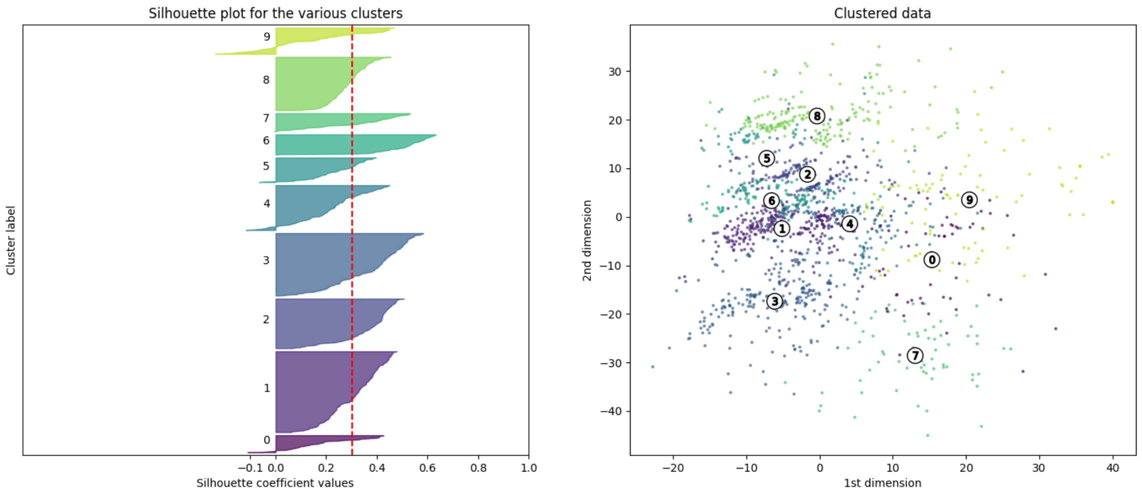

Silhouette analysis for KMeans clustering with n_clusters = 11

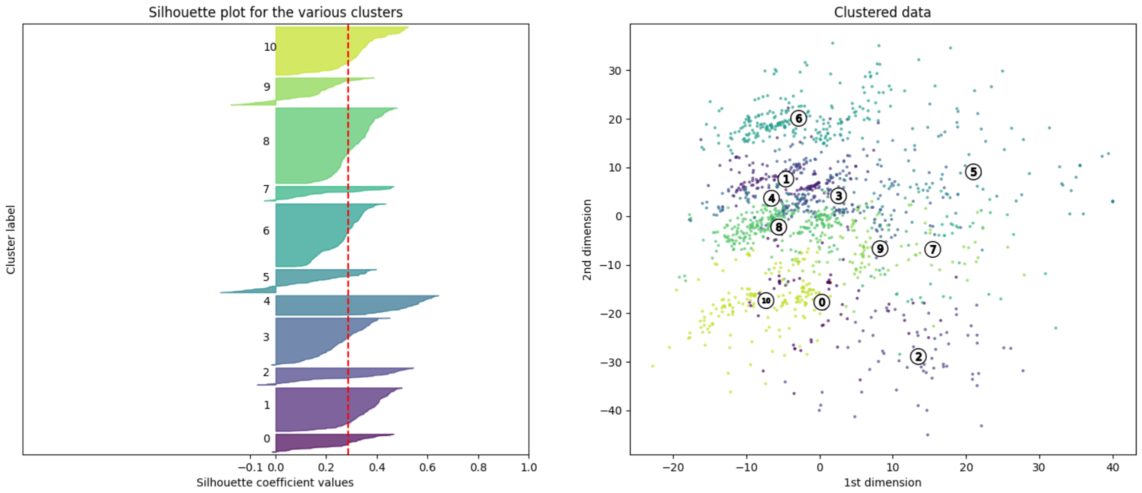

Silhouette analysis for KMeans clustering with n_clusters = 12

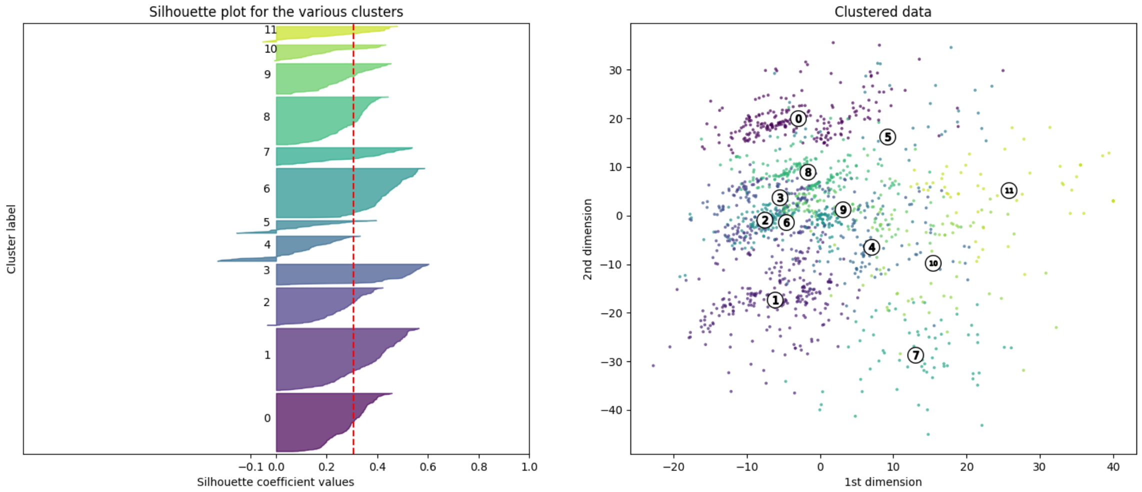

Silhouette analysis for KMeans clustering with n_clusters = 13

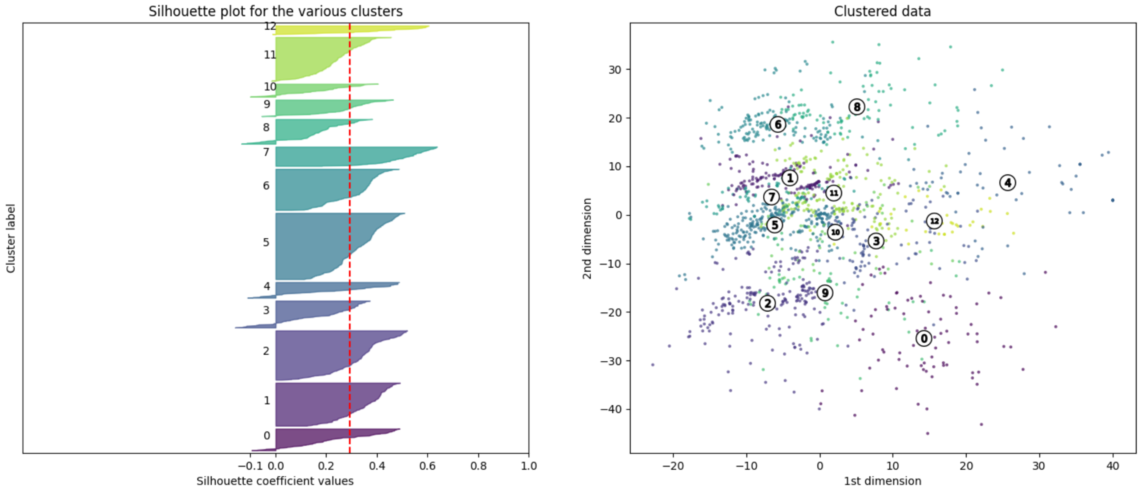

Silhouette analysis for KMeans clustering with n_clusters = 14

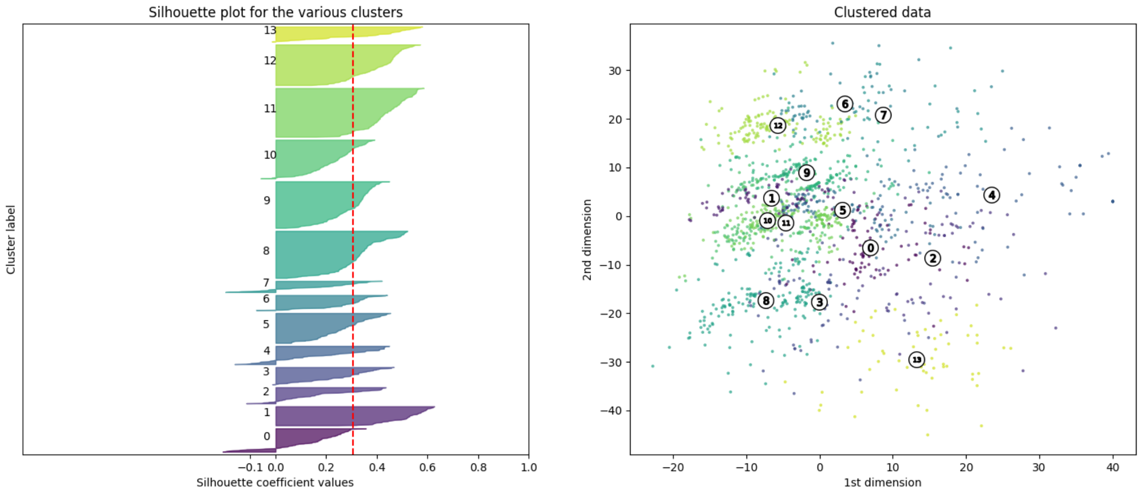

Silhouette analysis for KMeans clustering with n_clusters = 15

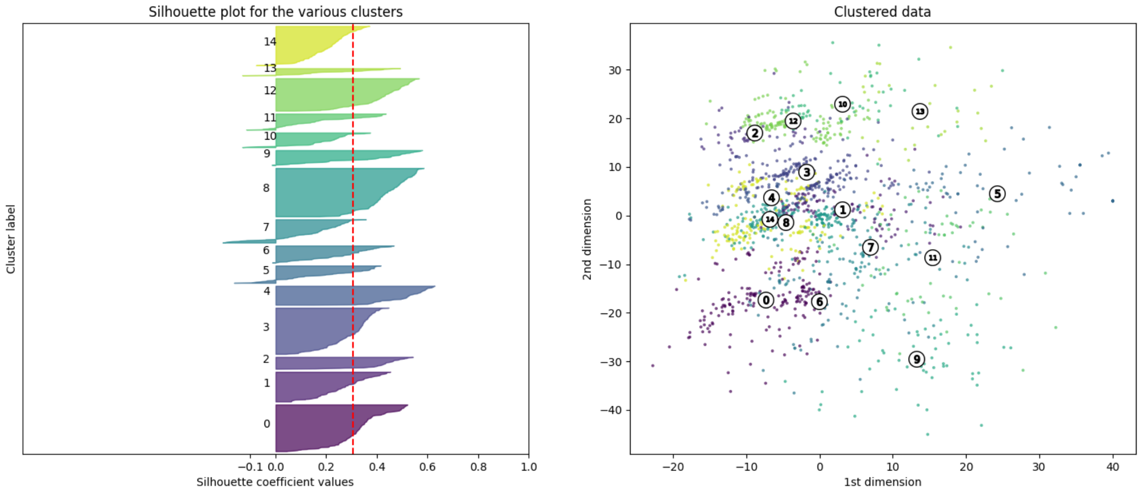

Silhouette analysis for KMeans clustering with n_clusters = 16

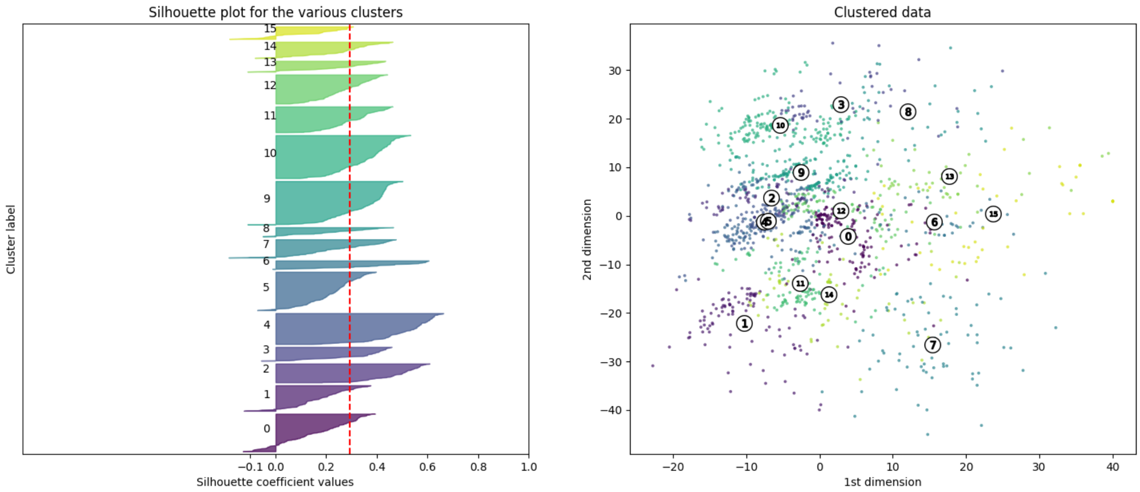

Silhouette analysis for KMeans clustering with n_clusters = 17

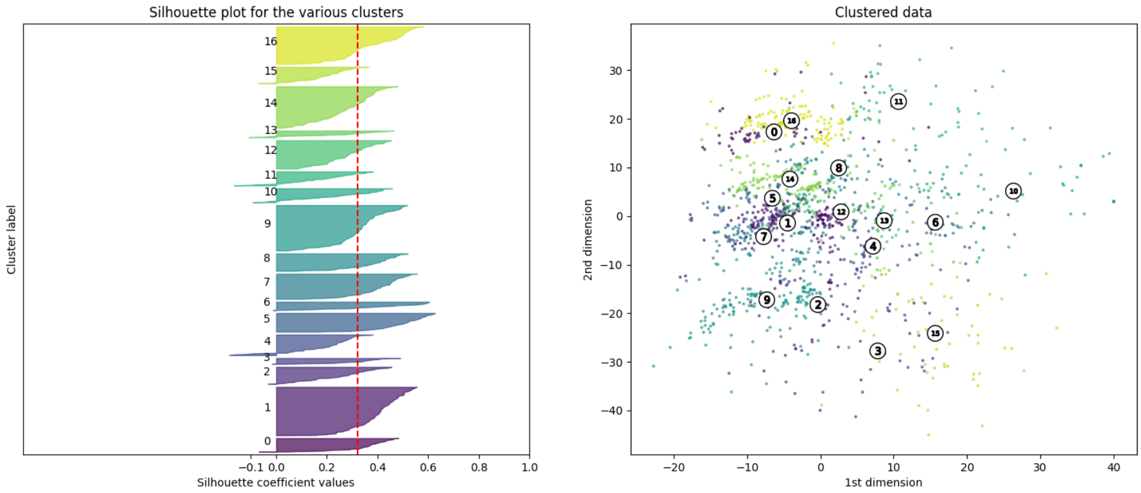

Silhouette analysis for KMeans clustering with n_clusters = 18

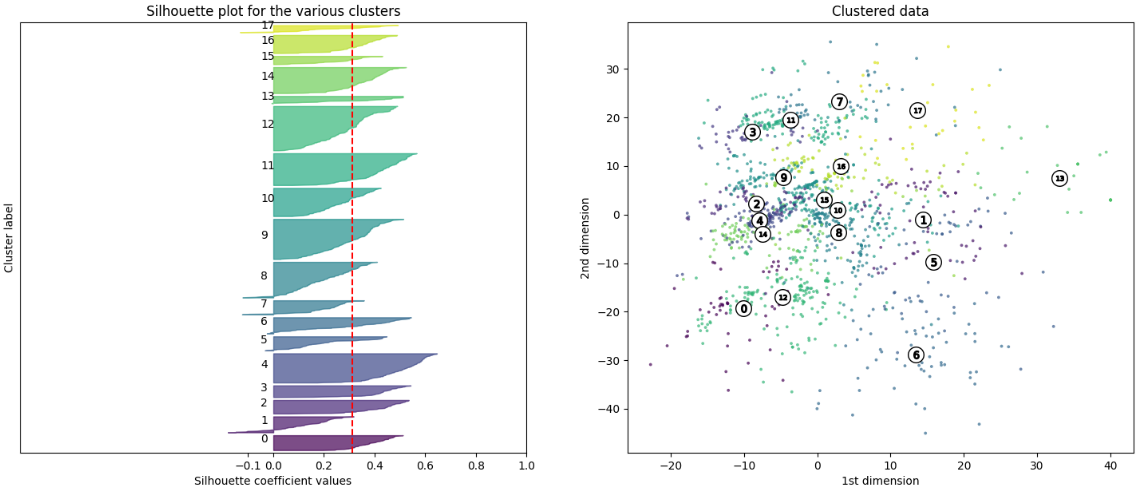

Silhouette analysis for KMeans clustering with n_clusters = 19

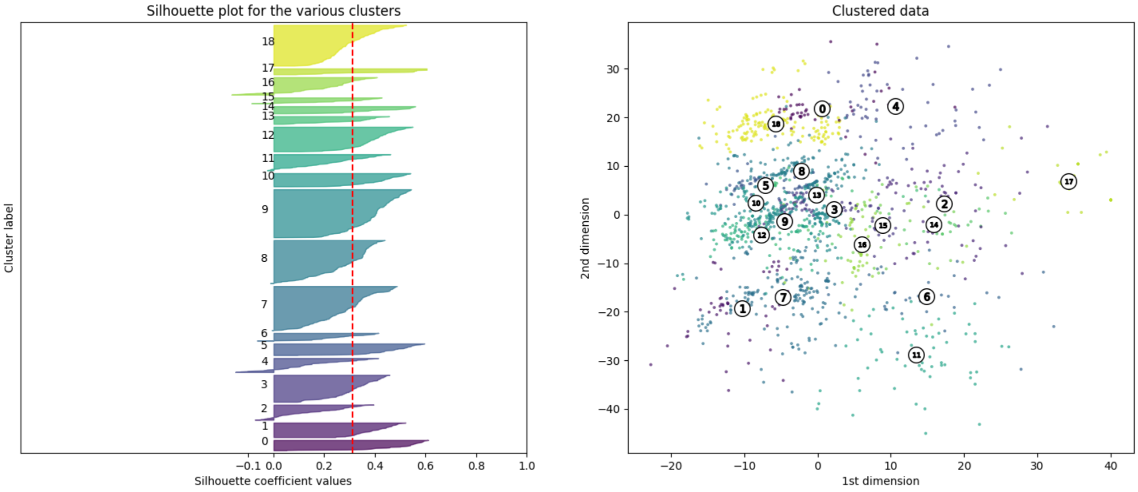

Supplement: Supplementary file 3 — Additional file 3 Silhouette analysis for the semantic distance. Silhouette values at the individual level for the KMeans algorithm, 2 to 19 clusters and the semantic distance. [file 13040_2022_293_MOESM3_ESM.pdf]
