# Supplementary material for: DIVIS: a semantic DIstance to improve the VISualisation of heterogeneous phenotypic datasets
Source: BioData Min. 2022 Apr 4;15:10. doi: 10.1186/s13040-022-00293-y (PMC8981856; doi:10.1186/s13040-022-00293-y)
Supplement: Supplementary file 4 — Additional file 4 Silhouette analysis for gower’s distance. Silhouette values at the individual level for the KMeans algorithm, 2 to 19 clusters and Gower’s distance. [file 13040_2022_293_MOESM4_ESM.pdf]

Silhouette analysis for KMeans clustering with n_clusters = 2

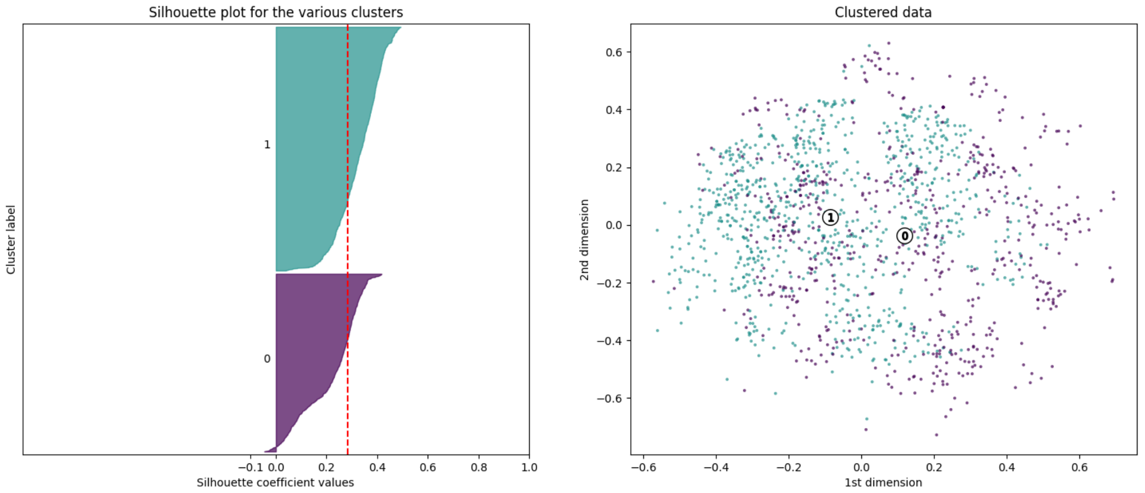

Silhouette analysis for KMeans clustering with n_clusters = 3

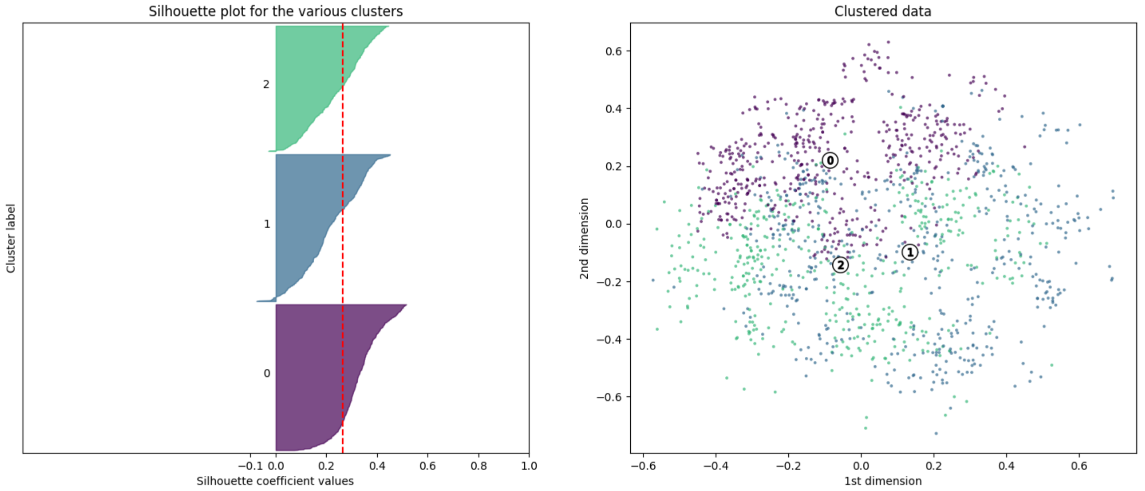

Silhouette analysis for KMeans clustering with n_clusters = 4

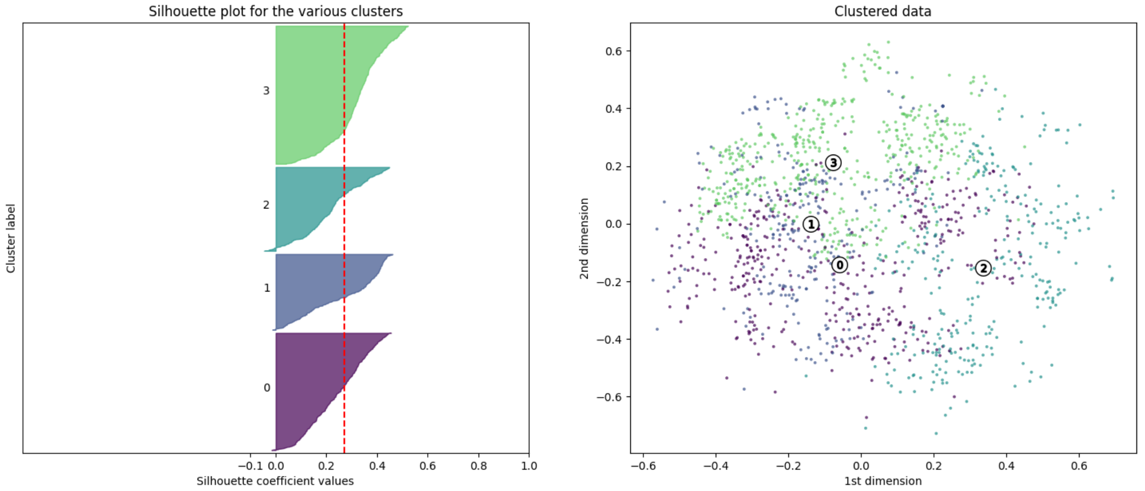

Silhouette analysis for KMeans clustering with n_clusters = 5

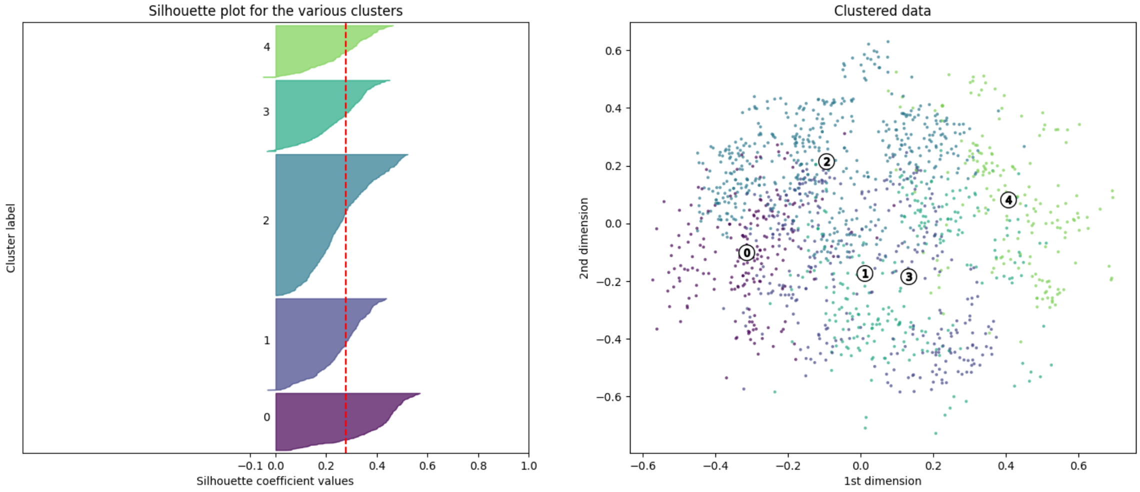

Silhouette analysis for KMeans clustering with n_clusters = 6

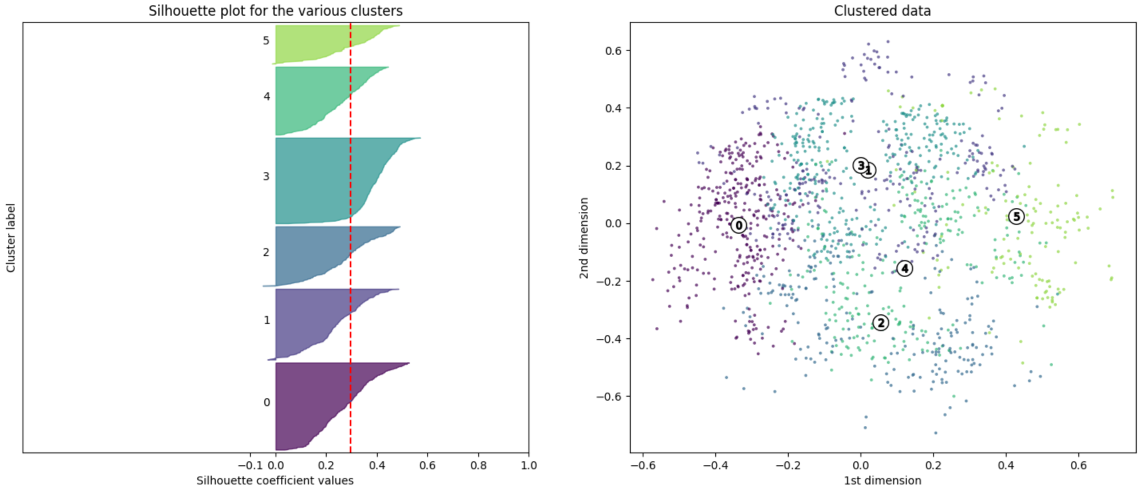

Silhouette analysis for KMeans clustering with n_clusters = 7

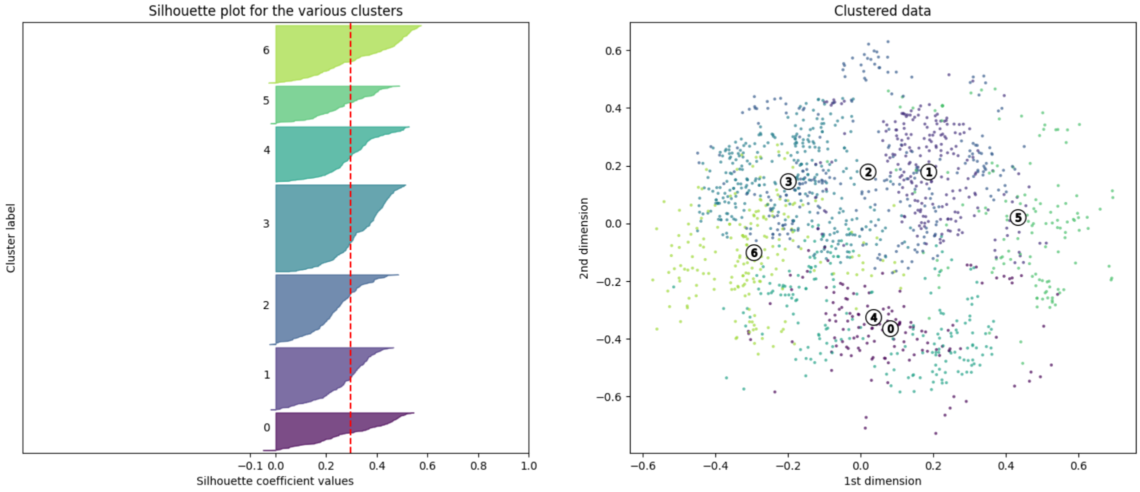

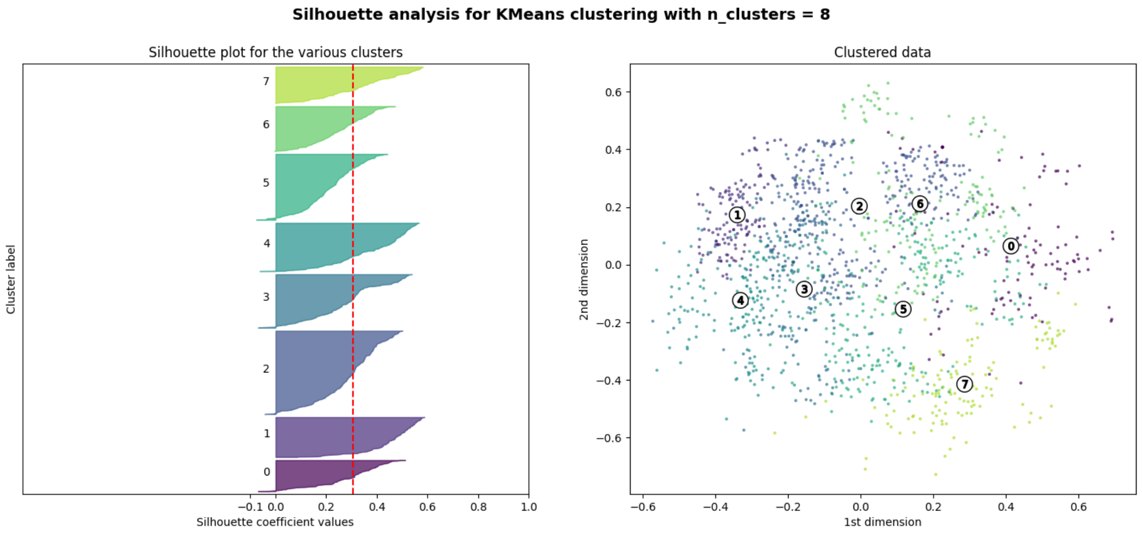
Silhouette analysis for KMeans clustering with n_clusters = 8
Silhouette plot for the various clusters
Cluster label
Silhouette coefficient values
Clustered data
1st dimension
2nd dimension

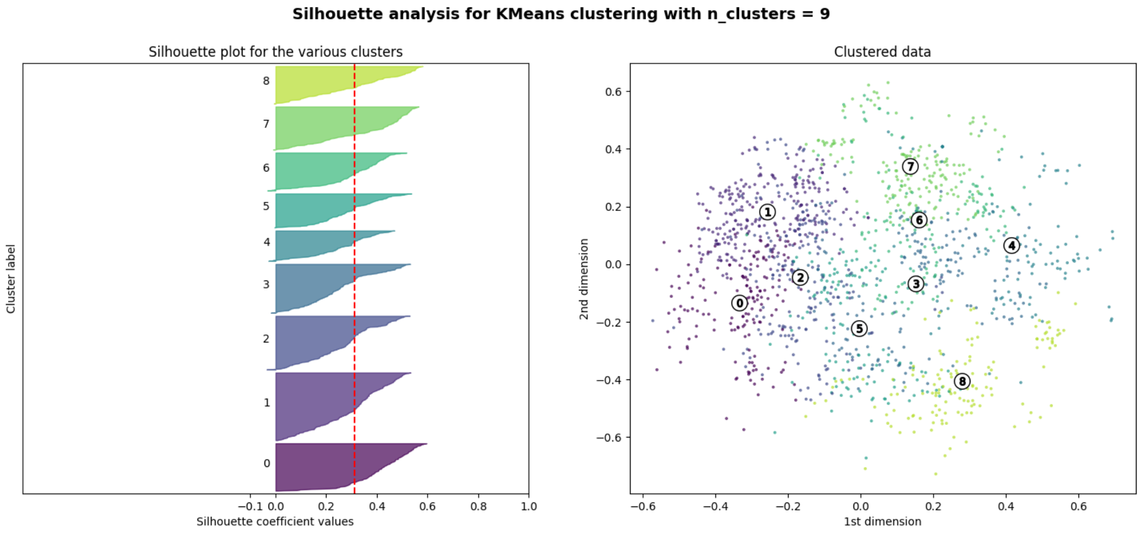
Silhouette analysis for KMeans clustering with n_clusters = 9
Silhouette plot for the various clusters
Cluster label
Silhouette coefficient values
Clustered data
1st dimension
2nd dimension

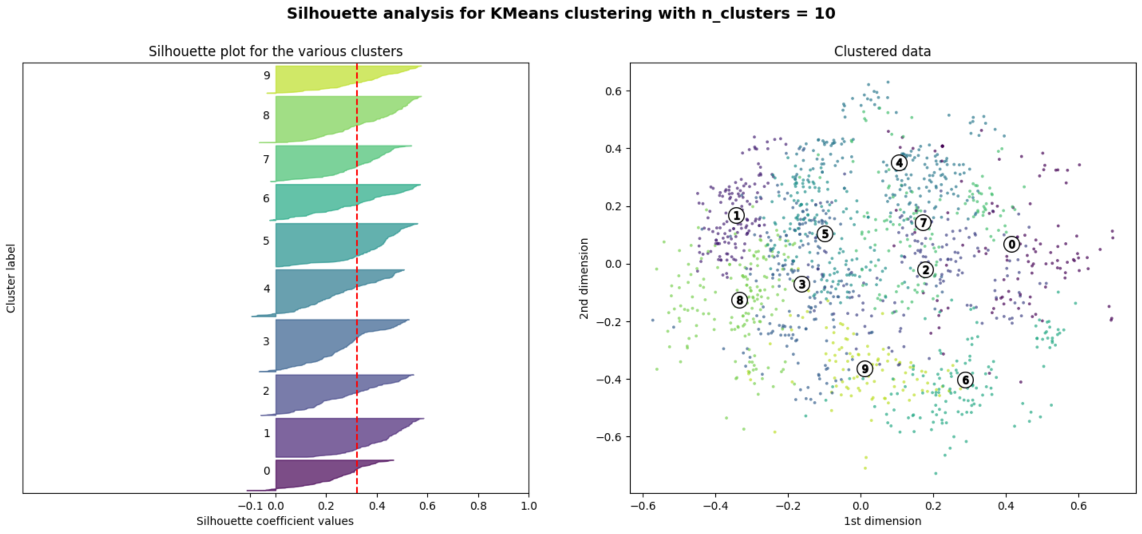
Silhouette analysis for KMeans clustering with n_clusters = 10
Silhouette plot for the various clusters
Cluster label
Silhouette coefficient values
Clustered data
1st dimension
2nd dimension

Silhouette analysis for KMeans clustering with n_clusters = 11

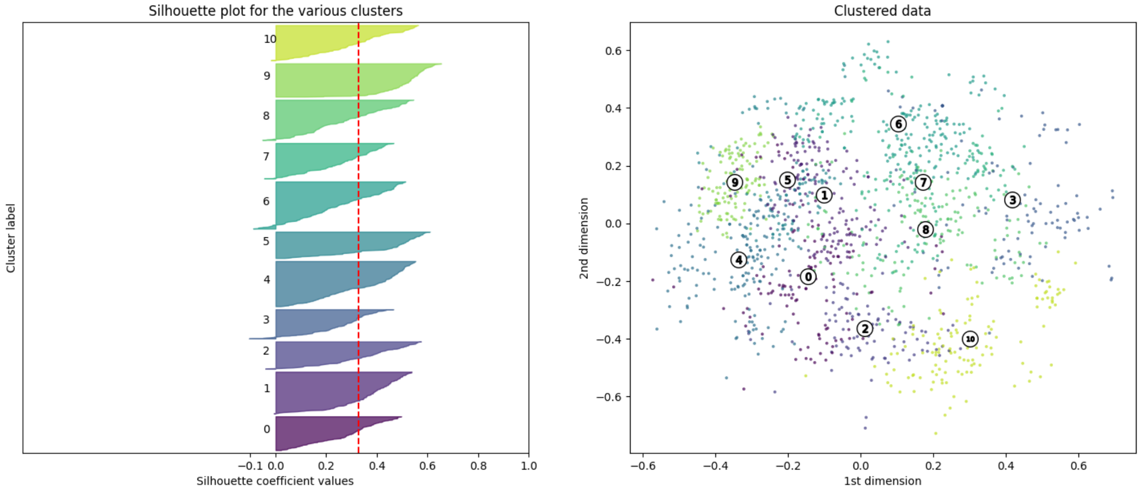

Silhouette analysis for KMeans clustering with n_clusters = 12

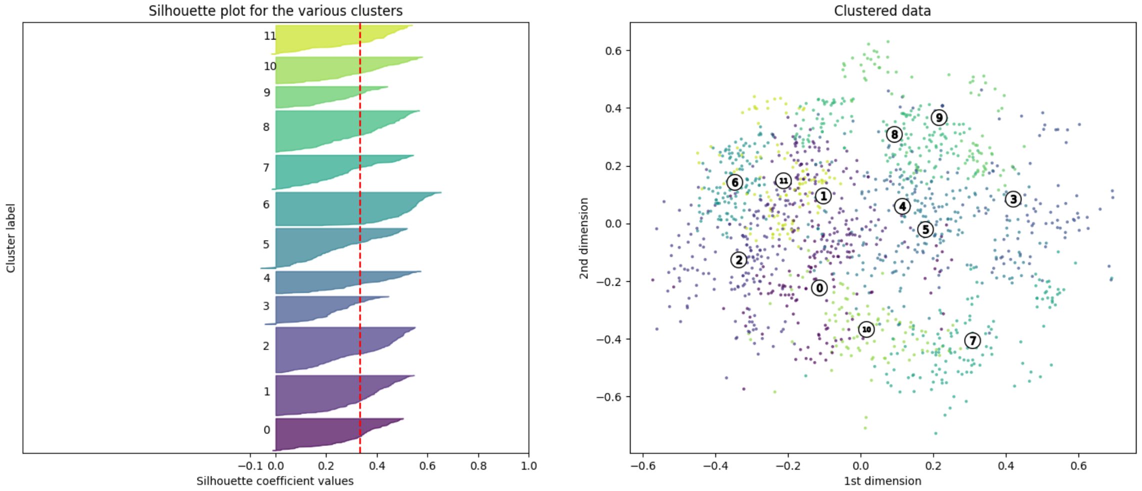

Silhouette analysis for KMeans clustering with n_clusters = 13

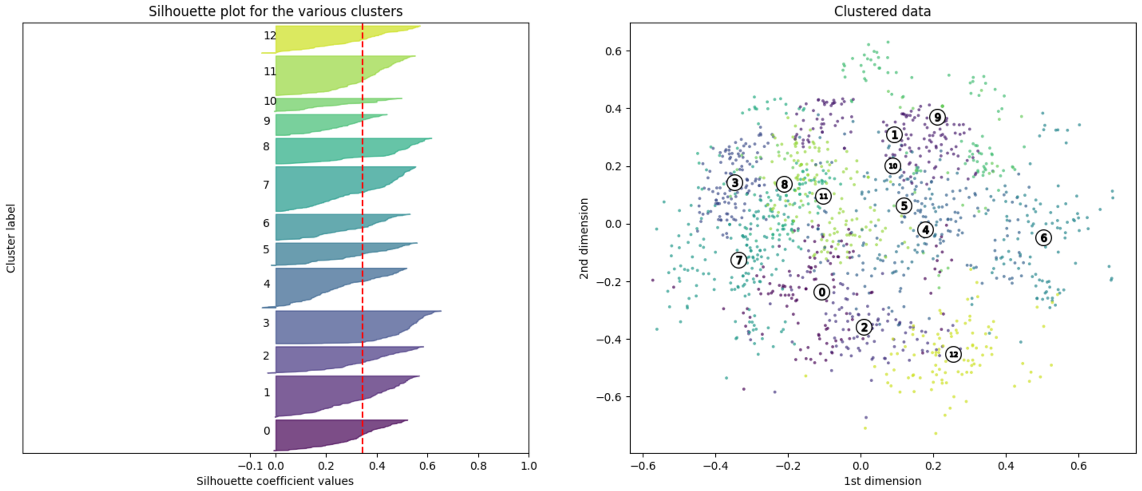

Silhouette analysis for KMeans clustering with n_clusters = 14

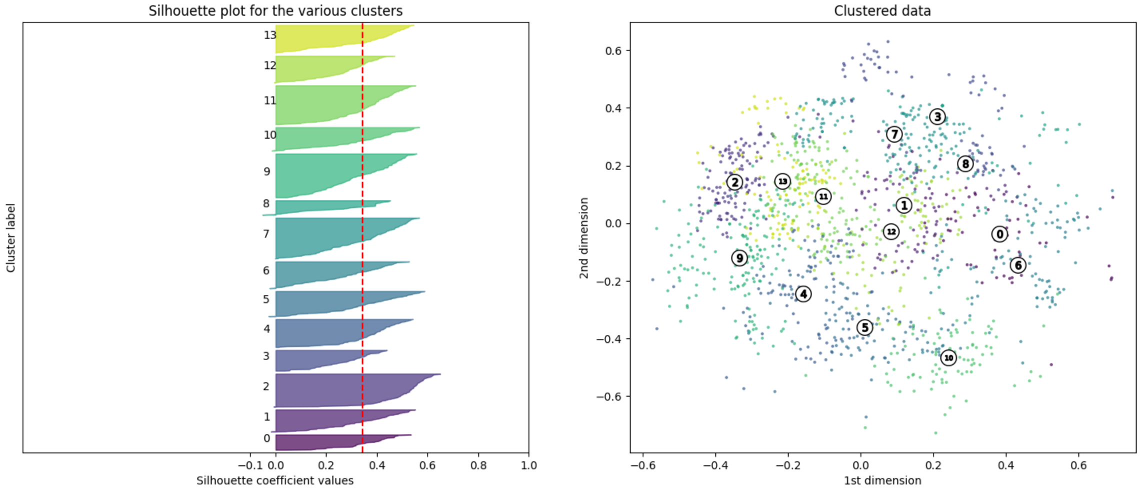

Silhouette analysis for KMeans clustering with n_clusters = 15

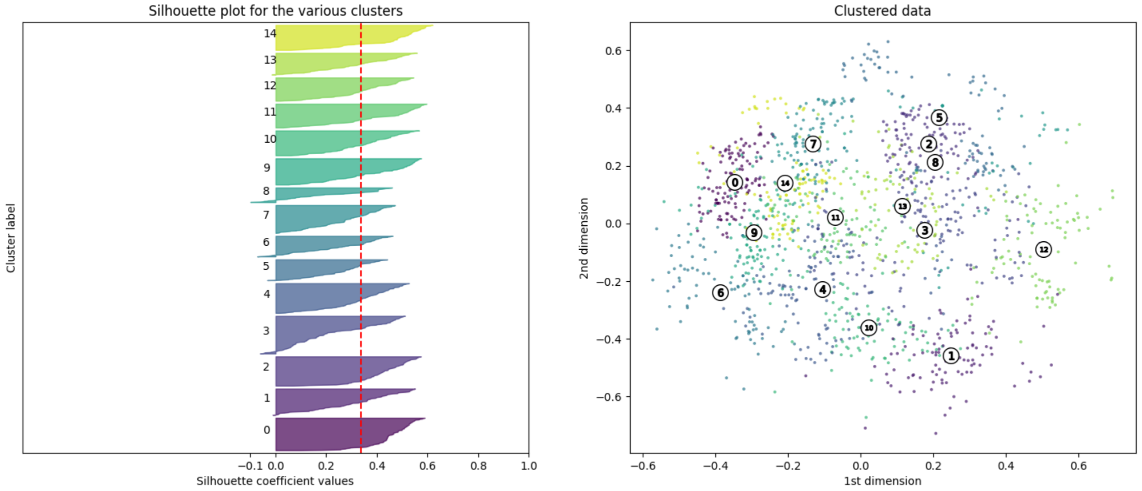

Silhouette analysis for KMeans clustering with n_clusters = 16

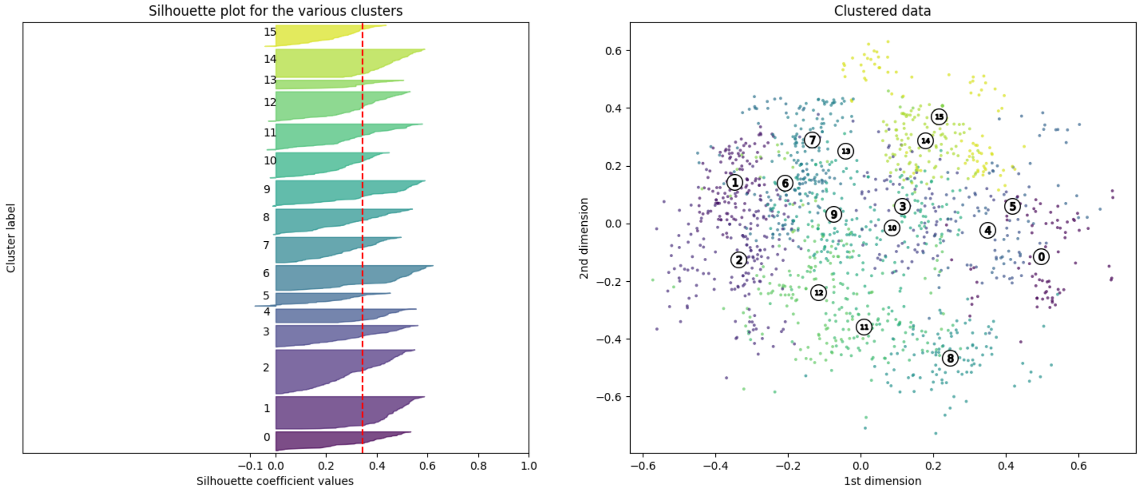

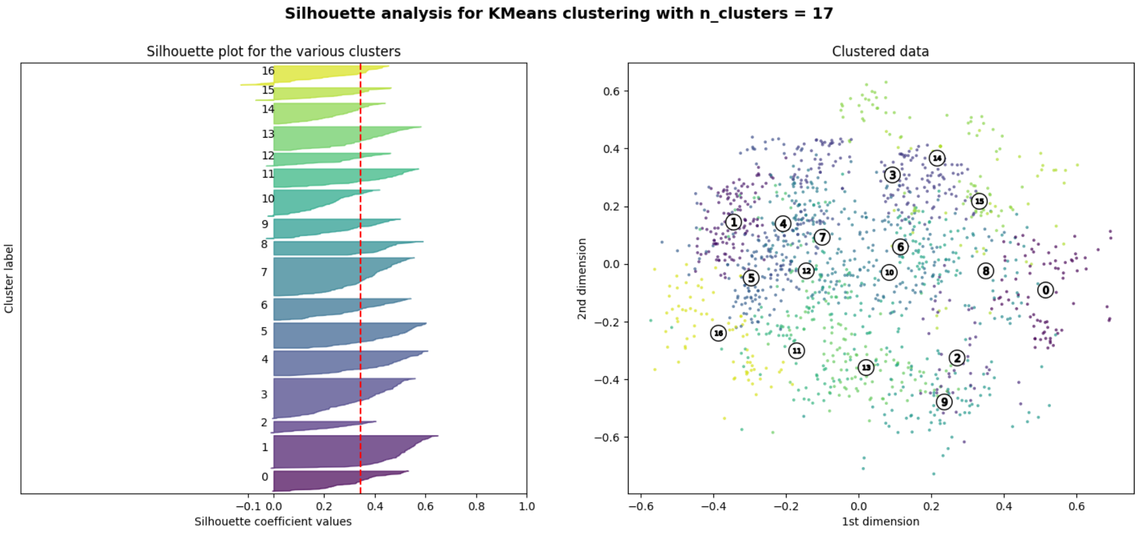
Silhouette analysis for KMeans clustering with n_clusters = 17
Silhouette plot for the various clusters
Cluster label
Silhouette coefficient values
Clustered data
1st dimension
2nd dimension

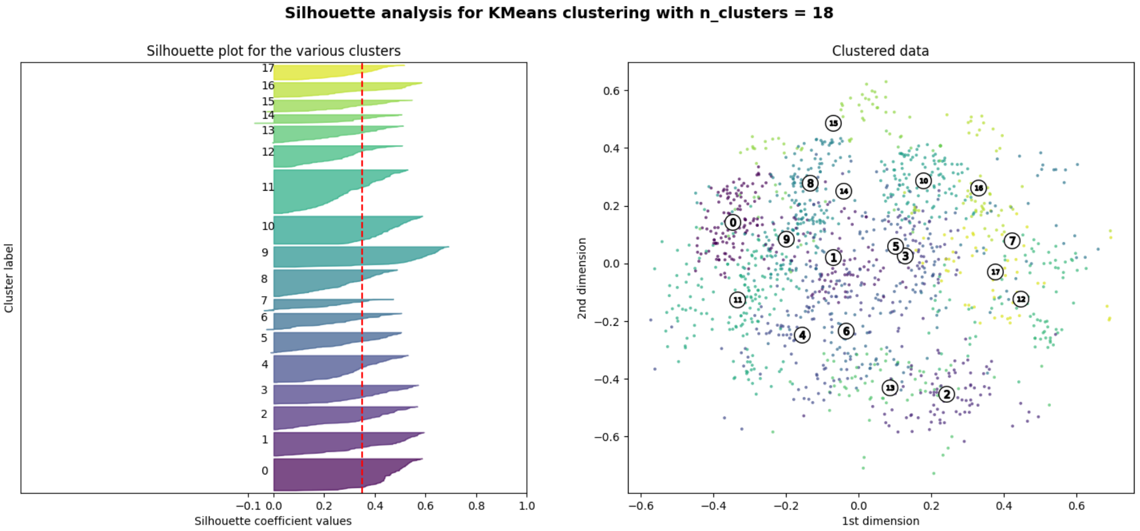
Silhouette analysis for KMeans clustering with n_clusters = 18
Silhouette plot for the various clusters
Cluster label
Silhouette coefficient values
Clustered data
1st dimension
2nd dimension

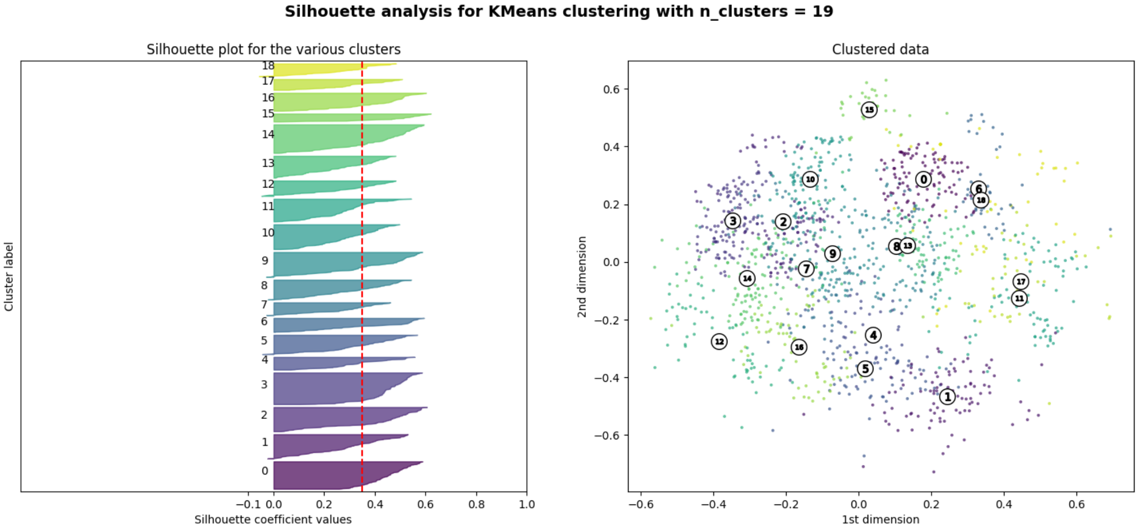
Silhouette analysis for KMeans clustering with n_clusters = 19
Silhouette plot for the various clusters
Cluster label
Silhouette coefficient values
Clustered data
1st dimension
2nd dimension
