# Supplementary material for: DIVIS: a semantic DIstance to improve the VISualisation of heterogeneous phenotypic datasets
Source: BioData Min. 2022 Apr 4;15:10. doi: 10.1186/s13040-022-00293-y (PMC8981856; doi:10.1186/s13040-022-00293-y)
Supplement: Supplementary file 8 — Additional file 8 Results from the catdes r function from the factoMiner package. Results from the catdes function, used to characterise the clusters with the modalities of the original variables in the datasets, clusters calculated with the KMeans algorithm, in the 6 clusters case, for both distances (semantic and Gower’s). [file 13040_2022_293_MOESM8_ESM.html]

CatDes


# Semantic

```
## 
## Link between the cluster variable and the categorical variables (chi-square test)
## =================================================================================
##                              p.value df
## Ploidy.level           1.976263e-323 25
## Duplicature.type       3.281953e-308 20
## Horticultural.group    4.523280e-265 85
## Geographic.origin      1.128432e-149 95
## Breeding.period         3.329061e-70 80
## Petal.colour            1.065882e-21 60
## Repeat.flowering.level  5.307822e-11 30
## Quantity.of.prickles    1.368707e-02 20
## 
## Description of each cluster by the categories
## =============================================
## $`0`
##                                                    Cla/Mod   Mod/Cla    Global      p.value    v.test
## Horticultural.group=chinese                     47.9166667 40.350877  6.694561 6.879904e-29 11.153558
## Horticultural.group=wild                        41.1764706 42.982456  8.298466 4.219606e-27 10.781283
## Duplicature.type=NA                             19.3820225 60.526316 24.825662 2.852645e-17  8.452445
## Ploidy.level=5                                  59.2592593 14.035088  1.882845 5.975132e-12  6.880241
## Breeding.period=NA                              40.7407407 19.298246  3.765690 9.287770e-12  6.817129
## Geographic.origin=eastern europe                65.0000000 11.403509  1.394700 1.310917e-10  6.425900
## Geographic.origin=china                         32.0000000 21.052632  5.230126 4.057899e-10  6.251784
## Breeding.period=<1700                           39.5348837 14.912281  2.998605 5.151189e-09  5.842213
## Petal.colour=NA                                 24.4897959 21.052632  6.834031 1.695256e-07  5.229983
## Ploidy.level=3                                  21.2121212 24.561404  9.205021 3.640562e-07  5.086852
## Ploidy.level=6                                  39.3939394 11.403509  2.301255 4.249748e-07  5.057416
## Geographic.origin=middle east                   42.8571429  7.894737  1.464435 1.329096e-05  4.355259
## Repeat.flowering.level=NA                        9.0460526 96.491228 84.797768 4.095286e-05  4.102037
## Horticultural.group=agathe                      71.4285714  4.385965  0.488145 5.533959e-05  4.031850
## Geographic.origin=europe                        33.3333333  7.017544  1.673640 3.565831e-04  3.570292
## Geographic.origin=asia                          17.3913043  7.017544  3.207810 3.337566e-02  2.127535
## Breeding.period=[1700-1799]                     16.6666667  7.017544  3.347280 4.275136e-02  2.026131
## Repeat.flowering.level=high                      0.0000000  0.000000  2.580195 4.479015e-02 -2.006620
## Geographic.origin=paris, france                  4.6808511  9.649123 16.387727 3.554261e-02 -2.102122
## Breeding.period=[1880-1889]                      3.8461538  5.263158 10.878661 3.444712e-02 -2.114799
## Repeat.flowering.level=very high                 0.0000000  0.000000  2.859135 3.185206e-02 -2.146262
## Breeding.period=[1850-1859]                      2.0833333  1.754386  6.694561 1.555932e-02 -2.419092
## Horticultural.group=hybrid tea                   1.2500000  0.877193  5.578801 1.015687e-02 -2.570443
## Breeding.period=[1900-1909]                      2.7586207  3.508772 10.111576 7.958469e-03 -2.653827
## Breeding.period=[1840-1849]                      2.2556391  2.631579  9.274756 4.819212e-03 -2.818876
## Duplicature.type=double                          5.2208835 22.807018 34.728033 4.395004e-03 -2.848325
## Horticultural.group=damas                        0.0000000  0.000000  4.951185 2.390292e-03 -3.036894
## Horticultural.group=bourbon                      0.0000000  0.000000  5.369596 1.413882e-03 -3.191802
## Horticultural.group=noisette                     0.0000000  0.000000  5.578801 1.086422e-03 -3.267134
## Horticultural.group=tea                          0.0000000  0.000000  6.206416 4.910884e-04 -3.485571
## Horticultural.group=moss                         0.0000000  0.000000  7.182706 1.412278e-04 -3.806008
## Geographic.origin=angers, france                 0.6329114  0.877193 11.018131 1.611048e-05 -4.312930
## Geographic.origin=lyon, france                   1.0582011  1.754386 13.179916 1.066785e-05 -4.403174
## Horticultural.group=continuous flowering hybrid  0.0000000  0.000000 12.900976 7.162816e-08 -5.387038
## Horticultural.group=NA                           1.8947368  7.894737 33.124128 4.817390e-11 -6.576473
## Duplicature.type=single                          0.5714286  1.754386 24.407252 2.742513e-12 -6.990324
## Ploidy.level=2                                   0.0000000  0.000000 29.637378 5.232634e-19 -8.907224
## 
## $`1`
##                                                   Cla/Mod    Mod/Cla    Global       p.value     v.test
## Duplicature.type=single                         70.000000 99.5934959 24.407252 4.680856e-190  29.405427
## Ploidy.level=4                                  25.000000 82.9268293 56.903766  3.550649e-21   9.445100
## Horticultural.group=bourbon                     36.363636 11.3821138  5.369596  3.217825e-05   4.157476
## Horticultural.group=continuous flowering hybrid 27.567568 20.7317073 12.900976  1.393265e-04   3.809361
## Breeding.period=<1700                           34.883721  6.0975610  2.998605  4.531954e-03   2.838546
## Horticultural.group=moss                        27.184466 11.3821138  7.182706  8.115931e-03   2.647208
## Ploidy.level=3                                  25.757576 13.8211382  9.205021  8.755968e-03   2.621438
## Breeding.period=[1840-1849]                     25.563910 13.8211382  9.274756  9.996046e-03   2.575966
## Breeding.period=[1800-1809]                     37.500000  3.6585366  1.673640  1.724622e-02   2.381416
## Repeat.flowering.level=very high                31.707317  5.2845528  2.859135  2.100584e-02   2.307880
## Geographic.origin=angers, france                23.417722 15.0406504 11.018131  3.240169e-02   2.139420
## Geographic.origin=lyon, france                  22.751323 17.4796748 13.179916  3.349430e-02   2.126108
## Horticultural.group=NA                          14.315789 27.6422764 33.124128  4.336363e-02  -2.020190
## Petal.colour=yellow                              7.042254  2.0325203  4.951185  1.332136e-02  -2.475061
## Breeding.period=[1890-1899]                     10.588235  7.3170732 11.854951  1.203329e-02  -2.511166
## Breeding.period=[1900-1909]                      9.655172  5.6910569 10.111576  8.149262e-03  -2.645822
## Horticultural.group=wild                         8.403361  4.0650407  8.298466  5.052287e-03  -2.803681
## Horticultural.group=chinese                      7.291667  2.8455285  6.694561  4.452589e-03  -2.844180
## Breeding.period=NA                               3.703704  0.8130081  3.765690  2.879691e-03  -2.980297
## Repeat.flowering.level=NA                       15.707237 77.6422764 84.797768  9.976582e-04  -3.291186
## Horticultural.group=tea                          4.494382  1.6260163  6.206416  2.605843e-04  -3.651627
## Geographic.origin=asia                           0.000000  0.0000000  3.207810  1.492216e-04  -3.792361
## Geographic.origin=china                          2.666667  0.8130081  5.230126  8.297294e-05  -3.935647
## Duplicature.type=half-double                     0.000000  0.0000000 15.969317  2.657732e-21  -9.475387
## Duplicature.type=NA                              0.000000  0.0000000 24.825662  1.062287e-34 -12.287116
## Ploidy.level=2                                   0.000000  0.0000000 29.637378  9.337344e-43 -13.706075
## Duplicature.type=double                          0.000000  0.0000000 34.728033  5.297961e-52 -15.173494
## 
## $`2`
##                                                   Cla/Mod    Mod/Cla    Global       p.value     v.test
## Ploidy.level=4                                  68.014706 85.5161787 56.903766  3.483976e-94  20.588441
## Duplicature.type=double                         61.646586 47.3035439 34.728033  9.140719e-20   9.098715
## Horticultural.group=continuous flowering hybrid 69.729730 19.8767334 12.900976  7.193650e-13   7.175703
## Duplicature.type=NA                             59.831461 32.8197227 24.825662  2.073230e-10   6.355816
## Horticultural.group=moss                        72.815534 11.5562404  7.182706  4.981954e-09   5.847774
## Geographic.origin=angers, france                67.088608 16.3328197 11.018131  5.335051e-09   5.836370
## Geographic.origin=paris, france                 61.276596 22.1879815 16.387727  7.577784e-08   5.376903
## Horticultural.group=damas                       76.056338  8.3204931  4.951185  7.682120e-08   5.374440
## Breeding.period=[1850-1859]                     70.833333 10.4776579  6.694561  1.870186e-07   5.211798
## Horticultural.group=hybrid tea                  71.250000  8.7827427  5.578801  1.558338e-06   4.803605
## Breeding.period=[1840-1849]                     61.654135 12.6348228  9.274756  7.258130e-05   3.967662
## Repeat.flowering.level=high                     75.675676  4.3143297  2.580195  1.723917e-04   3.756376
## Duplicature.type=half-double                    56.331878 19.8767334 15.969317  2.564399e-04   3.655741
## Breeding.period=[1810-1819]                     63.218391  8.4745763  6.066946  5.606396e-04   3.449974
## Breeding.period=[1860-1869]                     60.909091 10.3235747  7.670851  6.482249e-04   3.410590
## Petal.colour=red                                54.651163 21.7257319 17.991632  8.602198e-04   3.332654
## Repeat.flowering.level=medium/high              72.727273  3.6979969  2.301255  1.468520e-03   3.180833
## Petal.colour=pink                               49.550898 51.0015408 46.582985  2.317373e-03   3.046221
## Repeat.flowering.level=very high                68.292683  4.3143297  2.859135  2.906538e-03   2.977453
## Horticultural.group=NA                          50.736842 37.1340524 33.124128  3.429717e-03   2.926344
## Geographic.origin=north west, france            66.666667  4.3143297  2.928870  5.124158e-03   2.799123
## Repeat.flowering.level=low/medium               65.217391  4.6224961  3.207810  6.233028e-03   2.735264
## Horticultural.group=bourbon                     59.740260  7.0878274  5.369596  9.262385e-03   2.602215
## Geographic.origin=western europe                53.703704 13.4052388 11.297071  2.258366e-02   2.280405
## Geographic.origin=northern europe               54.545455 11.0939908  9.205021  2.537273e-02   2.235680
## Geographic.origin=south west, france            20.000000  0.4622496  1.046025  4.969841e-02  -1.962551
## Geographic.origin=europe                        25.000000  0.9244992  1.673640  4.469064e-02  -2.007555
## Ploidy.level=5                                  25.925926  1.0785824  1.882845  4.169706e-02  -2.036531
## Geographic.origin=orleans, france               26.470588  1.3867488  2.370990  2.537404e-02  -2.235660
## Geographic.origin=lyon, france                  37.566138 10.9399076 13.179916  2.237171e-02  -2.283997
## Geographic.origin=eastern europe                20.000000  0.6163328  1.394700  2.162325e-02  -2.296921
## Horticultural.group=bengale                      0.000000  0.0000000  0.488145  1.455335e-02  -2.443307
## Horticultural.group=agathe                       0.000000  0.0000000  0.488145  1.455335e-02  -2.443307
## Breeding.period=[1800-1809]                     20.833333  0.7704160  1.673640  1.446846e-02  -2.445417
## Geographic.origin=middle east                   19.047619  0.6163328  1.464435  1.413024e-02  -2.453935
## Breeding.period=[1890-1899]                     34.705882  9.0909091 11.854951  3.091556e-03  -2.958485
## Breeding.period=<1700                           23.255814  1.5408320  2.998605  2.805247e-03  -2.988310
## Geographic.origin=south east, france            23.913043  1.6949153  3.207810  2.699470e-03  -3.000037
## Geographic.origin=america                       27.027027  3.0816641  5.160391  1.044062e-03  -3.278376
## Horticultural.group=noisette                    27.500000  3.3898305  5.578801  8.793309e-04  -3.326535
## Petal.colour=NA                                 27.551020  4.1602465  6.834031  2.151748e-04  -3.700500
## Petal.colour=white                              33.333333 10.7858243 14.644351  1.539401e-04  -3.784626
## Breeding.period=[1900-1909]                     30.344828  6.7796610 10.111576  1.197552e-04  -3.846627
## Petal.colour=yellow                             15.492958  1.6949153  4.951185  6.590907e-08  -5.401981
## Repeat.flowering.level=NA                       41.858553 78.4283513 84.797768  1.097539e-09  -6.094538
## Breeding.period=NA                               7.407407  0.6163328  3.765690  7.195353e-10  -6.161732
## Geographic.origin=asia                           2.173913  0.1540832  3.207810  2.100639e-11  -6.698852
## Horticultural.group=tea                          8.988764  1.2326656  6.206416  2.115913e-14  -7.643381
## Geographic.origin=china                          0.000000  0.0000000  5.230126  4.396924e-21  -9.422686
## Horticultural.group=chinese                      0.000000  0.0000000  6.694561  4.567804e-27 -10.773987
## Horticultural.group=wild                         0.000000  0.0000000  8.298466  8.945527e-34 -12.113622
## Duplicature.type=single                          0.000000  0.0000000 24.407252 1.894585e-112 -22.534761
## Ploidy.level=2                                   0.000000  0.0000000 29.637378 5.387593e-144 -25.550741
## 
## $`3`
##                                                   Cla/Mod     Mod/Cla     Global       p.value     v.test
## Ploidy.level=2                                  54.352941 100.0000000 29.6373780 2.703221e-148  25.934737
## Horticultural.group=tea                         59.550562  22.9437229  6.2064156  3.169318e-22   9.694905
## Duplicature.type=double                         26.305221  56.7099567 34.7280335  8.133074e-14   7.468149
## Breeding.period=[1900-1909]                     37.931034  23.8095238 10.1115760  1.332603e-11   6.765053
## Horticultural.group=NA                          25.263158  51.9480519 33.1241283  1.073100e-10   6.456277
## Horticultural.group=noisette                    43.750000  15.1515152  5.5788006  1.567858e-09   6.037226
## Petal.colour=yellow                             40.845070  12.5541126  4.9511855  3.176565e-07   5.112658
## Repeat.flowering.level=NA                       18.009868  94.8051948 84.7977685  3.761944e-07   5.080625
## Geographic.origin=orleans, france               52.941176   7.7922078  2.3709902  6.485030e-07   4.976183
## Breeding.period=[1890-1899]                     27.647059  20.3463203 11.8549512  4.472562e-05   4.081606
## Duplicature.type=half-double                    24.890830  24.6753247 15.9693166  1.726327e-04   3.756026
## Geographic.origin=south east, france            36.956522   7.3593074  3.2078103  5.143460e-04   3.473172
## Geographic.origin=lyon, france                  24.867725  20.3463203 13.1799163  8.341973e-04   3.341191
## Geographic.origin=south west, france            53.333333   3.4632035  1.0460251  1.057642e-03   3.274727
## Breeding.period=[1880-1889]                     24.358974  16.4502165 10.8786611  4.713106e-03   2.826015
## Geographic.origin=america                       28.378378   9.0909091  5.1603905  6.243954e-03   2.734687
## Breeding.period=[1910-1914]                     38.095238   3.4632035  1.4644351  1.524716e-02   2.426454
## Geographic.origin=western europe                22.839506  16.0173160 11.2970711  1.744445e-02   2.377204
## Geographic.origin=northern europe               22.727273  12.9870130  9.2050209  3.690836e-02   2.086776
## Petal.colour=white                              20.952381  19.0476190 14.6443515  4.426005e-02   2.011620
## Horticultural.group=ayrshire                    44.444444   1.7316017  0.6276151  4.995393e-02   1.960358
## Geographic.origin=north west, france             4.761905   0.8658009  2.9288703  2.920776e-02  -2.180671
## Geographic.origin=eastern europe                 0.000000   0.0000000  1.3947001  2.905154e-02  -2.182786
## Repeat.flowering.level=low/medium                4.347826   0.8658009  3.2078103  1.648044e-02  -2.398098
## Duplicature.type=NA                             12.078652  18.6147186 24.8256625  1.516181e-02  -2.428491
## Petal.colour=mauve_purple                        7.142857   2.5974026  5.8577406  1.429296e-02  -2.449815
## Geographic.origin=europe                         0.000000   0.0000000  1.6736402  1.422165e-02  -2.451616
## Breeding.period=[1800-1809]                      0.000000   0.0000000  1.6736402  1.422165e-02  -2.451616
## Geographic.origin=NA                             7.368421   3.0303030  6.6248257  1.078861e-02  -2.549472
## Ploidy.level=5                                   0.000000   0.0000000  1.8828452  8.310991e-03  -2.639167
## Breeding.period=<1700                            2.325581   0.4329004  2.9986053  4.893398e-03  -2.813969
## Breeding.period=[1810-1819]                      5.747126   2.1645022  6.0669456  3.191914e-03  -2.948625
## Ploidy.level=6                                   0.000000   0.0000000  2.3012552  2.827614e-03  -2.985882
## Horticultural.group=hybrid tea                   5.000000   1.7316017  5.5788006  2.254263e-03  -3.054513
## Petal.colour=red                                 9.689922  10.8225108 17.9916318  1.249237e-03  -3.227393
## Breeding.period=[1850-1859]                      5.208333   2.1645022  6.6945607  9.497560e-04  -3.305002
## Repeat.flowering.level=very high                 0.000000   0.0000000  2.8591353  6.663179e-04  -3.403076
## Geographic.origin=paris, france                  8.936170   9.0909091 16.3877266  5.873492e-04  -3.437390
## Geographic.origin=angers, france                 6.962025   4.7619048 11.0181311  3.584213e-04  -3.568945
## Breeding.period=[1700-1799]                      0.000000   0.0000000  3.3472803  1.867070e-04  -3.736355
## Horticultural.group=bourbon                      2.597403   0.8658009  5.3695955  1.386067e-04  -3.810641
## Breeding.period=[1860-1869]                      4.545455   2.1645022  7.6708508  1.311192e-04  -3.824347
## Horticultural.group=damas                        1.408451   0.4329004  4.9511855  4.474969e-05  -4.081481
## Geographic.origin=china                          1.333333   0.4329004  5.2301255  2.239454e-05  -4.239573
## Breeding.period=[1840-1849]                      4.511278   2.5974026  9.2747559  1.990597e-05  -4.265943
## Horticultural.group=chinese                      0.000000   0.0000000  6.6945607  2.500010e-08  -5.573270
## Horticultural.group=moss                         0.000000   0.0000000  7.1827057  6.612336e-09  -5.800485
## Horticultural.group=continuous flowering hybrid  2.702703   2.1645022 12.9009763  7.695576e-10  -6.151083
## Horticultural.group=wild                         0.000000   0.0000000  8.2984658  3.072523e-10  -6.295074
## Ploidy.level=3                                   0.000000   0.0000000  9.2050209  2.462140e-11  -6.675605
## Duplicature.type=single                          0.000000   0.0000000 24.4072524  8.998687e-32 -11.729502
## Ploidy.level=4                                   0.000000   0.0000000 56.9037657  3.298130e-98 -21.032635
## 
## $`4`
##                                                    Cla/Mod    Mod/Cla     Global      p.value     v.test
## Ploidy.level=2                                  21.1764706 100.000000 29.6373780 1.949822e-51  15.087749
## Geographic.origin=asia                          60.8695652  31.111111  3.2078103 3.360013e-24  10.148643
## Horticultural.group=chinese                     37.5000000  40.000000  6.6945607 7.597931e-22   9.605242
## Horticultural.group=wild                        32.7731092  43.333333  8.2984658 2.637755e-21   9.476175
## Geographic.origin=china                         40.0000000  33.333333  5.2301255 5.909471e-19   8.893723
## Breeding.period=NA                              31.4814815  18.888889  3.7656904 6.499442e-09   5.803372
## Petal.colour=white                              14.7619048  34.444444 14.6443515 1.124659e-06   4.868470
## Horticultural.group=bengale                     71.4285714   5.555556  0.4881450 1.696128e-05   4.301543
## Breeding.period=[1800-1809]                     29.1666667   7.777778  1.6736402 4.939933e-04   3.483993
## Duplicature.type=half-double                    11.3537118  28.888889 15.9693166 1.399081e-03   3.194841
## Breeding.period=[1700-1799]                     18.7500000  10.000000  3.3472803 2.733295e-03   2.996242
## Quantity.of.prickles=medium                     28.5714286   4.444444  0.9762901 1.004743e-02   2.574193
## Repeat.flowering.level=NA                        6.9078947  93.333333 84.7977685 1.304750e-02   2.482470
## Repeat.flowering.level=very low                 17.6470588   6.666667  2.3709902 2.071450e-02   2.313148
## Petal.colour=NA                                 12.2448980  13.333333  6.8340307 2.241500e-02   2.283261
## Repeat.flowering.level=low/medium                0.0000000   0.000000  3.2078103 4.826637e-02  -1.975016
## Geographic.origin=south east, france             0.0000000   0.000000  3.2078103 4.826637e-02  -1.975016
## Petal.colour=red                                 3.4883721  10.000000 17.9916318 3.387375e-02  -2.121571
## Horticultural.group=damas                        0.0000000   0.000000  4.9511855 8.895828e-03  -2.616032
## Quantity.of.prickles=NA                          5.9743954  93.333333 98.0474198 7.550123e-03  -2.671553
## Horticultural.group=bourbon                      0.0000000   0.000000  5.3695955 5.899875e-03  -2.753295
## Horticultural.group=noisette                     0.0000000   0.000000  5.5788006 4.801366e-03  -2.820067
## Horticultural.group=hybrid tea                   0.0000000   0.000000  5.5788006 4.801366e-03  -2.820067
## Horticultural.group=tea                          0.0000000   0.000000  6.2064156 2.580446e-03  -3.013745
## Geographic.origin=northern europe                0.7575758   1.111111  9.2050209 1.474337e-03  -3.179688
## Horticultural.group=moss                         0.0000000   0.000000  7.1827057 9.738074e-04  -3.297987
## Geographic.origin=western europe                 0.6172840   1.111111 11.2970711 2.036218e-04  -3.714480
## Ploidy.level=3                                   0.0000000   0.000000  9.2050209 1.250084e-04  -3.836090
## Petal.colour=pink                                3.4431138  25.555556 46.5829847 2.656842e-05  -4.201046
## Geographic.origin=angers, france                 0.0000000   0.000000 11.0181311 1.905290e-05  -4.275710
## Horticultural.group=continuous flowering hybrid  0.0000000   0.000000 12.9009763 2.588698e-06  -4.701016
## Geographic.origin=paris, france                  0.4255319   1.111111 16.3877266 1.197992e-06  -4.855969
## Horticultural.group=NA                           1.4736842   7.777778 33.1241283 5.981055e-09  -5.817287
## Duplicature.type=single                          0.0000000   0.000000 24.4072524 4.482860e-12  -6.921057
## Ploidy.level=4                                   0.0000000   0.000000 56.9037657 2.384486e-35 -12.407338
## 
## $`5`
##                                                    Cla/Mod     Mod/Cla     Global      p.value     v.test
## Duplicature.type=single                         29.4285714  99.0384615 24.4072524 1.608493e-67  17.361714
## Ploidy.level=2                                  24.4705882 100.0000000 29.6373780 5.661701e-60  16.333930
## Horticultural.group=tea                         26.9662921  23.0769231  6.2064156 2.799704e-09   5.942931
## Repeat.flowering.level=NA                        8.4703947  99.0384615 84.7977685 3.932215e-07   5.072210
## Geographic.origin=china                         24.0000000  17.3076923  5.2301255 2.685423e-06   4.693521
## Petal.colour=yellow                             22.5352113  15.3846154  4.9511855 2.587425e-05   4.207035
## Horticultural.group=wild                        17.6470588  20.1923077  8.2984658 7.011915e-05   3.975881
## Breeding.period=[1890-1899]                     13.5294118  22.1153846 11.8549512 2.078796e-03   3.078738
## Breeding.period=[1900-1909]                     13.1034483  18.2692308 10.1115760 8.422663e-03   2.634640
## Petal.colour=white                              11.4285714  23.0769231 14.6443515 1.714393e-02   2.383606
## Geographic.origin=south east, france            17.3913043   7.6923077  3.2078103 1.949192e-02   2.335987
## Horticultural.group=ayrshire                    33.3333333   2.8846154  0.6276151 2.504595e-02   2.240693
## Repeat.flowering.level=very high                 0.0000000   0.0000000  2.8591353 4.361084e-02  -2.017812
## Breeding.period=<1700                            0.0000000   0.0000000  2.9986053 3.733987e-02  -2.082028
## Breeding.period=[1840-1849]                      3.0075188   3.8461538  9.2747559 3.507232e-02  -2.107522
## Repeat.flowering.level=low/medium                0.0000000   0.0000000  3.2078103 2.957000e-02  -2.175803
## Breeding.period=[1850-1859]                      2.0833333   1.9230769  6.6945607 2.806881e-02  -2.196323
## Horticultural.group=bourbon                      1.2987013   0.9615385  5.3695955 2.157144e-02  -2.297830
## Horticultural.group=hybrid tea                   1.2500000   0.9615385  5.5788006 1.751501e-02  -2.375715
## Breeding.period=[1810-1819]                      1.1494253   0.9615385  6.0669456 1.071697e-02  -2.551794
## Geographic.origin=paris, france                  3.4042553   7.6923077 16.3877266 8.228404e-03  -2.642551
## Petal.colour=pink                                5.2395210  33.6538462 46.5829847 5.849461e-03  -2.756103
## Geographic.origin=angers, france                 1.8987342   2.8846154 11.0181311 2.158519e-03  -3.067508
## Horticultural.group=moss                         0.0000000   0.0000000  7.1827057 3.171250e-04  -3.600894
## Ploidy.level=3                                   0.0000000   0.0000000  9.2050209 2.918507e-05  -4.179734
## Horticultural.group=continuous flowering hybrid  0.0000000   0.0000000 12.9009763 3.220563e-07  -5.110060
## Duplicature.type=half-double                     0.0000000   0.0000000 15.9693166 6.546408e-09  -5.802165
## Duplicature.type=NA                              0.2808989   0.9615385 24.8256625 1.397171e-12  -7.084331
## Duplicature.type=double                          0.0000000   0.0000000 34.7280335 6.486510e-21  -9.381785
## Ploidy.level=4                                   0.0000000   0.0000000 56.9037657 4.473917e-41 -13.422335
```

# Gower

```
## 
## Link between the cluster variable and the categorical variables (chi-square test)
## =================================================================================
##                              p.value df
## Ploidy.level           5.856364e-281 25
## Duplicature.type       3.577006e-271 20
## Petal.colour           4.763602e-193 60
## Horticultural.group     4.368923e-75 85
## Geographic.origin       3.117025e-58 95
## Breeding.period         4.026281e-39 80
## Repeat.flowering.level  3.943427e-09 30
## 
## Description of each cluster by the categories
## =============================================
## $`0`
##                                                   Cla/Mod    Mod/Cla     Global       p.value     v.test
## Duplicature.type=double                         57.028112 97.9310345 34.7280335 2.084045e-151  26.209261
## Ploidy.level=4                                  34.313725 96.5517241 56.9037657  5.510083e-65  17.023375
## Petal.colour=red                                36.434109 32.4137931 17.9916318  1.394490e-11   6.758478
## Horticultural.group=continuous flowering hybrid 35.135135 22.4137931 12.9009763  3.499587e-07   5.094340
## Breeding.period=[1840-1849]                     34.586466 15.8620690  9.2747559  4.723302e-05   4.068913
## Geographic.origin=paris, france                 28.936170 23.4482759 16.3877266  4.534720e-04   3.506834
## Breeding.period=[1860-1869]                     33.636364 12.7586207  7.6708508  5.980165e-04   3.432512
## Horticultural.group=hybrid tea                  36.250000 10.0000000  5.5788006  6.276727e-04   3.419366
## Repeat.flowering.level=high                     43.243243  5.5172414  2.5801953  1.385874e-03   3.197577
## Horticultural.group=moss                        32.038835 11.3793103  7.1827057  3.328349e-03   2.935664
## Horticultural.group=damas                       33.802817  8.2758621  4.9511855  6.061062e-03   2.744460
## Breeding.period=[1810-1819]                     32.183908  9.6551724  6.0669456  6.694842e-03   2.711654
## Geographic.origin=north west, france            38.095238  5.5172414  2.9288703  6.980264e-03   2.697784
## Repeat.flowering.level=medium/high              39.393939  4.4827586  2.3012552  1.104090e-02   2.541402
## Geographic.origin=NA                            30.526316 10.0000000  6.6248257  1.363594e-02   2.466713
## Repeat.flowering.level=very high                36.585366  5.1724138  2.8591353  1.415563e-02   2.453290
## Geographic.origin=angers, france                27.848101 15.1724138 11.0181311  1.450083e-02   2.444611
## Quantity.of.prickles=medium                      0.000000  0.0000000  0.9762901  4.161406e-02  -2.037359
## Geographic.origin=lyon, france                  14.285714  9.3103448 13.1799163  2.557175e-02  -2.232654
## Bush.height=very high                           13.286713  6.5517241  9.9721060  2.504288e-02  -2.240741
## Petal.colour=yellow                              9.859155  2.4137931  4.9511855  1.910480e-02  -2.343480
## Ploidy.level=5                                   3.703704  0.3448276  1.8828452  1.895673e-02  -2.346381
## Geographic.origin=south east, france             4.347826  0.6896552  3.2078103  2.490528e-03  -3.024490
## Breeding.period=NA                               3.703704  0.6896552  3.7656904  5.091219e-04  -3.475911
## Repeat.flowering.level=NA                       18.256579 76.5517241 84.7977685  2.851415e-05  -4.185020
## Geographic.origin=asia                           0.000000  0.0000000  3.2078103  2.540563e-05  -4.211166
## Horticultural.group=chinese                      5.208333  1.7241379  6.6945607  2.261229e-05  -4.237400
## Geographic.origin=china                          2.666667  0.6896552  5.2301255  6.445479e-06  -4.511223
## Horticultural.group=noisette                     2.500000  0.6896552  5.5788006  2.199434e-06  -4.734179
## Horticultural.group=wild                         5.042017  2.0689655  8.2984658  1.343237e-06  -4.833247
## Breeding.period=[1890-1899]                      7.058824  4.1379310 11.8549512  6.320586e-07  -4.981155
## Horticultural.group=tea                          2.247191  0.6896552  6.2064156  3.086062e-07  -5.118114
## Petal.colour=white                               6.190476  4.4827586 14.6443515  1.435065e-09  -6.051496
## Ploidy.level=3                                   0.000000  0.0000000  9.2050209  2.159553e-14  -7.640753
## Duplicature.type=half-double                     0.000000  0.0000000 15.9693166  1.768877e-25 -10.432125
## Duplicature.type=NA                              1.685393  2.0689655 24.8256625  7.986052e-32 -11.739603
## Duplicature.type=single                          0.000000  0.0000000 24.4072524  7.597820e-41 -13.383037
## Ploidy.level=2                                   0.000000  0.0000000 29.6373780  2.044161e-51 -15.084630
## 
## $`1`
##                                                   Cla/Mod    Mod/Cla     Global      p.value     v.test
## Ploidy.level=2                                  56.235294 72.2054381 29.6373780 4.966698e-78  18.699825
## Petal.colour=white                              76.666667 48.6404834 14.6443515 1.774485e-74  18.258389
## Geographic.origin=asia                          82.608696 11.4803625  3.2078103 5.047146e-18   8.652301
## Duplicature.type=half-double                    44.541485 30.8157100 15.9693166 2.554309e-15   7.910951
## Petal.colour=yellow                             61.971831 13.2930514  4.9511855 6.917707e-13   7.181052
## Horticultural.group=wild                        51.260504 18.4290030  8.2984658 2.472205e-12   7.004867
## Repeat.flowering.level=NA                       25.986842 95.4682779 84.7977685 1.090126e-11   6.794071
## Breeding.period=[1900-1909]                     42.758621 18.7311178 10.1115760 2.706914e-08   5.559408
## Geographic.origin=china                         48.000000 10.8761329  5.2301255 1.274126e-06   4.843748
## Horticultural.group=noisette                    40.000000  9.6676737  5.5788006 5.166685e-04   3.471962
## Breeding.period=NA                              42.592593  6.9486405  3.7656904 1.248643e-03   3.227529
## Petal.colour=NA                                 36.734694 10.8761329  6.8340307 1.597874e-03   3.156294
## Horticultural.group=tea                         37.078652  9.9697885  6.2064156 2.150661e-03   3.068598
## Petal.colour=orange                             48.275862  4.2296073  2.0223152 2.985987e-03   2.969177
## Horticultural.group=interspé                    50.000000  3.6253776  1.6736402 4.189434e-03   2.863534
## Geographic.origin=northern europe               32.575758 12.9909366  9.2050209 8.778713e-03   2.620554
## Breeding.period=[1890-1899]                     30.588235 15.7099698 11.8549512 1.611772e-02   2.406239
## Horticultural.group=ayrshire                    55.555556  1.5105740  0.6276151 4.142840e-02   2.039217
## Geographic.origin=orleans, france               38.235294  3.9274924  2.3709902 4.570651e-02   1.998094
## Bush.height=low                                 16.860465  8.7613293 11.9944212 3.537280e-02  -2.104065
## Horticultural.group=hybrid tea                  12.500000  3.0211480  5.5788006 1.594721e-02  -2.410121
## Repeat.flowering.level=medium/high               6.060606  0.6042296  2.3012552 1.125006e-02  -2.534834
## Geographic.origin=NA                            12.631579  3.6253776  6.6248257 8.975647e-03  -2.612980
## Repeat.flowering.level=high                      5.405405  0.6042296  2.5801953 4.673292e-03  -2.828731
## Breeding.period=[1840-1849]                     12.781955  5.1359517  9.2747559 1.940375e-03  -3.099210
## Petal.colour=red                                15.891473 12.3867069 17.9916318 1.882510e-03  -3.108168
## Geographic.origin=north west, france             4.761905  0.6042296  2.9288703 1.514630e-03  -3.171866
## Breeding.period=[1850-1859]                      9.375000  2.7190332  6.6945607 3.913915e-04  -3.545821
## Horticultural.group=bourbon                      7.792208  1.8126888  5.3695955 3.681843e-04  -3.561897
## Repeat.flowering.level=low/medium                2.173913  0.3021148  3.2078103 7.505989e-05  -3.959649
## Geographic.origin=angers, france                10.759494  5.1359517 11.0181311 3.325533e-05  -4.149946
## Horticultural.group=moss                         7.766990  2.4169184  7.1827057 2.868951e-05  -4.183627
## Repeat.flowering.level=very high                 0.000000  0.0000000  2.8591353 1.781975e-05  -4.290592
## Geographic.origin=paris, france                 11.914894  8.4592145 16.3877266 2.811538e-06  -4.684128
## Breeding.period=[1860-1869]                      5.454545  1.8126888  7.6708508 2.959640e-07  -5.125999
## Ploidy.level=3                                   6.060606  2.4169184  9.2050209 5.590700e-08  -5.431421
## Duplicature.type=single                         12.571429 13.2930514 24.4072524 2.070189e-08  -5.606031
## Horticultural.group=continuous flowering hybrid  3.243243  1.8126888 12.9009763 1.991959e-15  -7.941845
## Ploidy.level=4                                   7.720588 19.0332326 56.9037657 3.008180e-58 -16.089798
## Petal.colour=pink                                1.197605  2.4169184 46.5829847 1.197612e-92 -20.416316
## 
## $`2`
##                                        Cla/Mod    Mod/Cla    Global      p.value     v.test
## Petal.colour=pink                    34.730539 97.8902954 46.582985 3.538904e-80  18.961646
## Duplicature.type=NA                  38.764045 58.2278481 24.825662 3.955427e-34  12.180358
## Ploidy.level=4                       24.632353 84.8101266 56.903766 2.072723e-23   9.969499
## Duplicature.type=half-double         34.934498 33.7552743 15.969317 5.164805e-14   7.527684
## Horticultural.group=damas            39.436620 11.8143460  4.951185 2.221324e-06   4.732170
## Breeding.period=[1850-1859]          32.291667 13.0801688  6.694561 8.117603e-05   3.940901
## Geographic.origin=eastern europe     50.000000  4.2194093  1.394700 5.875389e-04   3.437303
## Geographic.origin=angers, france     25.949367 17.2995781 11.018131 1.365212e-03   3.201907
## Geographic.origin=paris, france      23.829787 23.6286920 16.387727 1.537470e-03   3.167517
## Horticultural.group=agathe           71.428571  2.1097046  0.488145 1.986351e-03   3.092265
## Ploidy.level=5                       40.740741  4.6413502  1.882845 2.739826e-03   2.995514
## Horticultural.group=wild             26.890756 13.5021097  8.298466 2.784066e-03   2.990626
## Breeding.period=[1810-1819]          27.586207 10.1265823  6.066946 7.303714e-03   2.682669
## Breeding.period=<1700                32.558140  5.9071730  2.998605 8.912812e-03   2.615380
## Breeding.period=[1820-1829]          25.609756  8.8607595  5.718271 3.075519e-02   2.160226
## Repeat.flowering.level=low/medium    28.260870  5.4852321  3.207810 4.237177e-02   2.029850
## Geographic.origin=northern europe     9.848485  5.4852321  9.205021 2.429108e-02  -2.252494
## Breeding.period=[1910-1914]           0.000000  0.0000000  1.464435 2.186347e-02  -2.292731
## Petal.colour=orange                   0.000000  0.0000000  2.022315 5.013018e-03  -2.806196
## Geographic.origin=south east, france  2.173913  0.4219409  3.207810 2.436938e-03  -3.031064
## Horticultural.group=chinese           6.250000  2.5316456  6.694561 2.355611e-03  -3.041297
## Geographic.origin=orleans, france     0.000000  0.0000000  2.370990 1.987482e-03  -3.092097
## Horticultural.group=hybrid tea        5.000000  1.6877637  5.578801 1.664401e-03  -3.144378
## Geographic.origin=china               4.000000  1.2658228  5.230126 7.930765e-04  -3.355200
## Breeding.period=[1880-1889]           7.051282  4.6413502 10.878661 2.755709e-04  -3.637244
## Ploidy.level=3                        6.060606  3.3755274  9.205021 2.083921e-04  -3.708620
## Petal.colour=NA                       4.081633  1.6877637  6.834031 1.131840e-04  -3.860438
## Breeding.period=>1920                 1.562500  0.4219409  4.463040 1.098387e-04  -3.867762
## Geographic.origin=lyon, france        6.349206  5.0632911 13.179916 1.084374e-05  -4.399626
## Petal.colour=yellow                   0.000000  0.0000000  4.951185 1.884037e-06  -4.765481
## Breeding.period=[1900-1909]           4.137931  2.5316456 10.111576 1.811686e-06  -4.773370
## Horticultural.group=tea               1.123596  0.4219409  6.206416 1.242011e-06  -4.848815
## Petal.colour=mauve_purple             0.000000  0.0000000  5.857741 1.553730e-07  -5.246076
## Duplicature.type=single               5.142857  7.5949367 24.407252 5.047877e-13  -7.224004
## Petal.colour=white                    0.000000  0.0000000 14.644351 1.117232e-18  -8.822709
## Petal.colour=red                      0.000000  0.0000000 17.991632 2.903186e-23  -9.935976
## Ploidy.level=2                        2.823529  5.0632911 29.637378 1.777296e-24 -10.210618
## Duplicature.type=double               0.000000  0.0000000 34.728033 6.855314e-50 -14.850986
## 
## $`3`
##                                         Cla/Mod    Mod/Cla    Global      p.value     v.test
## Ploidy.level=3                       75.0000000 49.2537313  9.205021 1.711581e-66  17.225428
## Duplicature.type=single              36.5714286 63.6815920 24.407252 3.315094e-38  12.923545
## Geographic.origin=lyon, france       31.7460317 29.8507463 13.179916 1.033792e-11   6.801718
## Horticultural.group=chinese          31.2500000 14.9253731  6.694561 6.839268e-06   4.498630
## Geographic.origin=south east, france 36.9565217  8.4577114  3.207810 8.250240e-05   3.937012
## Horticultural.group=tea              29.2134831 12.9353234  6.206416 1.201841e-04   3.845750
## Breeding.period=>1920                26.5625000  8.4577114  4.463040 6.862828e-03   2.703428
## Horticultural.group=noisette         25.0000000  9.9502488  5.578801 7.135843e-03   2.690437
## Petal.colour=NA                      23.4693878 11.4427861  6.834031 9.099850e-03   2.608280
## Breeding.period=[1890-1899]          20.5882353 17.4129353 11.854951 1.205091e-02   2.510650
## Breeding.period=[1870-1879]          22.2222222 10.9452736  6.903766 2.160738e-02   2.297200
## Breeding.period=[1910-1914]          33.3333333  3.4825871  1.464435 2.472651e-02   2.245649
## Bush.height=medium/high              19.8717949 15.4228856 10.878661 3.216526e-02   2.142351
## Petal.colour=yellow                  22.5352113  7.9601990  4.951185 4.606604e-02   1.994788
## Ploidy.level=6                        3.0303030  0.4975124  2.301255 4.847782e-02  -1.973156
## Horticultural.group=interspé          0.0000000  0.0000000  1.673640 2.583121e-02  -2.228740
## Horticultural.group=damas             4.2253521  1.4925373  4.951185 7.633688e-03  -2.667856
## Horticultural.group=wild              4.2016807  2.4875622  8.298466 3.528376e-04  -3.573057
## Breeding.period=[1840-1849]           4.5112782  2.9850746  9.274756 2.512999e-04  -3.660932
## Breeding.period=[1810-1819]           2.2988506  0.9950249  6.066946 1.850574e-04  -3.738587
## Breeding.period=[1850-1859]           2.0833333  0.9950249  6.694561 5.199656e-05  -4.046467
## Petal.colour=white                    5.7142857  5.9701493 14.644351 4.852500e-05  -4.062620
## Geographic.origin=angers, france      4.4303797  3.4825871 11.018131 4.600113e-05  -4.075067
## Geographic.origin=paris, france       5.1063830  5.9701493 16.387727 2.560772e-06  -4.703230
## Horticultural.group=moss              0.9708738  0.4975124  7.182706 1.899770e-06  -4.763804
## Duplicature.type=half-double          2.1834061  2.4875622 15.969317 6.284950e-11  -6.536799
## Duplicature.type=double               3.8152610  9.4527363 34.728033 1.096018e-18  -8.824855
## Ploidy.level=4                        4.7794118 19.4029851 56.903766 1.141284e-31 -11.709367
## 
## $`4`
##                                                    Cla/Mod    Mod/Cla    Global      p.value     v.test
## Petal.colour=pink                               20.9580838 99.2907801 46.582985 5.080749e-49  14.716108
## Ploidy.level=2                                  27.5294118 82.9787234 29.637378 6.069085e-44  13.903059
## Duplicature.type=double                         17.0682731 60.2836879 34.728033 7.687539e-11   6.506591
## Horticultural.group=tea                         30.3370787 19.1489362  6.206416 1.929590e-08   5.618198
## Geographic.origin=south east, france            30.4347826  9.9290780  3.207810 7.632564e-05   3.955654
## Repeat.flowering.level=NA                       11.0197368 95.0354610 84.797768 8.328313e-05   3.934751
## Breeding.period=[1900-1909]                     19.3103448 19.8581560 10.111576 2.379106e-04   3.674934
## Geographic.origin=orleans, france               29.4117647  7.0921986  2.370990 1.240986e-03   3.229289
## Breeding.period=[1890-1899]                     16.4705882 19.8581560 11.854951 3.838106e-03   2.891170
## Breeding.period=[1880-1889]                     16.6666667 18.4397163 10.878661 4.679479e-03   2.828308
## Geographic.origin=america                       20.2702703 10.6382979  5.160391 5.501750e-03   2.776087
## Horticultural.group=NA                          12.8421053 43.2624113 33.124128 8.289652e-03   2.640039
## Petal.colour=orange                              0.0000000  0.0000000  2.022315 4.817624e-02  -1.975810
## Geographic.origin=NA                             4.2105263  2.8368794  6.624826 4.385491e-02  -2.015475
## Breeding.period=[1810-1819]                      3.4482759  2.1276596  6.066946 2.633605e-02  -2.221220
## Repeat.flowering.level=very high                 0.0000000  0.0000000  2.859135 1.347079e-02  -2.471074
## Breeding.period=<1700                            0.0000000  0.0000000  2.998605 1.088089e-02  -2.546501
## Repeat.flowering.level=low/medium                0.0000000  0.0000000  3.207810 7.894182e-03  -2.656562
## Geographic.origin=paris, france                  4.6808511  7.8014184 16.387727 2.000034e-03  -3.090227
## Breeding.period=[1860-1869]                      1.8181818  1.4184397  7.670851 7.896846e-04  -3.356385
## Petal.colour=yellow                              0.0000000  0.0000000  4.951185 5.290348e-04  -3.465608
## Horticultural.group=damas                        0.0000000  0.0000000  4.951185 5.290348e-04  -3.465608
## Duplicature.type=half-double                     3.9301310  6.3829787 15.969317 3.909477e-04  -3.546120
## Petal.colour=NA                                  1.0204082  0.7092199  6.834031 3.647424e-04  -3.564362
## Geographic.origin=angers, france                 2.5316456  2.8368794 11.018131 2.409634e-04  -3.671678
## Horticultural.group=moss                         0.9708738  0.7092199  7.182706 2.185046e-04  -3.696602
## Petal.colour=mauve_purple                        0.0000000  0.0000000  5.857741 1.270801e-04  -3.832050
## Breeding.period=[1840-1849]                      0.7518797  0.7092199  9.274756 9.338434e-06  -4.431951
## Horticultural.group=continuous flowering hybrid  1.0810811  1.4184397 12.900976 3.742787e-07  -5.081595
## Petal.colour=white                               0.0000000  0.0000000 14.644351 5.640877e-11  -6.552957
## Duplicature.type=single                          1.7142857  4.2553191 24.407252 2.013818e-11  -6.705018
## Petal.colour=red                                 0.0000000  0.0000000 17.991632 1.400924e-13  -7.396242
## Ploidy.level=4                                   0.0000000  0.0000000 56.903766 1.039359e-56 -15.868968
## 
## $`5`
##                                                    Cla/Mod    Mod/Cla    Global      p.value     v.test
## Ploidy.level=4                                  28.5539216 99.5726496 56.903766 5.160774e-63  16.755520
## Duplicature.type=single                         44.0000000 65.8119658 24.407252 1.261269e-50  14.964042
## Petal.colour=red                                37.2093023 41.0256410 17.991632 3.729792e-20   9.195579
## Horticultural.group=moss                        36.8932039 16.2393162  7.182706 1.408025e-07   5.264198
## Geographic.origin=angers, france                28.4810127 19.2307692 11.018131 4.211124e-05   4.095580
## Geographic.origin=paris, france                 25.5319149 25.6410256 16.387727 6.962526e-05   3.977563
## Petal.colour=mauve_purple                       33.3333333 11.9658120  5.857741 7.800181e-05   3.950458
## Horticultural.group=continuous flowering hybrid 25.9459459 20.5128205 12.900976 3.242435e-04   3.595119
## Horticultural.group=bourbon                     31.1688312 10.2564103  5.369596 8.906785e-04   3.322959
## Breeding.period=[1840-1849]                     26.3157895 14.9572650  9.274756 2.019662e-03   3.087326
## Breeding.period=[1850-1859]                     27.0833333 11.1111111  6.694561 5.619799e-03   2.769178
## Horticultural.group=hybrid tea                  26.2500000  8.9743590  5.578801 1.986097e-02   2.328964
## Repeat.flowering.level=very high                29.2682927  5.1282051  2.859135 3.535993e-02   2.104212
## Breeding.period=[1890-1899]                     11.1764706  8.1196581 11.854951 4.762407e-02  -1.980708
## Petal.colour=white                              11.4285714 10.2564103 14.644351 3.347827e-02  -2.126300
## Breeding.period=[1830-1839]                      7.6086957  2.9914530  6.415621 1.322032e-02  -2.477778
## Geographic.origin=lyon, france                  10.0529101  8.1196581 13.179916 9.351570e-03  -2.598927
## Ploidy.level=5                                   0.0000000  0.0000000  1.882845 7.763419e-03  -2.662189
## Horticultural.group=noisette                     6.2500000  2.1367521  5.578801 6.876712e-03  -2.702756
## Petal.colour=yellow                              5.6338028  1.7094017  4.951185 6.731213e-03  -2.709857
## Breeding.period=[1900-1909]                      8.2758621  5.1282051 10.111576 3.428783e-03  -2.926429
## Ploidy.level=6                                   0.0000000  0.0000000  2.301255 2.601059e-03  -3.011330
## Geographic.origin=america                        4.0540541  1.2820513  5.160391 1.074175e-03  -3.270342
## Repeat.flowering.level=NA                       14.8848684 77.3504274 84.797768 9.106482e-04  -3.316769
## Geographic.origin=asia                           0.0000000  0.0000000  3.207810 2.390536e-04  -3.673710
## Horticultural.group=wild                         3.3613445  1.7094017  8.298466 5.167838e-06  -4.557856
## Horticultural.group=tea                          0.0000000  0.0000000  6.206416 7.447056e-08  -5.380037
## Ploidy.level=3                                   0.7575758  0.4273504  9.205021 5.365731e-10  -6.208017
## Petal.colour=pink                                8.5329341 24.3589744 46.582985 2.761887e-14  -7.609017
## Ploidy.level=2                                   0.0000000  0.0000000 29.637378 1.828035e-40 -13.317636
## Duplicature.type=double                          0.0000000  0.0000000 34.728033 3.430294e-49 -14.742655
```
